# Oleic acid triggers metabolic rewiring of T cells poising them for T helper 9 differentiation

**DOI:** 10.1016/j.isci.2024.109496

**Published:** 2024-03-12

**Authors:** Nathalie A. Reilly, Friederike Sonnet, Koen F. Dekkers, Joanneke C. Kwekkeboom, Lucy Sinke, Stan Hilt, Hayat M. Suleiman, Marten A. Hoeksema, Hailiang Mei, Erik W. van Zwet, Bart Everts, Andreea Ioan-Facsinay, J. Wouter Jukema, Bastiaan T. Heijmans

**Affiliations:** 1 Molecular Epidemiology, Department of Biomedical Data Sciences, Leiden, the Netherlands; 2 Leiden University Center for Infectious Diseases (LUCID), Leiden, the Netherlands; 3 Department of Rheumatology Leiden University Medical Center, Leiden, the Netherlands; 4 Department of Medical Biochemistry, Amsterdam UMC, location University of Amsterdam, Amsterdam, the Netherlands; 5 Sequencing Analysis Support Core, Department of Biomedical Data Sciences, Leiden, the Netherlands; 6 Medical Statistics, Department of Biomedical Data Sciences, Leiden, the Netherlands; 7 Department of Cardiology, Leiden University Medical Center, Leiden, the Netherlands; 8 Netherlands Heart Institute, Utrecht, the Netherlands

**Keywords:** Physiology, Human metabolism, Immunology, Cell biology, Transcriptomics

## Abstract

T cells are the most common immune cells in atherosclerotic plaques, and the function of T cells can be altered by fatty acids. Here, we show that pre-exposure of CD4^+^ T cells to oleic acid, an abundant fatty acid linked to cardiovascular events, upregulates core metabolic pathways and promotes differentiation into interleukin-9 (IL-9)-producing cells upon activation. RNA sequencing of non-activated T cells reveals that oleic acid upregulates genes encoding key enzymes responsible for cholesterol and fatty acid biosynthesis. Transcription footprint analysis links these expression changes to the differentiation toward T_H_9 cells, a pro-atherogenic subset. Spectral flow cytometry shows that pre-exposure to oleic acid results in a skew toward IL-9^+^-producing T cells upon activation. Importantly, pharmacological inhibition of either cholesterol or fatty acid biosynthesis abolishes this effect, suggesting a beneficial role for statins beyond cholesterol lowering. Taken together, oleic acid may affect inflammatory diseases like atherosclerosis by rewiring T cell metabolism.

## Highlights

• Non-activated T cells upregulate metabolism-related genes in response to oleic acid
• The expression changes link to PU.1, a key transcription factor of T helper 9 cells
• Upon activation, pre-exposure leads to a skew toward interleukin-9-producing cells
• Inhibition of cholesterol or fatty acid biosynthesis abolishes this effect

## Graphical abstract

**Figure:**
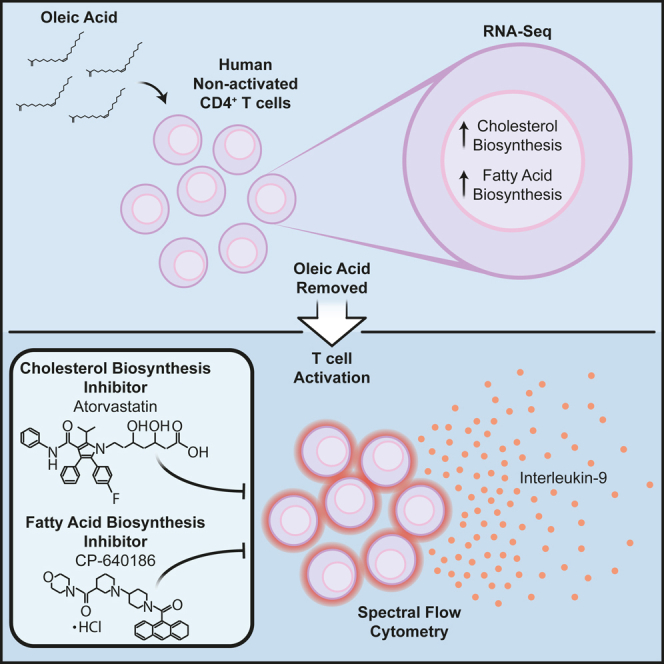

## Introduction

Atherosclerosis is the primary underlying cause of cardiovascular disease and is driven by the interactions between the immune system, lipids, and the vascular wall.^1^^,^^2^ Recent single-cell RNA sequencing (RNA-seq) and mass cytometry studies showed that T cells make up the majority of immune cells in atherosclerotic plaques, half of which are CD4^+^ T cells.^3^^,^^4^^,^^5^^,^^6^ This indicates that the role of CD4^+^ T cells in atherosclerosis is much greater than previously recognized.^7^^,^^8^^,^^9^ Lipids and in particular fatty acids are known to have a major influence on the function of CD4^+^ T cells.^2^ While previous research evaluated the effect of fatty acids on CD4^+^ T cell function during or after activation,^2^^,^^10^^,^^11^^,^^12^^,^^13^ interactions between fatty acids and CD4^+^ T cells relevant for atherosclerosis can already occur in the circulation, when the cells are in a non-activated state.^2^ While the impact of these interactions has not been studied, they may skew the differentiation toward pro- or anti-inflammatory subsets^8^^,^^14^ once the CD4^+^ T cells infiltrate atherosclerotic plaques or other disease sites such as rheumatoid arthritis and become activated.^2^^,^^15^

Fatty acids affect CD4^+^ T cells in multiple ways ranging from activation and proliferation to differentiation.^2^ It is thought that these effects are largely mediated by changes in metabolism.^16^ In a non-activated state, like in the circulation, CD4^+^ T cells rely on oxidative phosphorylation and β-oxidation of fatty acids for energy production.^17^^,^^18^ However, upon activation, CD4^+^ T cells switch their metabolism to fatty acid biosynthesis and aerobic glycolysis to support cell growth and proliferation, reminiscent of the Warburg effect.^18^^,^^19^^,^^20^ Importantly, the generation of specific T cell subset populations is associated with this metabolic reprogramming.^10^^,^^21^^,^^22^^,^^23^^,^^24^^,^^25^^,^^26^ The generally pro-inflammatory^14^^,^^27^^,^^28^ T helper 1 (T_H_1) and T helper 17 (T_H_17) cells, but also T helper 2 (T_H_2) cells that can be both pro- and anti-inflammatory, rely on pathways of aerobic glycolysis upon activation.^29^^,^^30^^,^^31^^,^^32^^,^^33^^,^^34^^,^^35^ In contrast, the generally anti-inflammatory regulatory T (T_reg_) cells mainly remain reliant on oxidative phosphorylation even after activation, indicating that the metabolic state of the cell may influence T cell effects in disease.^18^^,^^22^^,^^23^^,^^34^ Therefore, fatty acid-mediated metabolic reprogramming of CD4^+^ T cells may affect the initiation and progression of atherosclerosis by skewing CD4^+^ T cells toward a pro- or anti-inflammatory phenotype.

In this study, we characterized the effects of oleic acid on non-activated CD4^+^ T cell function. Oleic acid is a monounsaturated fatty acid that is of particular interest since it is one of the most abundant fatty acids in the circulation,^36^ is independently associated with an increased risk of cardiovascular events,^37^^,^^38^ and has been reported to elicit both pro- and anti-inflammatory effects on CD4^+^ T cells.^10^^,^^11^^,^^12^^,^^39^^,^^40^^,^^41^^,^^42^^,^^43^ To do so, we performed RNA-sequencing on non-activated CD4^+^ T cells exposed to oleic acid at 5 different time points. Furthermore, we performed spectral cytometry post-activation for various CD4^+^ T cell markers. We find that oleic acid exposure leads to a metabolic reprogramming and generates a profile that becomes skewed toward T_H_2, T_H_17, and, notably, T_H_9 CD4^+^ T cells after activation. This skewed profile post-activation is blocked by the addition of metabolic inhibitors during the initial oleic acid exposure.

## Results

### Establishing a model to study the effect of oleic acid on non-activated CD4^+^ T cells

Prior to studying the effect of oleic acid on non-activated CD4^+^ T cells, we evaluated various experimental conditions in order to establish an *in vitro* exposure model. Cells were cultured in medium containing fetal calf serum (FCS) to ensure cell viability during culture, and oleic acid was complexed to BSA to model physiological conditions of the circulation. The cellular response to oleic acid was assessed by measuring cell viability and the expression of *CPT1A*. The *CPT1A* gene encodes the long-chain fatty acid transporter carnitine palmitoyl transferase 1a, a rate-limiting enzyme in the metabolic process of β-fatty acid oxidation. First, three different types of culturing conditions for non-activated CD4^+^ T cells were compared: 5% FCS medium with oleic acid bound to fatty acid-free (FAF) BSA, 5% FCS medium with oleic acid diluted in 5% FCS medium, and FAF medium with oleic acid bound to FAF BSA. However, the latter two conditions led to either undissolved oleic acid or a low cell viability (Figure S1A). The first condition produced the largest *CPT1A* response while maintaining a high cell viability (Figure S1), presumably because oleic acid bound to BSA and the presence of FCS may be a better approximation of physiological conditions. In addition, various oleic acid concentrations used in previous studies were evaluated.^10^^,^^11^^,^^12^^,^^42^^,^^43^^,^^44^^,^^45^^,^^46^ A concentration of 30 μg/mL was observed to result in the highest *CPT1A* upregulation (9.83-fold, SE 5.60) while maintaining cell viability (84.36%, SE 0.49%; Figure S1). Importantly, this concentration is lower than the typical oleic acid concentration in the human circulation (85–904 μg/mL).^47^ The solvent control, ethanol, did not influence the results and was thus used as the control condition for the following analyses (Figure S2). Finally, we measured the oleic acid concentration in the medium due to the addition of 5% FCS. This concentration was 0.26 μg/mL of free oleic acid and 4.39 μg/mL oleic acid as components of larger molecules including cholesterol esters and sphingolipids.

### Transcriptomic analysis of oleic acid-exposed non-activated CD4^+^ T cells

**Figure 1:**
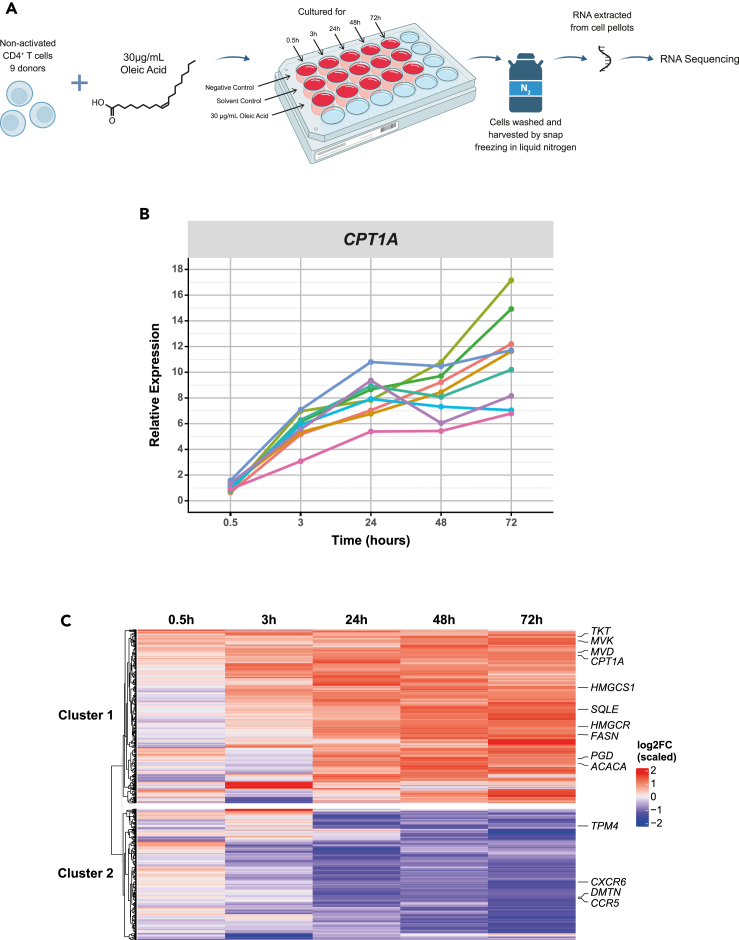
Oleic acid exposure in non-activated CD4^+^ T cells induces changes in transcriptomics (A) Experimental setup for RNA sequencing of oleic acid-exposed non-activated CD4^+^ T cells, n = 9. (B) Line plot showing the relative expression of *CPT1A* per donor across time as a confirmation of the *in vitro* model by RT-qPCR. Values are colored by donor across time. On average *CPT1A* was upregulated 1.03 SE 0.10-fold at 0.5 h, 5.73 SE 0.40-fold at 3 h, 8.08 SE 0.53-fold at 24 h, 8.39 SE 0.62-fold at 48 h, and 11.09 SE 1.16-fold at 72 h as compared to the solvent control, n = 9. (C) Differentially expressed genes (DEGs) in oleic acid-exposed non-activated CD4^+^ T cells across time as compared to the solvent control. Heatmap obtained from the DESeq2 analysis resulting in 544 DEGs (P_FDR_ < 0.05). DEGs were plotted across time to show the genes expression as log2FoldChange at each time point. Unsupervised K-means clustering indicated 2 clusters. Cluster 1 contains 310 of the DEGs, which are generally upregulated and are represented in red, and cluster 2 contains 234 of the DEGs, which are generally downregulated and are represented in blue. Genes of interest are labeled, n = 9.

In order to identify the molecular features that define the effect of oleic acid exposure on non-activated CD4^+^ T cells *in vitro*, we exposed non-activated CD4^+^ T cells to 30 μg/mL oleic acid for 0.5, 3, 24, 48, or 72 h (n = 9; Figure 1A). First, we measured *CPT1A* expression and found that its expression consistently increased over time indicating a robust response to oleic acid exposure across donors, while *CPT1A* expression did not change under control conditions (Figures 1B and S2B). Next, we analyzed the transcriptome of non-activated CD4^+^ T cells after oleic acid exposure using RNA-seq. Oleic acid induced differential expression of 544 genes (P_FDR_ < 0.05) that clustered into 310 upregulated genes and 234 downregulated genes (Figures 1C and S3, and Tables S1A and S1B). There was no statistical evidence for further subdivisions of the two clusters, for example, in fast- and slow-responding genes.

**Figure 2:**
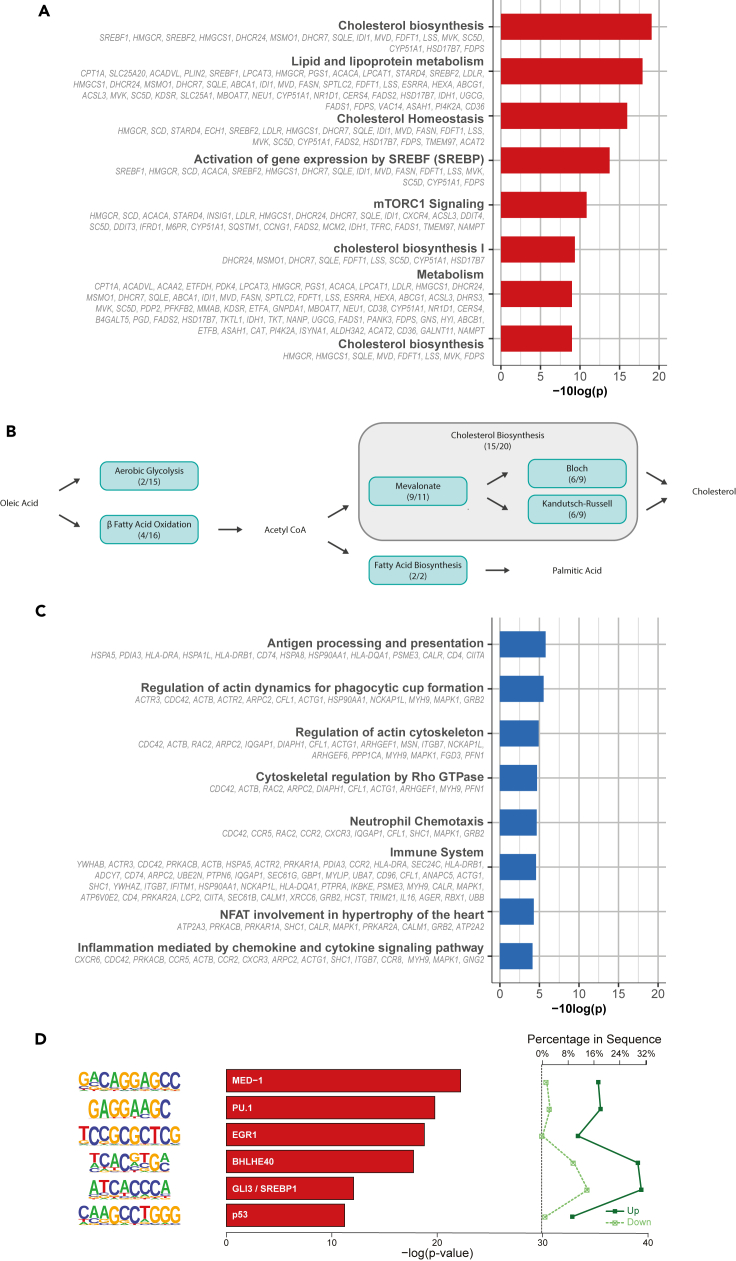
Up- and downregulated pathways and transcription factors in oleic acid-exposed non-activated CD4^+^ T cells (A) Pathway enrichment analysis of cluster 1 DEGs generated using *clusterProfiler* using 10 human pathway databases. Top 8 enrichments are shown. (B) Illustration of canonical pathway map of oleic acid metabolism by non-activated CD4^+^ T cells exposed to oleic acid. Blue boxes indicate metabolic pathways with the number of genes present in that particular pathway from cluster 1 of the RNA sequencing. Cholesterol biosynthesis can be divided into 3 separate pathways indicated by the surrounding gray rectangle. (C) Pathway enrichment analysis of cluster 2 DEGs generated using *clusterProfiler* using 10 human pathway databases. Top 8 enrichments are shown. (D) *De novo* motif analysis on promoters of up- versus down-regulated genes. Enrichment of transcription factor binding motifs was performed using HOMER. 6 motifs are shown with supplementing information on p value, percentage of genes in upregulated gene set and percentage of genes in downregulated gene set, transcription factor name, -log(p value), and percentage in sequence.

We first examined the functions of the 310 genes that were upregulated in non-activated CD4^+^ T cells by oleic acid exposure. We inspected the top differentially expressed genes (Figure S3A and Table S1A). The top differentially expressed gene was *CPT1A* highlighting the involvement of β-fatty acid oxidation. In addition, we found an increased expression of *HMGCR* (3-hydroxy-3-methyl-glutaryl-coenzyme A [CoA] reductase), encoding the rate-limiting enzyme for cholesterol biosynthesis, and *ACACA* (acetyl-coenzyme A carboxylase 1), encoding the rate-limiting enzyme of fatty acid biosynthesis. Furthermore, transcripts of several aerobic glycolysis-related genes, such as *TKT* and *PGD*, were upregulated (Figure S3A and Table S1A). A formal analysis of enriched biological processes among all 310 upregulated genes confirmed the involvement of metabolism. In particular, cholesterol biosynthesis (P_FDR_ < 0.001), homeostasis (P_FDR_ < 0.001), and signaling of mTORC1 (P_FDR_ < 0.001), a key complex of mechanistic target of rapamycin (mTOR) which aids in the switch toward aerobic glycolysis and fatty acid biosynthesis, were enriched (Figure 2A). Mapping the upregulated genes to canonical metabolic pathways further supported a specific metabolic rewiring of oleic acid-exposed non-activated CD4^+^ T cells (Figure 2B). First, oleic acid can first be catabolized through beta oxidation to produce acetyl-CoA, which can then be used as a starting point for cholesterol and fatty acid biosynthesis. In addition to *CPT1A*, we found 4 out of 15 enzymes in β-fatty acid oxidation (including *SLC25A20*, *ACADVL*, and *ACAA2*) and 2 out of 15 enzymes in the aerobic glycolysis pathway to be upregulated (*TKT* and *PGD*; Figures 2B, S4, and S5). Remarkably, on top of *HMGCR*, 15 out of 20 enzymes involved in cholesterol biosynthesis were upregulated in our gene set, including several key rate-limiting genes (such as *HMGCS1*, *SQLE*, *MVD*, and *MVK*). More specifically, 9/11 components of the mevalonate, 6/9 of the Bloch, and 6/9 of the Kandutsch-Russell pathway, together responsible for cholesterol biosynthesis, were upregulated (Figure 2B and S6). The upregulated gene set also included *ACACA* and *FASN* that encode the two enzymes that together are responsible for the 37 reactions making up fatty acid biosynthesis (Figures 2B and S7). Of note, the genes *ACACA* and *FASN* have been implicated in the differentiation toward T_H_17 cells, a highly pro-inflammatory subset of CD4^+^ T cells.^46^ Furthermore, aerobic glycolysis and cholesterol and fatty acid biosynthesis are the hallmark metabolic processes of activated T cells and suggest that non-activated CD4^+^ T cells undergo a metabolic reprogramming upon oleic acid exposure that may poise the cells for a different response to activation.

We then examined the functions of the 234 genes that were downregulated in non-activated CD4^+^ T cells by oleic acid exposure. We first inspected the top differentially expressed genes (Figure S3B and Table S1B). Among the top downregulated genes, decreased expression of *CXCR6* and *CCR5*, important chemokine receptors in the T cell immune response, was measured. Moreover, expression of *TPM4*, encoding actin-binding proteins involved in the cytoskeleton, and *DMTN*, encoding an actin-binding and bundling protein that stabilizes the actin cytoskeleton, was also downregulated. A formal analysis of the enriched biological processes among all 234 downregulated genes revealed a wide variety of different pathways. In line with the genes observed among the top downregulated genes, this included processes involved in immune response (*CCR2*, *CCR8, HLA-DRA*, *SLC2A1*) (P_FDR_ < 0.001) and actin cytoskeleton organization (*ACTB*, *RAC2*, *ARPC2*, *IQGAP1*) (P_FDR_ < 0.001) (Figure 2C). In addition, processes involved in chemotaxis (P_FDR_ < 0.001), chemokine and cytokine signaling (P_FDR_ < 0.001), and Rho GTPase regulation (P_FDR_ < 0.001) were also downregulated (Figure 2C). Overall, these data point to a broad yet specific downregulation of genes in oleic acid-exposed non-activated CD4^+^ T cells, perhaps to cope with the influx of the fatty acid.

Next, we investigated whether specific transcription factors may underlie the differential expression observed by testing the enrichment of transcription factor binding motifs in upregulated vs. downregulated genes. The top motifs enriched among upregulated genes included key transcription factors PU.1, EGR1, BHLHE40, and SREBP1 (Figure 2D). Notably, PU.1 is the key transcription factor for the development of T_H_9 cells. BHLHE40 has been linked to T_H_17 development and pathogenicity in autoimmune encephalomyelitis suggesting an additional possible preference toward T_H_17 differentiation post-activation.^48^^,^^49^ Furthermore, EGR1 and SREBP1 are involved in either the activation of Tbet or fatty acid and cholesterol biosynthesis, respectively.^50^^,^^51^ These data further support the notion that oleic acid-exposed non-activated CD4^+^ T cells may be poised to differentiate toward T_H_9 and T_H_17 T cell subsets after activation.

### Oleic acid induced CD4^+^ T cell phenotypes after activation

**Figure 3:**
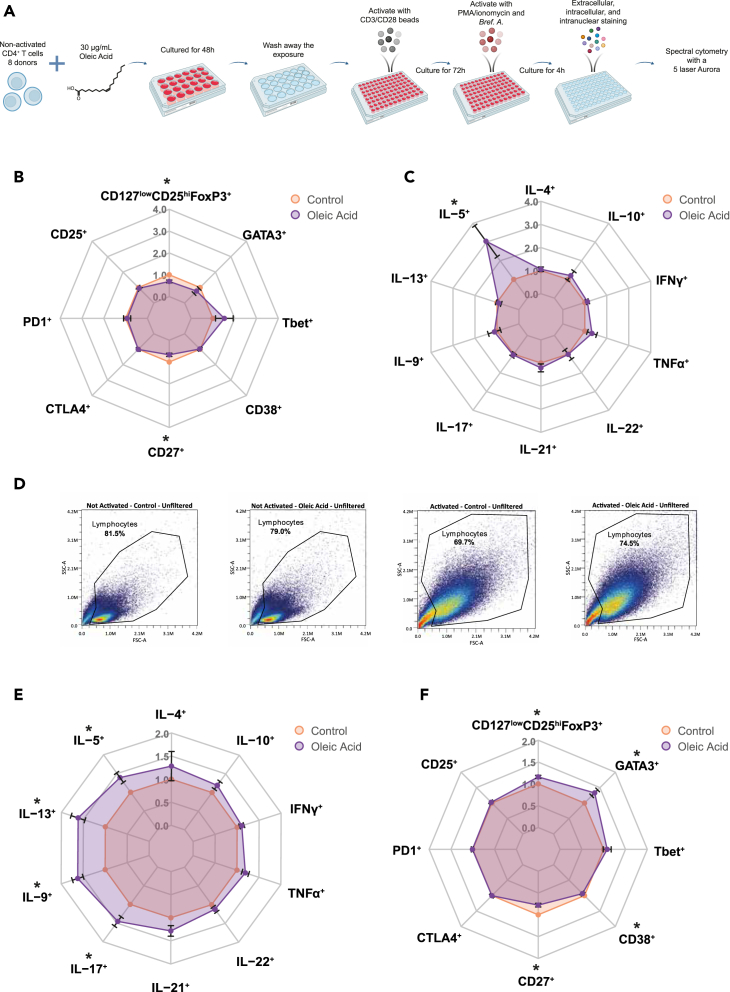
Oleic acid pre-exposure leads to changes in expression of extracellular markers, transcription factors, and intracellular cytokines (∗) P_FDR_ < 0.05, n = 8. (A) Experimental setup for spectral cytometry measurements of oleic acid-exposed non-activated CD4^+^ T cells for 48 h with and without activation for 72 h post-exposure. (B) Radar plot of various CD4^+^ T cell external markers and transcription factors expressed in CD4^+^ T cells after 48 h of oleic acid exposure or control followed by 72 h of rest and 4 h stimulus with PMA/ionomycin. Values are expressed as fold change and standard error relative to control. (C) Radar plot of various CD4^+^ T cell internal cytokines expressed in CD4^+^ T cells after 48 h of oleic acid exposure or control followed by 72 h of rest and 4 h stimulus with PMA/ionomycin. Values are expressed as fold change and standard error relative to control. (D) Forward and side scatter of activated vs. non-activated and control vs. oleic acid pre-exposed CD4^+^ T cells. Large differences in cell shape between the non-activated and activated state were observed, but little difference in cell shape between pre-exposure to control or oleic acid was found. Non-activated control-exposed cells are on the far left, non-activated oleic acid-exposed cells are on the center left, activated control-exposed cells are on the center right, and activated oleic acid-exposed cells are on the far right. (E) Radar plot of various CD4^+^ T cell internal cytokines expressed in CD4^+^ T cells after 48 h of oleic acid exposure or control followed by 72 h of activation with CD3/CD28 activation beads and 4 h additional stimulus with PMA/ionomycin. Values are expressed as fold change and standard error relative to control. (F) Radar plot of various CD4^+^ T cell external markers and transcription factors expressed in CD4^+^ T cells after 48 h of oleic acid exposure or control followed by 72 h of activation with CD3/CD28 activation beads and 4 h additional stimulus with PMA/ionomycin. Values are expressed as fold change and standard error relative to control.

To determine the functional impact of the transcriptomic changes identified, we characterized the phenotypes of CD4^+^ T cells that were pre-exposed to oleic acid or control conditions and subsequently activated in the absence of oleic acid. To this end, non-activated CD4^+^ T cells of 8 out of 9 donors, for whom sufficient cells were available, were again exposed to 30 μg/mL oleic acid (Figure 3A). The effect of exposure was confirmed by an upregulation of *CPT1A* (Figure S8A); cell viability was high (>90%), and there was no difference in diameter between cells exposed to oleic acid and control (Figures S8B–S8E).

First, we examined phenotypes after oleic acid exposure without activation (Figure S9 and Table S1C). We observed decreased frequencies of CD127^low^CD25^hi^FoxP3^+^ and CD27^+^ CD4^+^ T cells in response to oleic acid pre-exposure (P_FDR_ < 0.05; Figure 3B). In non-activated cells, the CD127^low^CD25^hi^FoxP3^+^ population is representative of T_reg_ cells, and thus the decreased frequencies in the non-activated cells are in line with the lower *FOXP3* expression observed in the RNA-seq analysis. Increased frequencies of interleukin (IL)-5^+^ cells were also observed (P_FDR_ < 0.05; Figure 3C) These data suggest that the oleic acid-induced changes in gene expression are reflected in consistent functional characteristics of the CD4^+^ T cells without activation.

Activation of the CD4^+^ T cells led to an increased cell size irrespective of pre-exposure to oleic acid (Figure 3D). In contrast, the expression of surface and intracellular markers was influenced by exposure to oleic acid prior to activation (Figure S10 and Table S1D). Pre-exposure to oleic acid resulted in a higher proportion of IL-9^+^ cells (P_FDR_ < 0.01) as compared to the control (Figure 3E). Additional analysis showed that IL-9 was not co-expressed with other T_H_2-associated cytokines (Table S1E). This aligns with our finding that a large percentage of upregulated genes mapped to a PU.1 motif (Figure 2D), the key transcription factor controlling T_H_9 differentiation. Furthermore, increased frequencies of IL-17A^+^ cells were observed after pre-exposure to oleic acid as compared with control conditions (P_FDR_ < 0.05). As IL-17A is mainly produced by T_H_17 cells, it was hypothesized that other T_H_17-associated cytokines, such as IL-21, may also have been upregulated. Indeed, IL-21^+^ cells were increased in frequency (p < 0.05), but this effect was no longer significant after correction for multiple testing (P_FDR_ < 0.08). This aligns with our finding that a large percentage of upregulated genes mapped to the BHLHE40 motif (Figure 2D) involved in T_H_17 differentiation.^48^^,^^49^ Activated CD4^+^ T cells showed increased frequencies of CD127^low^CD25^hi^FoxP3^+^ and GATA3^+^ and decreased frequencies of CD27^+^ and CD38^+^ cells in response to oleic acid pre-exposure (P_FDR_ < 0.05; Figure 3F). However, FoxP3 can be expressed on activated conventional T cells without a suppressor function;^52^ therefore, we are unable to differentiate whether the increased proportion of CD127^low^CD25^hi^FoxP3^+^ cells post-activation is due to increased differentiation toward T_reg_ or an artifact of T cell activation. GATA3 is the key transcription factor involved in T_H_2 differentiation, and, as such, frequencies of T_H_2-related cytokines IL-5^+^ and IL-13^+^ were increased (P_FDR_ < 0.05; Figure 3E). Finally, we observed that the effect of oleic acid on differentiation is not secondary to a differential proliferative capacity (p > 0.92; Figure S11). Together, these data indicate that the metabolic changes in non-activated CD4^+^ cells upon oleic acid exposure skew the cells toward producing more cytokines characteristic of T_H_9, T_H_17, and T_H_2 subsets upon activation.

In order to reinforce our findings, we repeated the spectral cytometry analysis with 8 independent donors. The effect of oleic acid exposure was confirmed by an upregulation of *CPT1A* (Figure S12A). Cell viability was high (>78%), and there was no difference in diameter between cells exposed to oleic acid and control (Figure S12B–S12E). Without activation, the phenotypes of oleic acid-exposed CD4^+^ T cells showed increased frequencies of both IL-17A^+^ (P_FDR_ < 0.05) and TNFα^+^ cells (P_FDR_ < 0.05; Figures S13A, S13B, and S14; Table S1F). After activation, the phenotypes of oleic acid-exposed CD4^+^ T cells showed an increased frequency of IL-9^+^ (P_FDR_ < 0.05) and GATA3^+^ (P_FDR_ < 0.05) cells as well as decreased frequencies of CD38^+^ cells (P_FDR_ < 0.05; Figures S13C, S13D, and S15, and Table S1G). These findings in non-activated and activated cells confirm results of our experiment and substantiate that oleic acid exposure in non-activated CD4^+^ cells poised the cells toward producing more cytokines representative of T_H_9 cells post-activation.

### Oleic acid induced CD4^+^ T cell phenotypes blocked by metabolic inhibitors

We next determined whether induction of this profile, reminiscent of an increase differentiation toward T_H_9, T_H_17, and T_H_2 subsets, was dependent on an upregulation of cholesterol and fatty acid biosynthesis in line with our RNA-seq data. We inhibited cholesterol synthesis with atorvastatin, targeting 3-hydroxy-3-methylglutaryl (HMG)-CoA reductase (*HMGCR*), and fatty acid synthesis with CP-640186, targeting both ACC1 and ACC2 (*ACACA* and *ACACB*). To this end, non-activated CD4^+^ T cells of 3 out of 8 donors, for whom sufficient cells were available, were again exposed to control conditions, oleic acid only, oleic acid +10 μM atorvastatin, oleic acid +20 μM CP-640186, or oleic acid and both atorvastatin and CP-640186 for 48 h. The effect of oleic acid exposure was confirmed by an upregulation of *CPT1A* (Figure S16A). Cell viability was high (>88%), and there was no difference in diameter between cells exposed to control, oleic acid, or oleic acid + inhibitors (Figure S16B–S16E).

**Figure 4:**
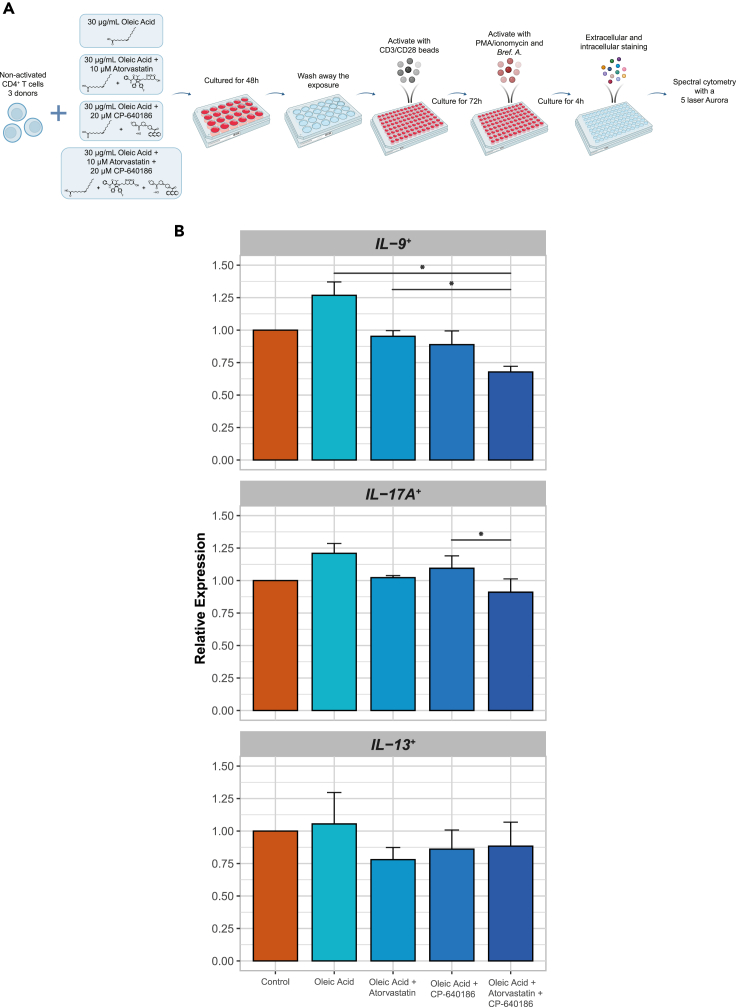
Metabolic inhibitors prevent oleic acid pre-exposure-induced changes in expression of IL-9, IL-17A, and IL-13 (∗) p < 0.05, n = 3. (A) Experimental setup for spectral cytometry measurements of oleic acid + inhibitor exposed non-activated CD4^+^ T cells for 48 h with activation for 72 h post-exposure. (B) Bar plot of IL-9, IL-17A, and IL-13 expression in CD4^+^ T cell after 48 h of control, oleic acid, oleic acid + atorvastatin, oleic acid + CP-640186, or oleic acid + atorvastatin + CP-640186 exposure followed by 72 h of activation with CD3/CD28 activation beads and 4 h additional stimulus with PMA/ionomycin. Values are expressed as fold change and standard error relative to control.

Subsequently, both oleic acid and the inhibitors were washed away and the pre-exposed CD4^+^ T cells were activated. We evaluated the expression of one key marker for each subset: IL-9 for T_H_9, IL-17A for T_H_17, and IL-13 for T_H_2 cells (Figures 4A and S17). Remarkably, the ability of oleic acid to increase frequencies of IL-9^+^ cells was inhibited by both atorvastatin and CP-640186 (Figure 4B and Table S1H). Although similar trends were observed for frequencies of IL-17A^+^ and IL-13^+^ cells, these effects were not statistically significant (Figure 4B). These data indicate that oleic acid promotes the differentiation to in particular IL-9^+^-producing T cells via upregulation of cholesterol and fatty acid biosynthesis.

## Discussion

T cells are known to respond to fatty acids.^2^ Using an *in vitro* model, we show that sub-physiological concentrations of oleic acid can already influence CD4^+^ T cells when in a non-activated state by upregulating the expression of genes that encode enzymes involved in core metabolic pathways responsible for cholesterol biosynthesis, fatty acid biosynthesis, and aerobic glycolysis. These metabolic processes are hallmarks of activated T cells.^53^ Indeed, upon activation, CD4^+^ T cells pre-exposed to oleic acid are characterized by increased production of cytokines, including IL-9, IL-17A, IL-5, and IL-13, indicative of a preferential differentiation toward the pro-inflammatory T helper subsets T_H_9 as well as T_H_17 and T_H_2, which can have both pro- and anti-inflammatory effects. Interestingly, this effect is abolished in particular for IL-9^+^-producing cells by blocking the cholesterol or the fatty acid biosynthesis pathways during the initial exposure to oleic acid. Our findings imply that increased fatty acid levels in the circulation can rewire the metabolism of non-activated T cells and poise them to particularly differentiate toward T_H_9 cells, for example, when the cells infiltrate diseased tissues, including atherosclerotic plaques, and become activated.

Our results showed that cholesterol biosynthesis was the primary transcriptionally upregulated pathway in oleic acid-exposed non-activated CD4^+^ T cells (15 out of 20 genes). This upregulation is of particular interest because of this pathway’s role in producing the necessary metabolites required for T cell activation.^54^ Cholesterol biosynthesis is upregulated in activated T cells to support membrane production, cell signaling through the formation of lipid rafts, and prenylation of signaling proteins.^55^ Additionally, intracellular cholesterol sensing has also been found to play a role in T cell differentiation, particularly toward pro-inflammatory subsets. For example, sterols were found to bind the T_H_17 transcription factor RORγt and could promote its activity.^56^ Thus, the upregulation of gene expression in the cholesterol biosynthesis pathway due to oleic acid exposure may be indicative of a metabolic reprogramming of the non-activated CD4^+^ T cells toward an activated state and may lead to the differentiation toward pro-inflammatory subsets post-activation.

Additionally, expression of the two genes comprising the *de novo* fatty acid biosynthesis pathway was upregulated (*ACACA* and *FASN*). Together, cholesterol and fatty acid biosynthesis comprise part of the process known as lipogenesis, the synthesis of novel lipids in a cell. Lipogenesis is induced by the activation of the transcription factor SREBP1, which was associated with the upregulated transcripts in our RNA-seq data. Enrichment analysis of our transcripts also revealed upregulated genes in mTORC1 signaling, which is known to induce the activation of SREBP1.^57^ Although this effect is usually insulin dependent, obesity and overfeeding have been shown to hyperactivate mTORC1.^58^ Thus, it is possible that oleic acid alone could induce the activation of mTORC1, which in turn activates SREBP1, leading to lipogenesis and expression of cholesterol and fatty acid biosynthesis-related genes.

Fatty acid biosynthesis has also been related to the development of T_H_17 cells.^17^^,^^32^ Specifically, the mRNA expression of genes *ACACA*, encoding for acetyl-CoA carboxylase 1 (ACC1), and *FASN*, encoding fatty acid synthase, was increased in our dataset. These genes are key determinants in the development of the pro-inflammatory subset T_H_17 cells over the anti-inflammatory subset T_reg_ cells.^22^^,^^31^^,^^39^^,^^46^^,^^59^ Correspondingly, *FOXP3*, the key transcription factor of T_reg_ cells, was downregulated in oleic acid-exposed non-activated CD4^+^ T cells. Upregulated transcripts were found to be associated with the transcription factor PU.1. PU.1 is the key transcription factor in the development of T_H_9 cells. This subset is a highly pro-inflammatory subset related to T_H_2 cells.^60^ This further supports the idea that oleic acid exposure leads to a cellular metabolic reprogramming that could promote the development of pro-inflammatory T cell subsets, specifically T_H_9, and possibly also T_H_17 and T_H_2 cells. These results indicate that oleic acid-exposed non-activated CD4^+^ T cells were upregulating genes involved in metabolism to initiate/prepare for the selective differentiation into T_H_9/T_H_17/T_H_2 cells post-activation. Moreover, the metabolic processes being enhanced due to oleic acid exposure hint that the cells may preferentially differentiate toward T_H_9, T_H_17, and T_H_2 cells upon activation.

Importantly, we provide evidence that the oleic acid-induced metabolic rewiring underpins the observed enhanced T_H_9, T_H_17, and T_H_2 differentiation as exposing non-activated CD4^+^ T cells to oleic acid in combination with cholesterol or fatty acid synthesis inhibitors decreased the frequencies of IL-9^+^, IL-17A^+^, and IL-13^+^ cells. While the role of T_H_17 and T_H_2 cells in atherosclerosis has not been resolved, these cell types have been identified as pro-inflammatory in other diseases such as autoimmune encephalomyelitis and allergy, respectively.^35^^,^^61^ In contrast, T_H_9 cells have been implicated in atherosclerosis pathogenesis.^62^^,^^63^^,^^64^ Additionally, statins have been hypothesized to have protective effects independent of cholesterol reduction;^65^ our study hints that effect of statins on T cell responses could contribute to this protective role.

Immune-lipid interactions occur in the circulation, which is a complex environment comprising many factors that can affect T cell function prior to their recruitment to disease site like the atherosclerotic plaque.^66^ Fatty acids are a significant component of this environment and have been found to exert their effect not only on atherosclerosis but also on T cell function.^2^ Our model was designed to determine the effect of oleic acid exposure on non-activated CD4^+^ T cells. Here, we focus solely on the interaction between oleic acid and CD4^+^ T cells and thus make no claim to what effects this fatty acid might have in relation to atherosclerotic cardiovascular disease as a component of more complex lipids, like olive oil. Our study only focuses on oleic acid as it was shown to have both pro- and anti-inflammatory effects on T cells in previous studies.^10^^,^^11^^,^^12^^,^^39^^,^^40^^,^^41^^,^^42^^,^^43^ Circulating levels of oleic acid have been found to be related to pro-atherogenic effects,^37^^,^^38^ and oleic acid is one of the most abundant fatty acids in the human circulation.^36^ However, this does not preclude any effects *in vivo* or of other types of fatty acids on non-activated T cells.

Taken together, our results suggest that oleic acid can rewire the metabolism of non-activated CD4^+^ T cells, as they exist in the circulation. This metabolic rewiring induces a preferential differentiation in particular toward T_H_9 cell types following activation. Since T_H_9 cell have pro-atherogenic effects^62^^,^^63^^,^^64^ and we show that the oleic acid-induced differentiation into T_H_9 cells can be inhibited by statins, our study indicates a new route by which fatty acids can contribute to atherosclerosis through modifiable effects on the immune system.

### Limitations of the study

Although our experiments show that non-activated CD4^+^ T cells exposed to oleic acid undergo distinct changes in the expression of genes encoding key enzymes constituting core metabolic pathways, and that subsequent activation of pre-exposed cells results in a differentiation that is skewed toward IL-9^+^- producing T cells, our study used an *in vitro* model to establish these relationships and lacked an in-depth functional and mechanistic characterization of the metabolic changes involved. First, studies *in vivo* will be required to determine the relevance of our findings to the etiology of inflammatory diseases including atherosclerosis. Second, additional functional support for the occurrence of metabolic rewiring by oleic acid as implied by our results will be important. However, it will be challenging to assay functional effects. The T cells exposed to oleic acid were in a non-activated state and hence are unlikely to display functional differences in cell metabolism. Metabolic pathways are involved in the differentiation of CD4^+^ T cells into specific subsets, and functional metabolic differences in T cells generally emerge only post-activation. Cell-subtype-specific and single-cell approaches can be informative to overcome the limitations of the bulk sequencing and spectrometry experiments as we performed in this study,^18^ including flow cytometry-based methods to functionally profile energy metabolism,^67^ mass spectrometry, and proteomics. Nevertheless, pharmacological inhibition of fatty acid and cholesterol metabolism in non-activated T cells abolished the oleic acid-induced skew toward IL-9^+^-producing T cells upon activation, supporting our overall interpretation that metabolism is mechanistically involved in the effects we observed.

## STAR★Methods

### Key resources table

**Table:** 

REAGENT or RESOURCE	SOURCE	IDENTIFIER
**Antibodies**		

Anti-human CD4 MicroBeads - lyophilized	Miltenyi Biotec	Cat#130-097-048; RRID:AB_2889919
Anti-human CD3; PE; Isotype Mouse; Clone SK7	BD Biosciences	Cat#345765; RRID:AB_2868796
Anti-human CD4; APC; Isotype Mouse; Clone SK3	BD Biosciences	Cat#345771; RRID:AB_2868799
Anti-human CD8; FITC; Isotype Mouse; Clone HIT8a	BD Biosciences	Cat#555634; RRID:AB_395996
Anti-human CD14; PEcy7; Isotype Mouse; Clone M5E2	BD Biosciences	Cat#557742; RRID:AB_396848
Dynabeads™ Human T-Activator CD3/CD28 for T Cell Expansion and Activation	Thermo Fisher Scientific	Cat#11161D; RRID:AB_2916088
Anti-human CD38; APC/Fire™ 810; Isotype Mouse; Clone HB-7	BioLegend	Cat#356643; RRID:AB_2860936
Anti-human CD8; Pacific Orange™; Isotype Mouse; Clone 3B5	Thermo Fisher Scientific	Cat#MHCD0830; RRID:AB_10372066
Anti-human CD25; BUV563; Isotype Mouse; Clone 2A3	BD Biosciences	Cat#612918; RRID:AB_2870203
Anti-human CD45RA; BUV496; Isotype Mouse; Clone 5H9	BD Biosciences	Cat#741182; RRID:AB_2870749
Anti-human CD45RO; BUV805; Isotype Mouse; Clone UCHL1	BD Biosciences	Cat#748367; RRID:AB_2872786
Anti-human CD4; cFluor® YG584; Isotype Mouse; Clone SK3	Cytek Biosciences	Cat#R7-20042
Anti-human CD3; BUV395; Isotype Mouse; Clone UCHT1	BD Biosciences	Cat#563546; RRID:AB_2744387
Anti-human CD27; APC-H7; Isotype Mouse; Clone M-T271	BD Biosciences	Cat#560222; RRID:AB_1645474
Anti-human CD279 (PD-1); BV750; Isotype Mouse; Clone EH12.1	BD Biosciences	Cat#747446; RRID:AB_2872125
Anti-human CD127; R718; Isotype Mouse; Clone HIL-7R-M21	BD Biosciences	Cat#566967; RRID:AB_2869977
Anti-human CD197 (CCR7); Brilliant Violet 785™; Isotype Mouse; Clone G043H7	BioLegend	Cat#353230; RRID:AB_2561371
Anti-human TNF; PE-Cy™7; Isotype Mouse; Clone MAb11	BD Biosciences	Cat#557647; RRID:AB_396764
Anti-human IL-17A; Pacific Blue™; Isotype Mouse; Clone BL168	BioLegend	Cat#512312; RRID:AB_961392
Anti-human IL-5; APC; Isotype Rat; Clone TRFK5	BioLegend	Cat#504306; RRID:AB_315329
Anti-human IL-4; BUV737; Isotype Rat; Clone MP4-25D2	BD Biosciences	Cat#612835; RRID:AB_2870157
Anti-human IFN-γ; BV650; Isotype Mouse; Clone 4S.B3	BD Biosciences	Cat#563416; RRID:AB_2738193
Anti-human IL-13; BV711; Isotype Rat; Clone JES10-5A2	BD Biosciences	Cat#564288; RRID:AB_2738731
Anti-human IL-9; PE; Isotype Mouse; Clone MH9A4	BioLegend	Cat#507605; RRID:AB_315487
Anti-human IL-10; PerCP-eFluor™ 710; Isotype Rat; Clone JES3-9D7	Thermo Fisher Scientific	Cat#46-7108-42; RRID:AB_2573833
Anti-human CD152; PE-Cy™5; Isotype Mouse; Clone BNI3	BD Biosciences	Cat#555854; RRID:AB_396177
Anti-human IL-21; Alexa Fluor® 647; Isotype Mouse; Clone 3A3-N2.1	BD Biosciences	Cat#560493; RRID:AB_1645421
Anti-human T-bet; KIRAVIA Blue 520™; Isotype Mouse; Clone 4B10	BioLegend	Cat#644838; RRID:AB_2888710
Anti-human FOXP3; PE/Dazzle™ 594; Isotype Mouse; Clone 206D	BioLegend	Cat#320126; RRID:AB_2564024
Anti-human IL-22; Vio® B515; Isotype Human; Clone REA466	Miltenyi Biotec	Cat#130-108-096; RRID:AB_2652431
Anti-human GATA3; BV421; Isotype Mouse; Clone L50-823	BD Biosciences	Cat#563349; RRID:AB_2738152
CompBeads Anti-Mouse Ig, κ/Negative Control Compensation Particles Set	BD Biosciences	Cat#552843; RRID:AB_10051478
CompBeads Anti-Rat Ig, κ/Negative Control Compensation Particles Set	BD Biosciences	Cat#552844; RRID:AB_10055784
MACS® Comp Bead Kit, anti-REA	Miltenyi Biotec	Cat#130-104-693

**Biological samples**		

Primary Human CD4^+^ T cells isolated from buffy coats	Sanquin, Amsterdam, The Netherlands	N/A

**Chemicals, peptides, and recombinant proteins**		

1% paraformaldehyde	Apotheek LUMC	Cat#120810-001
Fetal Calf Serum	Bodinco BDC	Cat#16941
Dulbecco′s Modified Eagle′s Medium - high glucose	Sigma-Aldrich	Cat#D5796
Penicillin-Streptomycin	Lonza	Cat#DE17-602E
GlutaMAX™ Supplement	Thermo Fisher Scientific	Cat#35050038
Recombinant Human IL-2	PeproTech	Cat#200-02
CryoSure-Dimethyl Sulfoxide	WAK-Chemie Medical GmbH	Cat#WAK-DMSO-10
Oleic Acid	Sigma-Aldrich	Cat#O1383
HPLC Grade Ethanol	Thermo Fisher Scientific	Cat#64-17-5
Bovine Serum Albumin	Sigma-Aldrich	Cat#A7030
TaqMan™ Fast Advanced Master Mix	Thermo Fisher Scientific	Cat#4444557
Atorvastatin	Sigma-Aldrich	Cat#PHR1422
CP 640,186	Sanbio	Cat#17691-5
RPMI 1640, HEPES, no glutamine	Thermo Fisher Scientific	Cat#42401
Fetal Calf Serum	Serana	Cat#S-FBS-SA-015
Phorbol 12-myristate 13-acetate	Sigma-Aldrich	Cat#P8139
Ionomycin	Sigma-Aldrich	Cat#I0634
Brefeldin A	Sigma-Aldrich	Cat#B7651
LIVE/DEAD™ Fixable Blue Dead Cell Stain	Thermo Fisher Scientific	Cat#L34962
Bovine Serum Albumin Fraction V	Merck	Cat#10735086001
UltraPure™ 0.5M EDTA, pH 8.0	Thermo Fisher Scientific	Cat#15575020
Brilliant Stain Buffer	BD Biosciences	Cat#563794

**Critical commercial assays**		

*Quick*-DNA/RNA Microprep Plus Kit	Zymo Research	Cat#D7005
Qubit™ RNA, Broad Range (BR), Assay Kits	Thermo Fisher Scientific	Cat#Q10210
Agilent RNA 6000 Nano Kit	Agilent	Cat#5067-1511
Transcriptor First Strand cDNA Synthesis Kit	Roche	Cat#04897030001
Truseq Stranded mRNA Library Prep	Illumina	Cat#20020595
Ribo Zero Gold rRNA Depletion Kit	Illumina	Cat#20037135
eBioscience™ Foxp3 / Transcription Factor Staining Buffer Set	Thermo Fisher Scientific	Cat#00-5523-00

**Deposited data**		

Count data of RNA-sequencing	Gene Expression Omnibus repository, GEO	GSE231458

**Oligonucleotides**		

*CPT1A*	Thermo Fisher Scientific	Cat#4331182; Assay ID Hs00912671_m1
*RPL13A*	Thermo Fisher Scientific	Cat#4448892; Assay ID Hs03043887_gH
*SDHA*	Thermo Fisher Scientific	Cat#4453320; Assay ID Hs00188166_m1

**Software and algorithms**		

RStudio	RStudio, Inc.	v4.2.2
BD FACSDiva™ Software	BD Biosciences	v8.0.2
SpectroFlo® Software	Cytek Biosciences	v2.2.0.3
OMIQ	Dotmatics	N/A
BioRender	BioRender	N/A

### Resource availability

#### Lead contact

Further information and requests for resources and reagents should be directed to and will be fulfilled by the lead contact, Prof. dr. Bastiaan T. Heijmans (B.T.Heijmans@lumc.nl).

#### Materials availability

This study did not generate new unique reagents.

#### Data and code availability

• RNA sequencing data generated in this study have been deposited at Gene Expression Omnibus repository, accession, GEO, and are publicly available as of the date of publication. Accession numbers are listed in the key resources table.
• This paper does not report original code.
• Any additional information required to reanalyze the data reported in this paper is available from the lead contact upon request.

### Experimental model and study participant details

#### CD4^+^ T cell isolation and culture conditions

To obtain non-activated CD4^+^ T cells, peripheral blood mononuclear cells (PBMCs) were isolated from buffy coats of anonymous blood bank donors (Sanquin, Amsterdam, The Netherlands) by Ficoll paque (Apotheek LUMC, 97902861) gradient centrifugation. The sex of the cells could not be determined due to the anonymity of the donors. However, RNA sequencing showed that, of 9 donors sequenced, 8 were female and 1 was male, which was accounted for during the statistical analysis by correcting for donor effect. Next, CD4^+^ T cells were purified from the PBMCs using lyophilized human anti-CD4^+^ magnetically labeled microbeads (Miltenyi Biotec, 130-097-048) scaling the manufacturer’s instructions to ⅕ of the recommended volumes. CD4^+^ T cell authentication and purity was assessed on an LSR-II instrument at the Leiden University Medical Center Flow Cytometry Core Facility (https://www.lumc.nl/research/facilities/fcf/) with the BD FACSDiva™ v8.0.2 software (BD Biosciences). Cells were stained with anti-CD3-PE (BD Biosciences, 345765; RRID:AB_2868796), anti-CD4-APC (BD Biosciences, 345771; RRID:AB_2868799), anti-CD8-FITC (BD Biosciences, 555634; RRID:AB_395996), and anti-CD14-PEcy7 (BD Biosciences, 557742; RRID:AB_396848) and resuspended in 1% paraformaldehyde (Apotheek LUMC, 120810-001) to fix the cells prior to acquisition. Purity was >98% for all donors.

Prior to oleic acid exposure, ∼8∗10^7^ isolated cells were cultured overnight to allow the cells to return to a resting state after the stress of the isolation procedure. This was done in T75 flasks (Greiner Bio-One, 658-175) at a density of ∼2.5∗10^6^ cells/mL in 5% fetal calf serum (FCS) (Bodinco BDC, 16941) DMEM (Dulbecco’s Modified Eagle’s Serum (Sigma-Aldrich, D5796), 1% Pen-Strep (Lonza, DE17-602E), 1% GlutaMAX-1 (100x) (Thermo Fisher Scientific, 35050-038)) medium supplemented with 50 IU/mL IL-2 (PeproTech, 200-02) and incubated at 37°C under 5% CO_2_. To keep the cells in a non-activated state, no additional stimulus was added. Any CD4^+^ T cells not used directly after the isolation were kept in DMEM supplemented with 30% FCS, 1% Pen-Strep, 1% GlutaMAX-1, and 20% Dimethyl Sulfoxide (DMSO) (WAK-Chemie Medical GmbH, WAK-DMSO-10) medium, and stored in liquid nitrogen.

Next, non-activated CD4^+^ T cells were cultured with or without oleic acid for 0.5, 3, 24, 48, or 72 hours at 37°C under 5% CO_2_. To this end, CD4^+^ T cells from each donor were plated in a 24 wells plate (density of ∼4∗10^6^ cells/well) in 2mL 5% FCS DMEM for each time point, one exposed to oleic acid, one to the solvent control, and one to the negative control. Cells were cultured in medium containing FCS to ensure cell viability during culture and to be more comparable to physiological conditions of the circulation where other lipids are also present. Oleic acid (Sigma-Aldrich, O1383) was dissolved in HPLC grade ethanol (Thermo Fisher Scientific, 64-17-5) to a final concentration of 30,000μg/mL and complexed to fatty acid-free (FAF) bovine serum albumin (BSA) (Sigma-Aldrich, A7030) in a 2% FAF BSA DMEM mixture (Dulbecco’s Modified Eagle’s Serum, 2% FAF BSA, 1% Pen-Strep, 1% GlutaMAX-1 (100x)) to a final concentration of 150μg/mL. Complexing oleic acid mimics physiological conditions as fatty acids are also bound to albumin in the human circulation.^68^ Oleic acid was further diluted to the final concentrations of 10, 20, 30, and 50μg/mL. The concentrations tested were determined based on a literature search.^10^^,^^11^^,^^12^^,^^42^^,^^43^^,^^44^^,^^45^^,^^46^ For the solvent control samples, HPLC grade ethanol was diluted in 2% FAF BSA DMEM in the same volume as to dilute oleic acid to 150μg/mL and added to the wells. For the negative control samples, 2% FAF BSA DMEM was added directly to the wells with no additional solvent. The amount of 2% FAF BSA DMEM added to the wells was equal for each condition to keep the volumes equivalent. To assess the additional oleic acid stimulus to the non-activated CD4^+^ T cells due to FCS in the culture medium, an FCS sample was measured via the Shotgun Lipidomics Assistant (SLA) method^69^ to estimate the fraction of oleic acid in the sample. The sample was prepped as previously described^70^ but with two modifications, a starting volume of 25μL FCS and 600μL MTBE was added instead of 575μL during the first extraction. After exposure, the cells were flash frozen in liquid nitrogen and stored at -80°C until further use. Cell viability was measured via trypan blue staining (Sigma-Aldrich, T8154).

#### Spectral cytometry cell prep and activation

To study the effect of oleic acid pre-exposure on CD4^+^ T cell subset development, cells from 8 out of 9 donors that were previously analyzed using RNA-seq were thawed from liquid nitrogen; 1 donor could not be studied further because too few cells were available. Cells were cultured overnight to allow the cells to return to a resting state after the stress of the thawing, in T75 flasks at a density of ∼2.5∗10^6^ cells/mL in 5% FCS DMEM medium supplemented with 50 IU/mL IL-2 at 37°C under 5% CO_2_. To keep the cells in a non-activated state, no additional stimulus was added. Following overnight incubation, the cells were divided into 2 conditions, oleic acid and solvent exposed, and plated in a 24 wells plate (density of ∼4∗10^6^ cells/well) in 2mL 5% FCS DMEM. The oleic acid and solvent solution were prepared as stated previously, with one modification. To ensure that there was no effect of the solvent on T cell differentiation, the HPLC grade EtOH was evaporated before dissolving the oleic acid in 2% FAF BSA DMEM medium. The HPLC grade EtOH was also evaporated before adding the 2% FAF BSA DMEM medium in the solvent exposed condition, rendering it essentially the same as the negative control. These solutions were each added to the respective wells, where the final concentration of the oleic acid exposed conditions equaled 30μg/mL. The CD4^+^ T cells were cultured for 48h at 37°C under 5% CO_2_.

To ensure that the effect on CD4^+^ T cell differentiation was due to oleic acid pre-exposure, all medium of each condition was replaced by 5% FCS medium after 48h of exposure, before initiating the activation. Cell viability and diameter were first measured by Via1-Cassette™ (Chemometec, 941-0012) on a NucleoCounter® NC-200™ (Chemometec, 900-0200) and found to be > 90% for each condition. Then, 2 million cells were harvested by flash freezing in liquid nitrogen for *in vitro* model confirmation by RT-qPCR. The remaining cells were plated in a round bottom 96 wells plate (Corning Incorporated, 3799), at a density of 100,000 cells/well, and were activated for 72h using Dynabeads™ Human T-Activator CD3/CD28 for T Cell Expansion and Activation (Thermo Fisher Scientific, 11161D; RRID:AB_2916088) according to the manufacturer’s instructions at 37°C under 5% CO_2_. Half the cells from each exposure were activated and the other half was left in the non-activated state. Subsequently, the cells from each pre-exposure and activation state were pooled in Eppendorf tubes and the beads were magnetically removed from the activated cells. Cell viability and diameter were measured by Via1-Cassette™ after 72h. Cells were then used for T cell subset identification described in more detail below. All centrifugation steps were performed at 1500 rpm at room temperature.

#### Inhibitor culture conditions and activation

To study whether the effect of oleic acid pre-exposure on CD4^+^ T cell subset development could be prevented by metabolic inhibitors, cells from 3 out of 8 donors that were previously analyzed for subset development were thawed from liquid nitrogen; 5 donors could not be studied further because too few cells were available. Cells were cultured overnight to allow the cells to return to a resting state after the stress of the thawing, in T75 flasks at a density of ∼2.5∗10^6^ cells/mL in 5% FCS DMEM medium supplemented with 50 IU/mL IL-2 at 37°C under 5% CO_2_. To keep the cells in a non-activated state, no additional stimulus was added. Following overnight incubation, the cells were divided into 5 conditions, solvent, oleic acid, oleic acid + atorvastatin (Sigma-Aldrich, PHR1422), oleic acid + CP-640186 (Sanbio, 17691-5), and oleic acid + atorvastatin + CP-640186 exposed, and plated in a 24 wells plate (density of ∼4∗10^6^ cells/well) in 2mL 5% FCS DMEM. The oleic acid and solvent solution were prepared as stated previously, with HPLC grade EtOH evaporation. These solutions were each added to the respective wells, where the final concentration of the oleic acid exposed conditions equaled 30μg/mL. Atorvastatin and CP-640186 were added to the respective wells at a concentration of 10μM and 20μM, respectively. The CD4^+^ T cells were cultured for 48h at 37°C under 5% CO_2_.

To ensure that the effect on CD4^+^ T cell differentiation was due to oleic acid and inhibitor pre-exposure, all medium of each condition was replaced by 5% FCS medium after 48h of exposure, before initiating the activation. Cell viability and diameter were first measured by Via1-Cassette™ on a NucleoCounter® NC-200™ and found to be > 90% for each condition. Then, ∼0.5-1.5 million cells were harvested by flash freezing in liquid nitrogen for *in vitro* model confirmation by RT-qPCR. The remaining cells were plated in a round bottom 96 wells plate, at a density of 100,000 cells/well, and were activated for 72h using Dynabeads™ Human T-Activator CD3/CD28 for T Cell Expansion and Activation according to the manufacturer’s instructions at 37°C under 5% CO_2_. Subsequently, the cells from each pre-exposure were pooled in Eppendorf tubes and the beads were magnetically removed. Cell viability and diameter were measured by Via1-Cassette™ after 72h. Cells were then used for T cell subset identification described in more detail below. All centrifugation steps were performed at 1500 rpm at room temperature.

### Method details

#### RNA isolation

To isolate total RNA for RNA sequencing and RT-qPCR, RNA was extracted from the cell samples using the Zymo Quick-DNA/RNA Microprep Plus Kit (Zymo Research, D7005) according to manufacturer’s instructions. The RNA was quantified using a Qubit® 2.0 Fluorometer (Q32866) with the Qubit® RNA BR Assay Kit (Thermo Fisher Scientific, Q10211) according to manufacturer’s instructions. RNA integrity (RIN) values of the samples were on average 8.40 SE 0.14 as determined using an Agilent 2100 Bioanalyzer Instrument (G2939BA) with the Agilent RNA 6000 Nano Reagents (5067-1511). RNA was divided into two samples and stored at -80°C, 1μg for RNA sequencing and the rest for cDNA synthesis and RT-qPCR measurements.

#### Real time-Quantitative PCR

To measure the expression of *CPT1A* in all the cell samples, cDNA was synthesized with 200ng of the stored RNA using the Transcriptor First Strand cDNA Synthesis Kit (Roche, 04897030001) according to the manufacturer’s instructions. Quantitative real time PCR’s for *CPT1A* (Thermo Fisher Scientific, 4331182; Assay ID: Hs00912671_m1) were performed using the TaqMan™ Fast Advanced Master Mix (Thermo Fisher Scientific, 4444557) with 10ng cDNA per reaction on a QuantStudio 6 Real-Time PCR system (Applied Biosystems). All RT-qPCR reactions were performed in triplicate and outliers were removed if the Ct value measured differed more than 0.5% from the mean. Relative gene expression levels (-ΔCt) were calculated using the average of Ct values of *RPL13A* (Thermo Fisher Scientific, 4448892; Assay ID: Hs03043887_gH) and *SDHA* (Thermo Fisher Scientific, 4453320; Assay ID: Hs00188166_m1) as internal controls.^71^ The fold change was determined using the 2^-ΔΔCt^ method, using the negative control as the reference. All statistical analyses were performed in R. Data are expressed as mean of the relative fold change and standard error. The reported P values were determined by applying a paired two-tailed student’s T test. P values < 0.05 were considered to be statistically significant.

#### RNA sequencing

RNA sequencing (RNA-seq) was performed to determine the differences in the transcriptome of oleic acid versus solvent exposed non-activated CD4^+^ T cells across time. 1μg of total RNA from each of the samples was sent for sequencing (Macrogen, Amsterdam, NL), each with a concentration above 20ng/μL in 50μL solution. RNA-seq libraries were prepared from 200ng RNA using the Illumina Truseq stranded mRNA library prep (Illumina, 20020595) after depletion of ribosomal RNA with Ribo Zero Gold (Illumina, 20037135). Both whole-transcriptome amplification and sequencing library preparations were performed in two 96-well plates with half the samples each, to reduce assay-to-assay variability. Quality control steps were included to determine total RNA quality and quantity, the optimal number of PCR preamplification cycles, and fragment size selection. No samples were eliminated from further downstream steps. Barcoded libraries were pooled and equally divided across two lanes to ensure an equal distribution of all the samples across the two lanes. Barcoded libraries were sequenced to a read depth of 30 million reads using the Novaseq 6000 (Illumina) to generate 100 base pair paired-end reads.

#### Spectral cytometry

Prior to FACS analysis, cells were washed in RPMI 1640 medium (Thermo Fisher Scientific, 42401), supplemented with 100U/mL penicillin, 100μg/mL streptomycin, 1mM pyruvate, 2mM glutamate, and 10% FCS (Serana, S-FBS-SA-015), and adjusted to a concentration of 1x10^6^ cells/mL. Cells were then resuspended in 100μL RPMI + 10% FCS and stimulated for 4h with Phorbol 12-myristate 13-acetate (PMA; 100ng/mL, Sigma-Aldrich, P8139) and ionomycin (1μg/mL, Sigma-Aldrich, I0634) at 37°C under 5% CO_2_ to promote cytokine production.^72^ After 2h of stimulation, 10μg/mL of the protein transport inhibitor Brefeldin A (Sigma-Aldrich, B7651) was added.

After stimulation, the cells were washed twice in phosphate-buffered saline (PBS), stained for viable cells with LIVE/DEAD™ Fixable Blue (Thermo Fisher Scientific, L34962) for 30min at room temperature, then washed twice in fluorescence-activated cell sorting (FACS) buffer (PBS supplemented with 0.5% BSA (Merck, 10735086001) and 2mM EDTA (Thermo Fisher Scientific, 15575020)). The antibody surface cocktail consisted of 11 markers, anti-CD38-APC-Fire810 (BioLegend, 356643; RRID:AB_2860936), anti-CD8-Pacific Orange (Thermo Fisher Scientific, MHCD0830; RRID:AB_10372066), anti-CD25-BUV563 (BD Biosciences, 612918; RRID:AB_2870203), anti-CD45RA-BUV496 (BD Biosciences, 741182; RRID:AB_2870749), anti-CD45RO-BUV805 (BD Biosciences, 748367; RRID:AB_2872786), anti-CD4-cFluor® YG584 (Cytek Biosciences, SKU R7-20041), anti-CD3-BUV395 (BD Biosciences, 563546; RRID:AB_2744387), anti-CD27-APC-H7 (BD Biosciences, 560222; RRID:AB_1645474), anti-PD1-BV750 (BD Biosciences, 747446; RRID:AB_2872125), anti-CD127-R718 (BD Biosciences, 566967; RRID:AB_2869977), and anti-CCR7-BV785 (BioLegend, 353230; RRID:AB_2561371). For the spectral cytometry of the inhibitor experiment, the same surface cocktail was used except for the CD8 marker. The antibody surface cocktail was prepared in FACS buffer containing 20% Brilliant Stain Buffer Plus (BD Biosciences, 563794) was added to the cells and incubated for 30min at room temperature. Cells were then washed twice in FACS buffer and afterwards fixed and permeabilized with the Fixation/Permeabilization solution from the eBioscience™ FoxP3 / Transcription Factor Staining Buffer Set (Thermo Fisher Scientific, 00-5523-00) according to the manufacturer’s instructions for 30min at 4°C. Subsequently, cells were washed twice with the Permeabilization buffer from the eBioscience™ FoxP3 / Transcription Factor Staining Buffer Set before being stained with the intracellular/intranuclear antibody cocktail for 30min at 4°C. The intracellular/intranuclear antibody cocktail consisted of 14 markers, anti-TNF-PE-Cy7 (BD Biosciences, 557647; RRID:AB_396764), anti-IL-17A-Pacific Blue (BioLegend, 512312; RRID:AB_961392), anti-IL-5-APC (BioLegend, 504306; RRID:AB_315329), anti-IL-4-BUV737 (BD Biosciences, 612835; RRID:AB_2870157), anti-IFN-γ-BV650 (BD Biosciences, 563416; RRID:AB_2738193), anti-IL-13-BV711 (BD Biosciences, 564288; RRID:AB_2738731), anti-IL-9-PE (BioLegend, 507605; RRID:AB_315487), anti-IL-10-PerCP-eFluor™ 710 (Thermo Fisher Scientific, 46-7108-42; RRID:AB_2573833), anti-CD152-PE-Cy5 (BD Biosciences, 555854; RRID:AB_396177), anti-IL-21-Alexa Fluor® 647 (BD Biosciences, 560493; RRID:AB_1645421), anti-T-bet-KIRAVIA Blue 520™ (BioLegend, 644838; RRID:AB_2888710), anti-FOXP3-PE/Dazzle™ 594 (BioLegend, 320126; RRID:AB_2564024), anti-IL-22-Vio® B515 (Miltenyi Biotec, 130-108-096; RRID:AB_2652431), and anti-GATA3-BV421 (BD Biosciences, 563349; RRID:AB_2738152). For the spectral cytometry of the inhibitor experiment, the same intracellular/intranuclear antibody cocktail was used except for the IL-5 marker. Lastly, cells were washed with eBioscience™ Permeabilization buffer followed by another wash in FACS buffer. All centrifugation steps before fixation were performed at 300x g at room temperature and after fixation at 800x g at 4°C. Single-stain reference controls were either cells or UltraComp eBeads™ (CompBeads Anti-Mouse Ig, κ/Negative Control Compensation Particles Set (BD Biosciences; 552843; RRID:AB_10051478); CompBeads Anti-Rat Ig, κ/Negative Control Compensation Particles Set (BD Biosciences; 552844; RRID:AB_10055784), or MACS® Comp Bead Kit, anti-REA (Miltenyi Biotec; 130-104-693)). Cells were used as unstained reference control. All reference controls underwent the same protocol as the fully stained samples, including washes, buffers used, and fixation and permeabilization steps.

For acquisition, cells were resuspended in FACS buffer and acquired on a 5L-Cytek Aurora instrument at the Leiden University Medical Center Flow Cytometry Core Facility with the SpectroFlo® v2.2.0.3 software (Cytek Biosciences). Data was manually gated in OMIQ (Dotmatics, 2023). All statistical analyses were performed in R. Data are expressed as mean of the relative fold change and standard error. The reported P values were determined by applying a paired two-tailed student’s T test. Differences with P_FDR_ < 0.05 (Benjamini-Hochberg) were considered to be significant.

### Quantification and statistical analysis

#### Statistical analyses

All statistical analyses were performed in R (v4.2.2). Statistical details per experiment can be found in the “Method details” section of the “STAR Methods” as well as in the (supplemental) figure and table legends. A detailed description of the methods used to analyze the RNA sequencing data can be found below in the section “RNA Sequencing Analysis”. For the analysis of all other experiments, the results are presented as mean ± SEM values. The reported P values were determined by applying a paired two-tailed student’s T test between control and oleic acid exposed samples. Differences with P_FDR_ < 0.05 (Benjamini-Hochberg) were considered to be significant.

#### RNA sequencing analysis

RNA-seq reads were processed using the BioWDL RNAseq pipeline (v3.0.0) developed at LUMC (http://zenodo.org/record/3713261#.ZF98HdJBw5k). Quality controls were performed using FastQC (v0.11.7) and MultiQC (v1.7). Cleaned reads were aligned to the human reference genome GRCh38 using STAR aligner (v2.7.3a). Gene count table was generated using Htseq-count (v0.11.2) with Ensembl gene annotation version 99. Based on Ensembl gene biotype annotation, we included only protein coding genes for further downstream analysis (19,916 genes in total). We used the Bioconductor package *DESeq2*^73^ (v1.40.1) to test whether oleic acid had an effect on gene expression at any time point. *DESeq2* fits a generalized linear model (GLM) assuming the negative binomial distribution for the counts. The model expresses the logarithm of the average of the counts in terms of one of more predictors. In this case, we compared two models: The first “null” model has only timepoint (as a categorical variable with 5 levels) and subject identifier as predictors. By including the subject identifier in the model, we account for the dependence between measurements within the same subject. The second “alternative” model also includes the interaction between phenotype (oleic acid as a numerical measurement) and timepoint. We compare the fit of the two models with a likelihood ratio test. As part of the *DESeq2* process lowly expressed genes were automatically removed, resulting in 12,932 analyzed genes.^73^ The Benjamini-Hochberg procedure was used to correct for multiple testing and a false discovery rate (FDR) < 0.05 was considered statistically significant.

Next, to identify distinct gene expression patterns in the data, unsupervised K means clustering was performed on the differentially expressed genes using the *factoextra*^74^ package (v1.0.7). The number of clusters, *k*, was chosen using the elbow, silhouette, and gap-statistic method. Heatmaps were constructed using *ComplexHeatmap*^75^ (v2.14.0) by plotting the log2FoldChange of the DEGs at each time point.

The identified clusters were then mapped for pathway enrichment. 10 human pathway databases (BioPlanet 2019, WikiPathways 2019 Human, KEGG 2019 Human, Elsevier Pathway Collection, BioCarta 2015, Reactome 2016, HumanCyc 2016, NCI-Nature 2016, Panther 2016 and MSigDB Hallmark 2020) were queried using gene symbols, with 430 of 544 queried genes present in at least 1 database. The identified clusters were then mapped for pathway enrichment using *clusterProfiler*^76^ (v4.6.2) with the background set to 12,932 expressed genes in the CD4^+^ T cells based on *DESeq2* filtering. Multiple testing using the Benjamini-Hochberg method at 5% FDR was performed over the combined results from the 10 databases. Pathways that included highly similar gene sets were grouped (Jaccard index > 0.7) and only the most significantly enriched pathway per group was retained. Furthermore, using the UniProt IDs of the enriched genes, the Path-MAP function of the PathBank database^77^ was used to visualize the list of matching components within specific canonical pathways. *De novo* motif analysis on promotors of differentially regulated genes was performed using HOMER.^78^

## Supplemental information

Document S1. Figures S1–S17

Table S1. Changes in gene expression and CD4^+^ T cell markers due to oleic acid pre-exposure, related to Figures 1, 2, 3, and 4 (a) Upregulated differentially expressed genes in order of significance along with their Ensembl ID, Gene Name, Enzyme Name, UniProt ID, chromosome location, start site, end site, base mean, log2 fold change, log2 fold change at 0.5h, log2 fold change at 3h, log2 fold change at 24h, log2 fold change at 48h, log2 fold change at 72h, standard error, test statistic, p value, and adjusted p value. The log2FoldChange at specific time points were used to create the heatmap, and the UniProt ID was used for Path-MAP in PathBank. (b) Downregulated differentially expressed genes in order of significance along with their Ensembl ID, Gene Name, Enzyme Name, UniProt ID, chromosome location, start site, end site, base mean, log2 fold change, log2 fold change at 0.5h, log2 fold change at 3h, log2 fold change at 24h, log2 fold change at 48h, log2 fold change at 72h, standard error, test statistic, p value, and adjusted p value. The log2FoldChange at specific time points were used to create the heatmap, and the UniProt ID was used for Path-MAP in PathBank. (c) Transcription factors, extracellular markers, and intracellular markers measured in non-activated CD4^+^ T cell after pre-exposure to oleic acid. Values are shown as a percent of the parent, n = 8. (d) Transcription factors, extracellular markers, and intracellular markers measured in activated CD4^+^ T cell after pre-exposure to oleic acid. The concentration of oleic acid was 30 μg/mL. Values are shown as a percent of the parent, n = 8. (e) Coexpression analysis of various CD4^+^ T cell markers in activated CD4^+^ T cells after pre-exposure to oleic acid. The concentration of oleic acid was 30 μg/mL. Values are shown as a percent of the parent, n = 8. (f) Transcription factors, extracellular markers, and intracellular markers measured in non-activated CD4^+^ T cell after pre-exposure to oleic acid. Values are shown as a percent of the parent, n = 8. In order to calculate the fold change of CD127^low^CD25^hi^FoxP3^+^ for D10, D11 and D12 add a "1" to each value for that donor. (g) Transcription factors, extracellular markers, and intracellular markers measured in activated CD4^+^ T cell after pre-exposure to oleic acid. The concentration of oleic acid was 28 μg/mL. Values are shown as a percent of the parent, n = 8. In order to calculate the fold change of CD127^low^CD25^hi^FoxP3^+^ for D11 and D12 add a "1" to each value for that donor. (h) Intracellular markers measured in activated CD4^+^ T cell after pre-exposure either oleic acid, oleic acid + atorvastatin, oleic acid + CP-640186, or oleic acid + atorvastatin + CP-640186. The concentration of oleic acid was 30 μg/mL, that of atorvastatin was 10 μM, and that of CP-640186 was 20 μM. The donor numbers are labeled respectively of the donors used in the previous experiments. Values are shown as a percent of the parent, n = 3.
