# Supplementary material for: Oleic acid triggers metabolic rewiring of T cells poising them for T helper 9 differentiation
Source: iScience. 2024 Mar 12;27(4):109496. doi: 10.1016/j.isci.2024.109496 (PMC10981094; doi:10.1016/j.isci.2024.109496)
Supplement: Document S1. Figures S1–S17 [file mmc1.pdf]

iScience, Volume *27*

Supplemental information

Oleic acid triggers metabolic rewiring of T cells poising them for T helper 9 differentiation

Nathalie A. Reilly, Friederike Sonnet, Koen F. Dekkers, Joanneke C. Kwekkeboom, Lucy Sinke, Stan Hilt, Hayat M. Suleiman, Marten A. Hoeksema, Hailiang Mei, Erik W. van Zwet, Bart Everts, Andreea Ioan-Facsinay, J. Wouter Jukema, and Bastiaan T. Heijmans

a

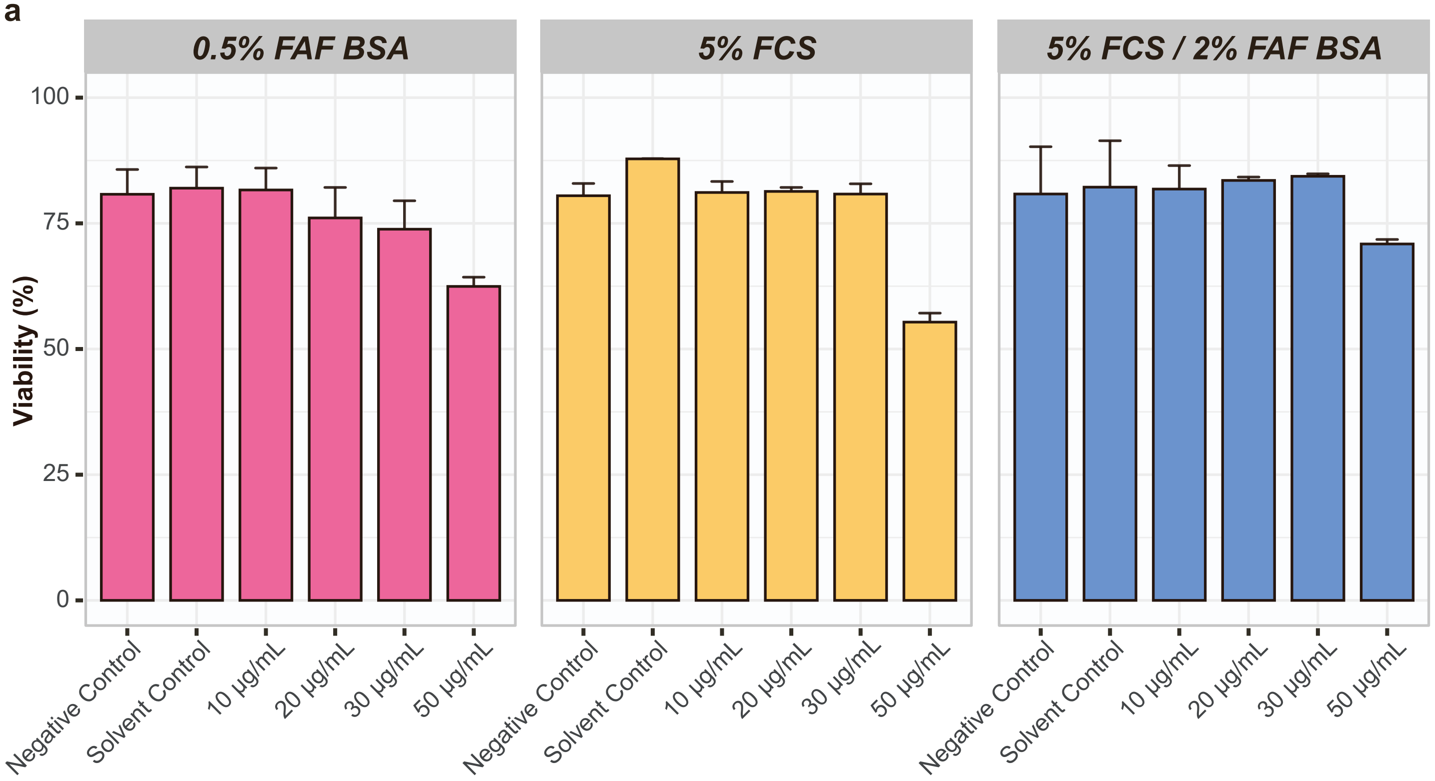
0.5% FAF BSA
5% FCS
5% FCS / 2% FAF BSA
Viability (%)
100
75
50
25
0
Negative Control
Solvent Control
10 µg/mL
20 µg/mL
30 µg/mL
50 µg/mL

b

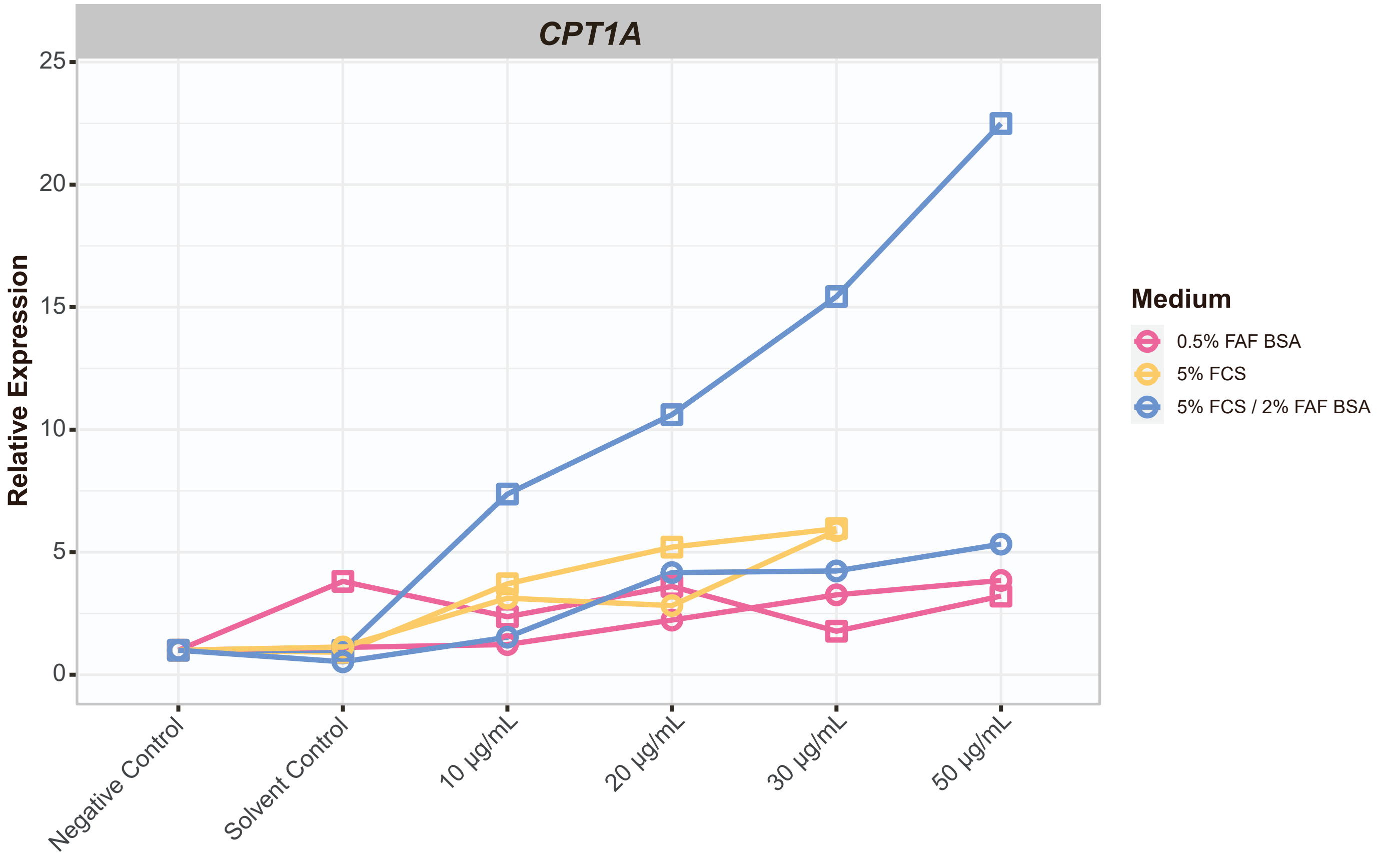
CPT1A
Relative Expression
25
20
15
10
5
0
Negative Control
Solvent Control
10 µg/mL
20 µg/mL
30 µg/mL
50 µg/mL
Medium
0.5% FAF BSA
5% FCS
5% FCS / 2% FAF BSA

Supplemental Figure 1: Determination of culture medium type and concentration of oleic acid to use in the *in vitro* model by viability and *CPT1A* expression. Related to Figure 1. Three different medium types were tested. First, cells cultured in and oleic acid dissolved in FAF BSA only. Second, cells cultured in and oleic acid dissolved in 5% FCS only. Third, cells cultured in 5% FCS and oleic acid dissolved in FAF BSA. Non-activated CD4$^+$ T cells were exposed to 10, 20, 30, or 50µg/mL oleic acid for 48h. Conditions are labeled by color. The greatest upregulation while maintaining cell viability occurred at 30µg/mL oleic acid in the 5% FCS / 2% FAF BSA medium combination, n = 2. **(a)** Bar plot showing the average cell viability and standard error in percent, as determined by trypan blue staining, for 2 donors for each medium and concentration tested after 48h exposure. For FAF BSA medium only, the average viability was 80.79 SE 4.93% at negative control, 82.00 SE 4.22% at solvent control, 81.64 SE 4.37% at 10µg/mL, 76.07 SE 6.07% at 20µg/mL, 73.84 SE 5.66% at 30µg/mL, and 62.45 SE 1.84% at 50µg/mL. For 5% FCS medium only, the average viability was 80.49 SE 2.44% at negative control, 87.82 SE 0.06% at solvent control, 81.14 SE 2.19% at 10µg/mL, 81.35 SE 0.79% at 20µg/mL, 80.83 SE 2.04% at 30µg/mL, and 55.36 SE 1.79% at 50µg/mL. For the combination of 5% FCS and FAF BSA, the average cell viability was 80.84 SE 9.41% at negative control, 82.20 SE 9.23% at solvent control, 81.82 SE 43.68% at 10µg/mL, 83.54 SE 0.68% at 20µg/mL, 84.36 SE 0.49% at 30µg/mL, and 70.90 SE 0.90% at 50µg/mL. The solvent control had no effect on CD4$^+$ T cell viability as expected. Oleic acid had no effect on CD4$^+$ T cell viability until 50µg/mL. **(b)** Line plot showing the relative expression of *CPT1A*, as determined by RT-qPCR, per donor for each medium and concentration tested after 48h exposure. Data is shown relative to the negative control condition. As expected, the solvent was not found to have any effect on *CPT1A* expression, in any of the medium types tested (3.81 fold for FAF BSA only, 1.02 SE 0.11 fold for 5% FCS only, and 0.76 SE 0.23 fold for the combination of 5% FCS and FAF BSA). No RNA was extracted from the second donor in the solvent control condition making the mean only the mean of the first donor and therefore also no SE could be calculated. For FAF BSA medium only, on average, oleic acid exposure caused *CPT1A* to be upregulated 1.80 SE 0.57 fold at 10µg/mL, 2.92 SE 0.69 fold at 20µg/mL, and 2.52 SE 0.74 fold at 30µg/mL, and 3.53 SE 0.32 fold at 50µg/mL. *CPT1A* expression increased inconsistently, most likely due to insufficient nutrients (often supplied by FCS) for the cells to survive and behave as they normally would. For 5% FCS medium only, on average, oleic acid exposure caused *CPT1A* to be upregulated 3.42 SE 0.29 fold at 10µg/mL, 4.01 SE 1.19 fold at 20µg/mL, and 5.92 SE 0.04 fold at 30µg/mL. Oleic acid exposure increased the expression of *CPT1A* gradually with increasing concentrations until 50µg/mL where the lack of albumin bound oleic acid became toxic and the cells died, making it impossible to extract sufficient quality RNA for RT-qPCR analysis. For the combination of 5% FCS and FAF BSA, on average, oleic acid exposure caused *CPT1A* to be upregulated 4.45 SE 2.92 fold at 10µg/mL, 7.39 SE 3.22 fold at 20µg/mL, 9.83 SE 5.60 fold at 30µg/mL, and 13.91 SE 8.58 fold at 50µg/mL. Oleic acid exposure increased the expression of *CPT1A* gradually with increasing concentrations.

a

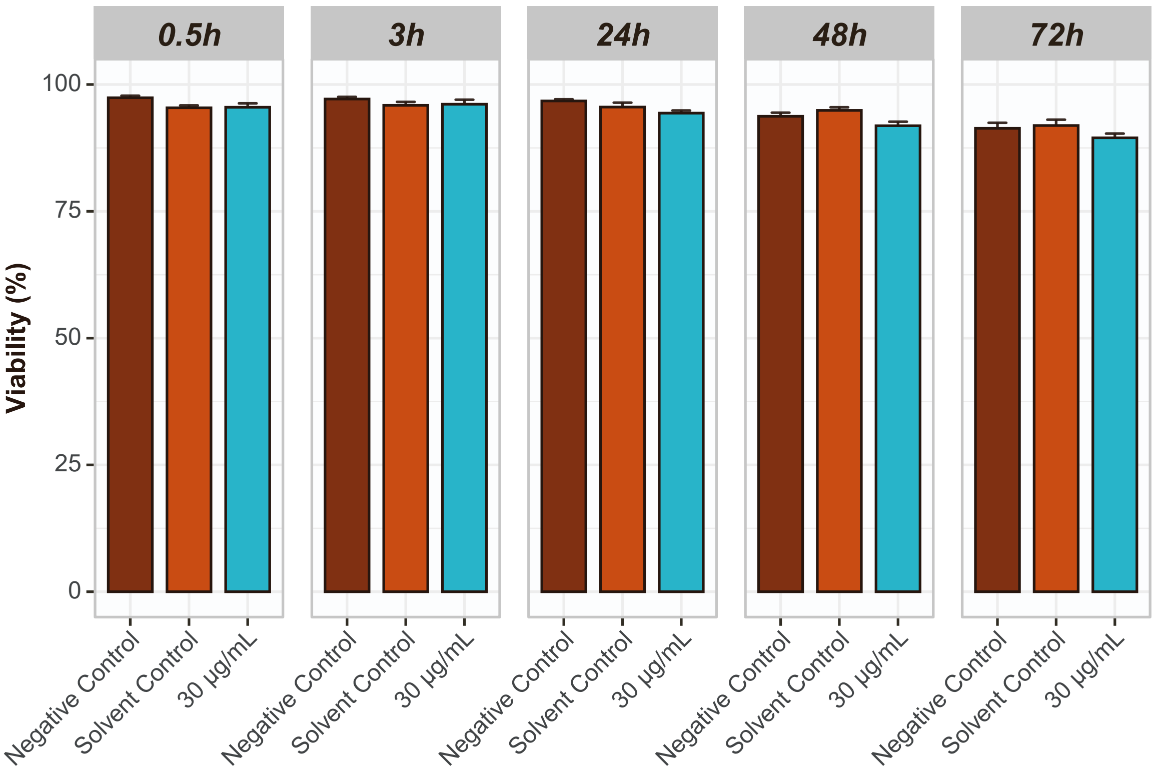
0.5h
3h
24h
48h
72h
Viability (%)
100
75
50
25
0
Negative Control
Solvent Control
30 µg/mL

b

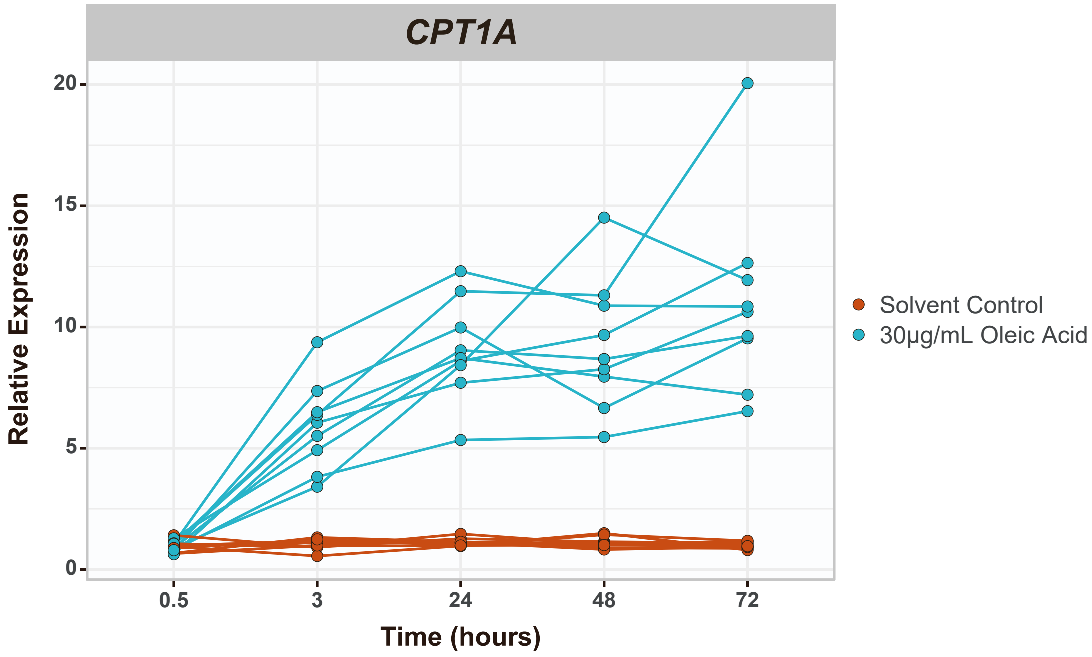
CPT1A
Relative Expression
20
15
10
5
0
0.5
3
24
48
72
Time (hours)
Solvent Control
30µg/mL Oleic Acid

Supplemental Figure 2: Verification of *in vitro* model prior to RNA sequencing by viability and *CPT1A* expression. Related to Figure 1. **(a)** Bar plot showing the average cell viability and standard error in percent, as determined by trypan blue exclusion. On average the cell viability of the negative control exposed cells was 97.4 SE 0.4% at 0.5h, 97.1 SE 0.4% at 3h, 96.8 SE 0.3% at 24h, 93.7 SE 0.7% at 48h, and 91.4 SE 1.1% at 72h. On average the cell viability of the solvent control exposed cells was 95.4 SE 0.5% at 0.5h, 95.9 SE 0.7% at 3h, 95.6 SE 0.9% at 24h, 94.9 SE 0.6% at 48h, and 91.9 SE 1.2% at 72h. On average the cell viability of the oleic acid exposed cells was 95.5 SE 0.8% at 0.5h, 96.1 SE 0.9% at 3h, 94.4 SE 0.5% at 24h, 91.8 SE 0.8% at 48h, and 89.4 SE 0.8% at 72h. Thus, neither the controls nor the exposure had an effect on $CD4^+$ T cell viability, as expected, n = 9. **(b)** Line plot showing the relative expression of *CPT1A* per donor across time by RT-qPCR confirming the effect of oleic acid on $CD4^+$ T cells in the *in vitro* model and the absence of an effect of solvent. Values are colored by exposure across time. In solvent control exposed samples, there was no effect on *CPT1A* expression with a relative expression of 1.0 SE 0.07 fold at 0.5h, 1.0 SE 0.08 fold at 3h, 1.1 SE 0.05 fold at 24h, 1.1 SE 0.08 fold at 48h, and 1.0 SE 0.04 fold at 72h as compared to the negative control. In oleic acid exposed samples, on average *CPT1A* was upregulated 0.9 SE 0.07 fold at 0.5h, 5.9 SE 0.61 fold at 3h, 9.1 SE 0.68 fold at 24h, 9.3 SE 0.90 fold at 48h, and 11.0 SE 1.30 fold at 72h as compared to the negative control. The solvent control has no effect gene expression and can therefore be used as a comparison for the differential gene expression analysis, n = 9.

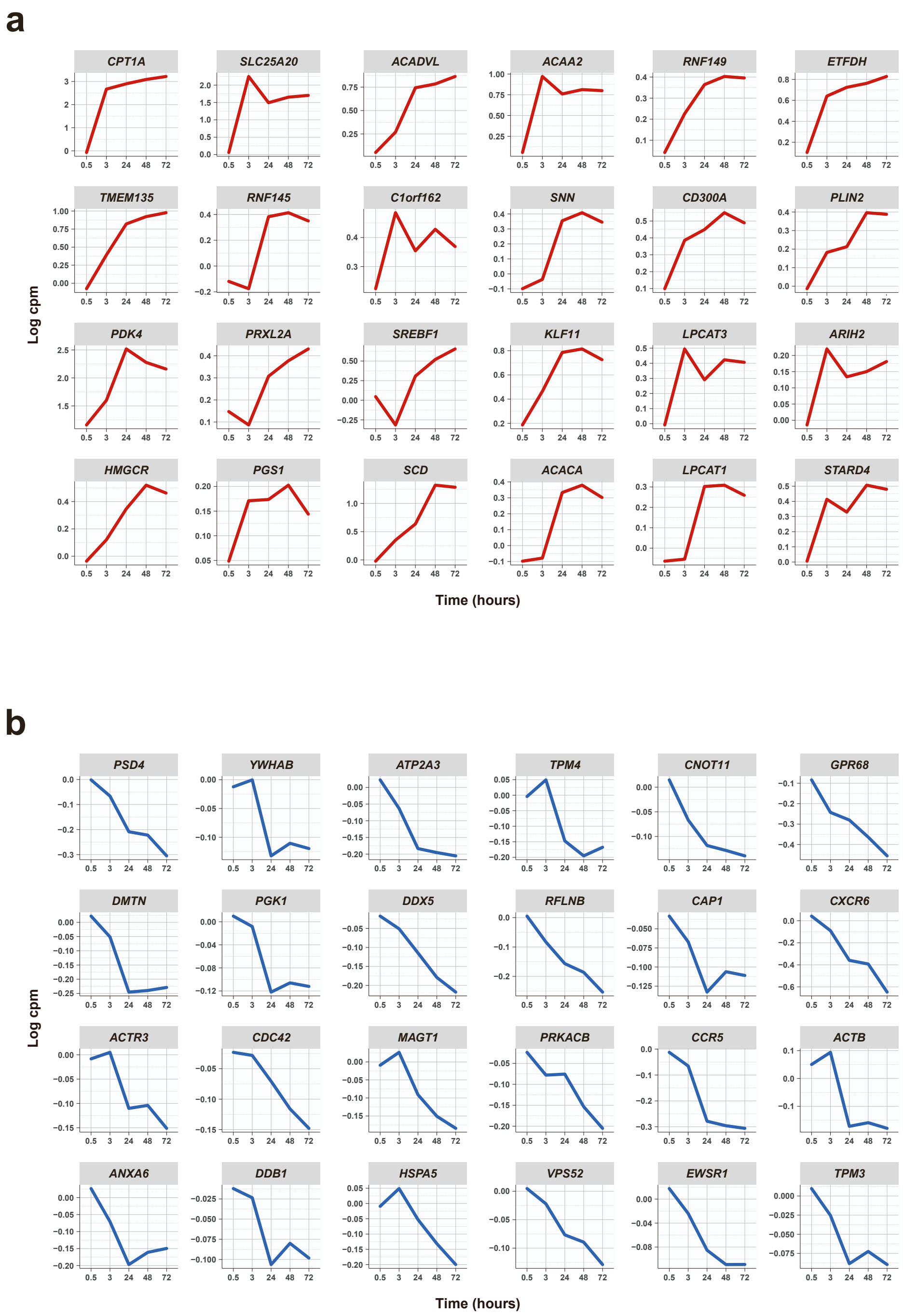

a
CPT1A
SLC25A20
ACADVL
ACAA2
RNF149
ETFDH
TMEM135
RNF145
C1orf162
SNN
CD300A
PLIN2
PDK4
PRXL2A
SREBF1
KLF11
LPCAT3
ARIH2
HMGCR
PGS1
SCD
ACACA
LPCAT1
STARD4
Log cpm
Time (hours)
b
PSD4
YWHAB
ATP2A3
TPM4
CNOT11
GPR68
DMTN
PGK1
DDX5
RFLNB
CAP1
CXCR6
ACTR3
CDC42
MAGT1
PRKACB
CCR5
ACTB
ANXA6
DDB1
HSPA5
VPS52
EWSR1
TPM3
Log cpm
Time (hours)

Supplemental Figure 3: Top differentially expressed genes from cluster 1 and 2. Related to Figure 1 **(a)** Cluster 1 differentially expressed genes. Line plots showing mean expression values (read counts) of indicated genes from cluster 1 across time analyzed by RNA-Seq. **(b)** Cluster 2 differentially expressed genes. Line plots showing mean expression values (read counts) of indicated genes from cluster 2 across time analyzed by RNA-Seq.

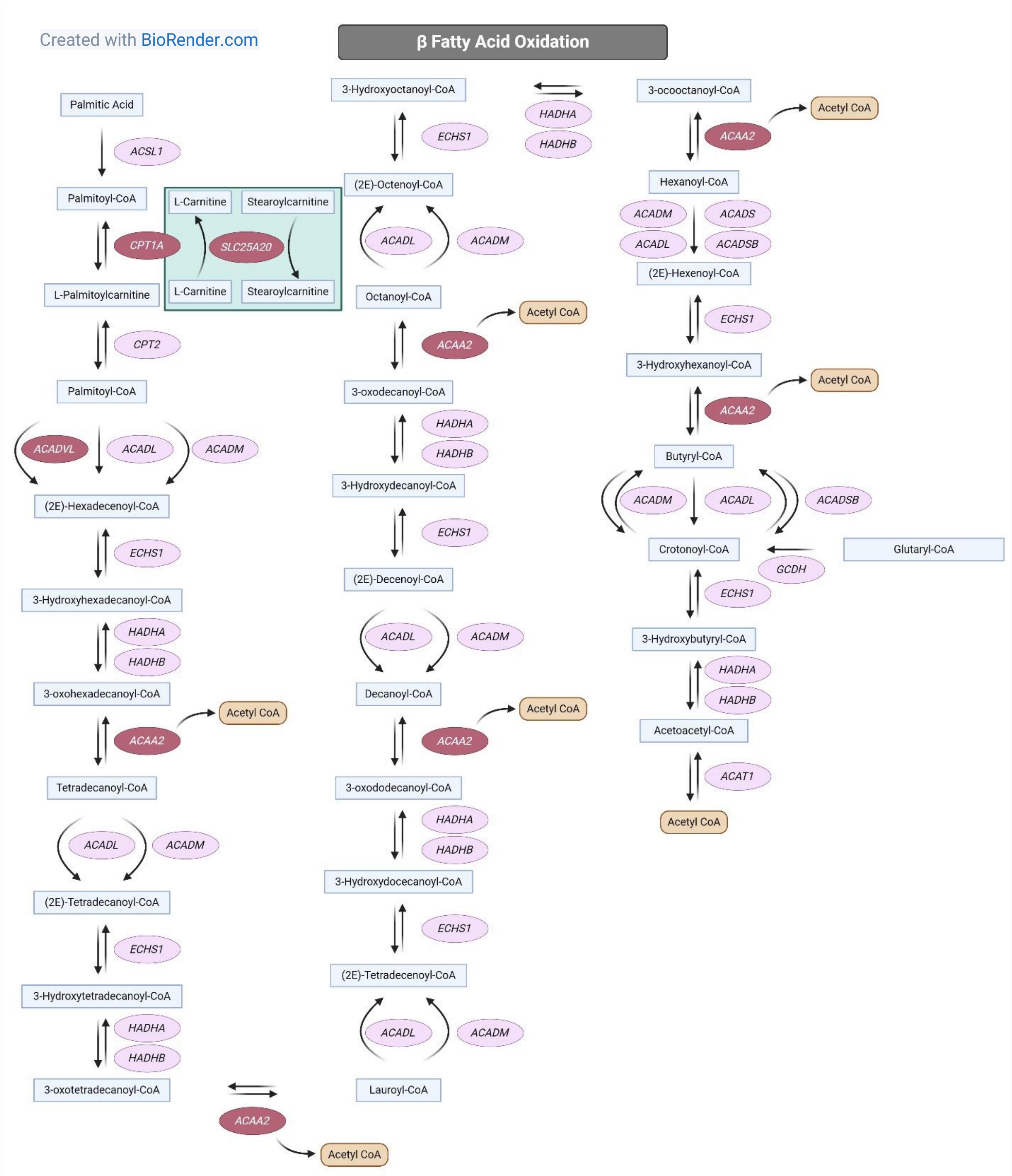

Supplemental Figure 4: Visualization of the Path-MAP identified overlap of differentially expressed genes within the β fatty acid oxidation pathway. Related to Figure 2. Overall, a total of 4 out of 16 enzymes involved in the β fatty acid oxidation were upregulated in our oleic acid exposed non-activated CD4$^+$ T cells. Compounds are in blue boxes, enzymes not differentially expressed in the RNA sequencing data are in light pink ovals, enzymes present in the RNA sequencing data are in red ovals, the arrows indicate the direction of movement of the process. Visualization created in BioRender.com.

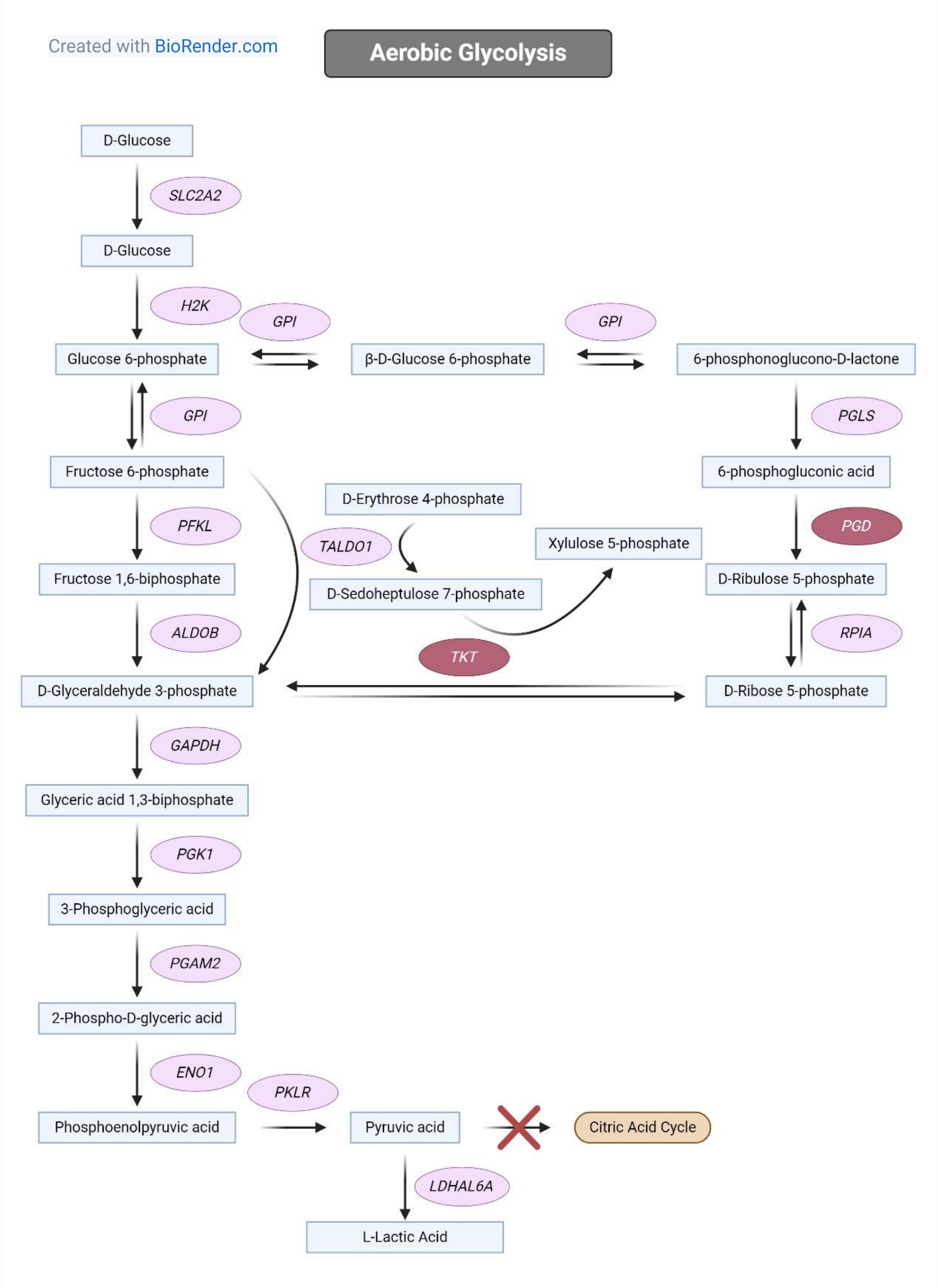

Supplemental Figure 5: Visualization of the Path-MAP identified overlap of differentially expressed genes within the aerobic glycolysis pathway. Related to Figure 2. Overall, a total of 2 out of 15 enzymes involved in aerobic glycolysis were upregulated in our oleic acid exposed non-activated CD4^{+} T cells. Compounds are in blue boxes, enzymes not differentially expressed in the RNA sequencing data are in light pink ovals, enzymes present in the RNA sequencing data are in red ovals, the arrows indicate the direction of movement of the process. Visualization created in BioRender.com.

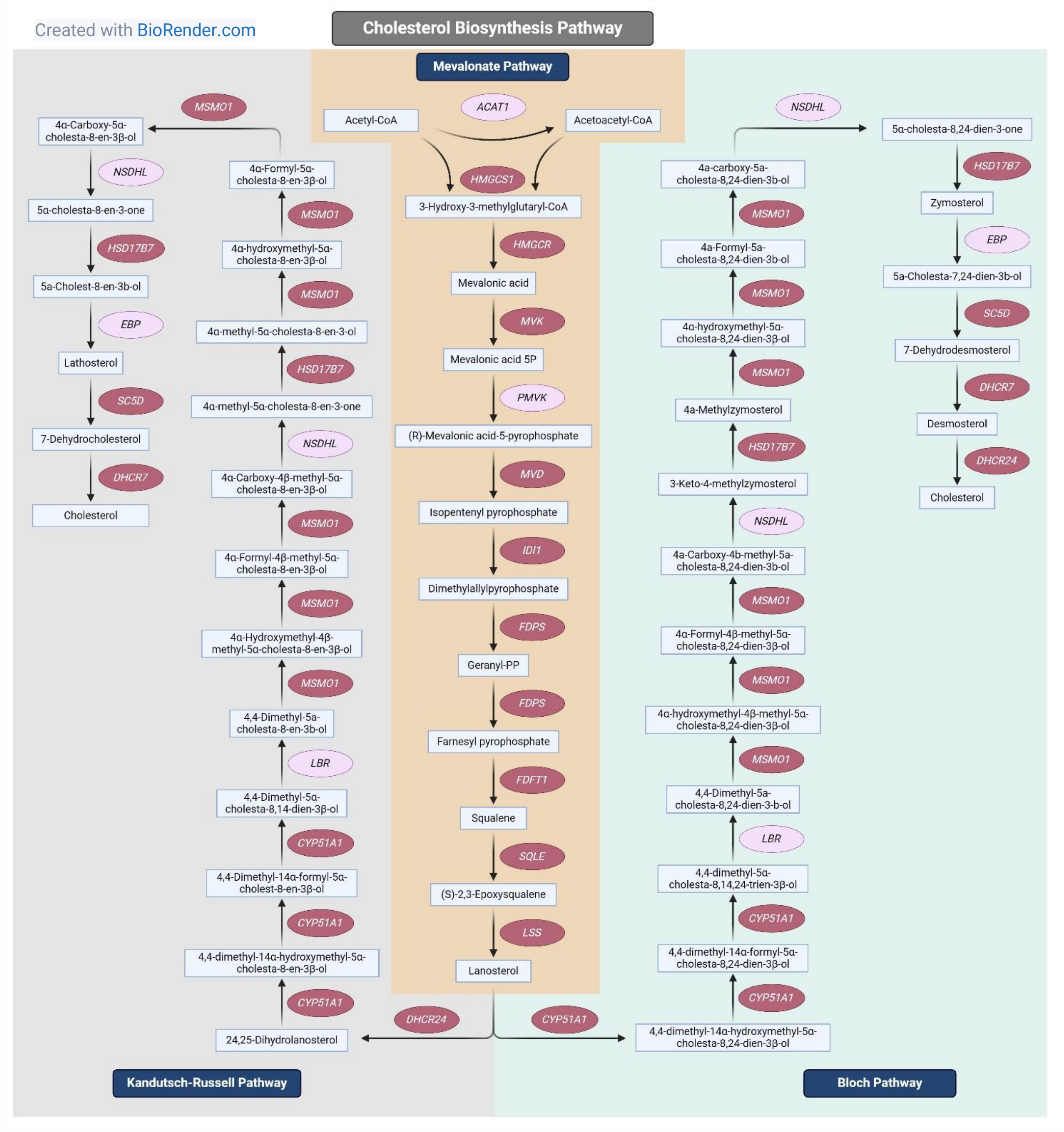

Supplemental Figure 6: Visualization of the Path-MAP identified overlap of differentially expressed genes within the cholesterol biosynthesis pathway. Related to Figure 2. Path-MAP showed an overlap between 9 of 11 enzymes within the mevalonate pathway, 6 out of 9 enzymes within the Bloch Pathway, and 6 of 9 enzymes within the Kandutch-Russel Pathway. Overall, a total of 15 out of 20 enzymes involved in cholesterol biosynthesis were upregulated in our oleic acid exposed non-activated CD4⁺ T cells. Compounds are in blue boxes, enzymes not differentially expressed in the RNA sequencing data are in light pink ovals, enzymes present in the RNA sequencing data are in red ovals, the arrows indicate the direction of movement for cholesterol production, the orange background indicates enzymes and compounds involved in the mevalonate pathway, the grey background indicates enzymes and compounds involved in the Kandutch-Russell pathway, and the light green background indicates enzymes and compounds involved in the Bloch pathway. Visualization created in BioRender.com.

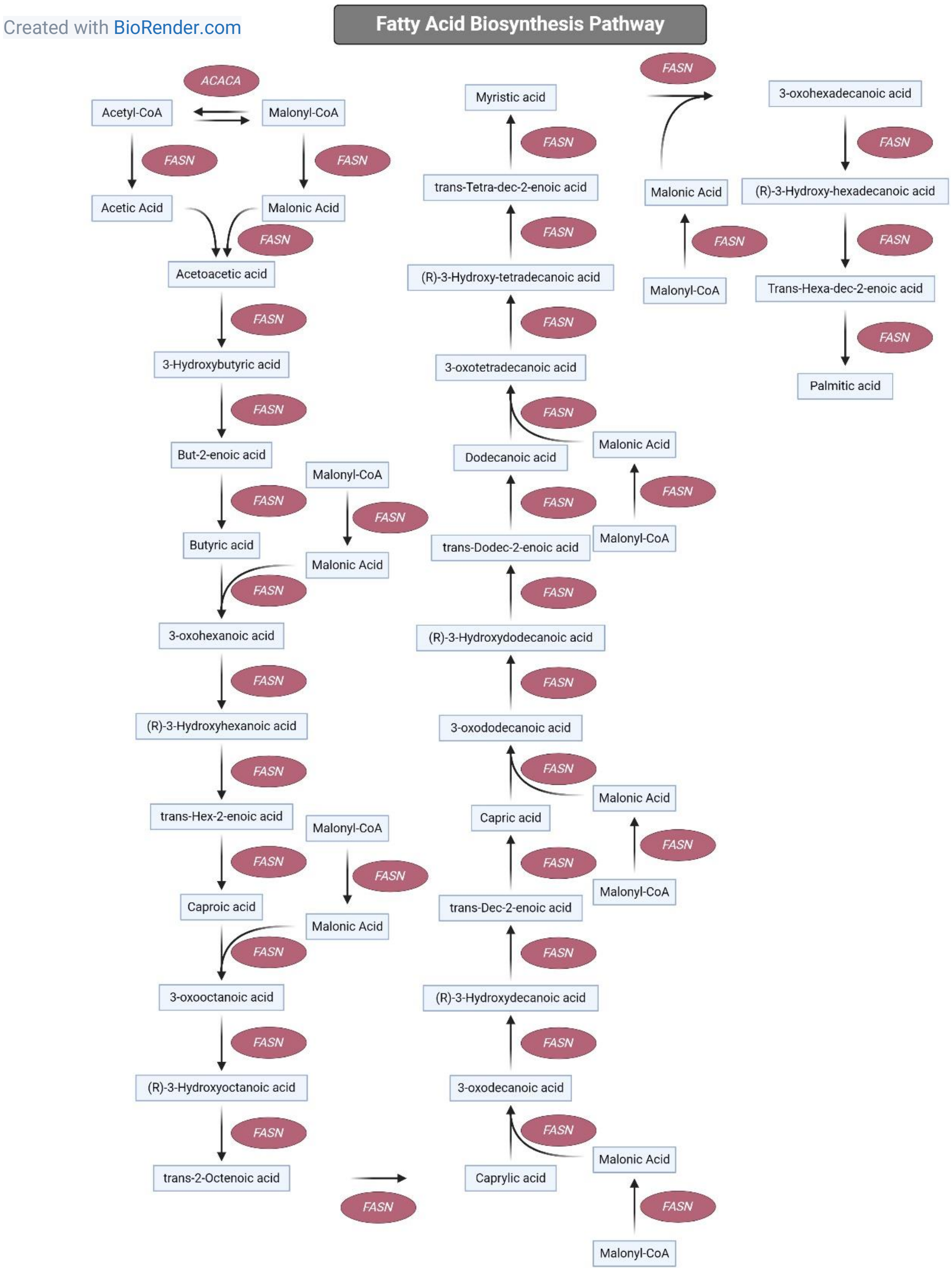

Supplemental Figure 7: Visualization of the Path-MAP identified overlap of differentially expressed genes within the fatty acid biosynthesis pathway. Related to Figure 2. Overall, a total of 2 out of 2 enzymes involved in fatty acid biosynthesis were upregulated in our oleic acid exposed non-activated CD4^{+} T cells. Compounds are in blue boxes, enzymes not differentially expressed in the RNA sequencing data are in light pink ovals, enzymes present in the RNA sequencing data are in red ovals, the arrows indicate the direction of movement for fatty acid production. Visualization created in BioRender.com.

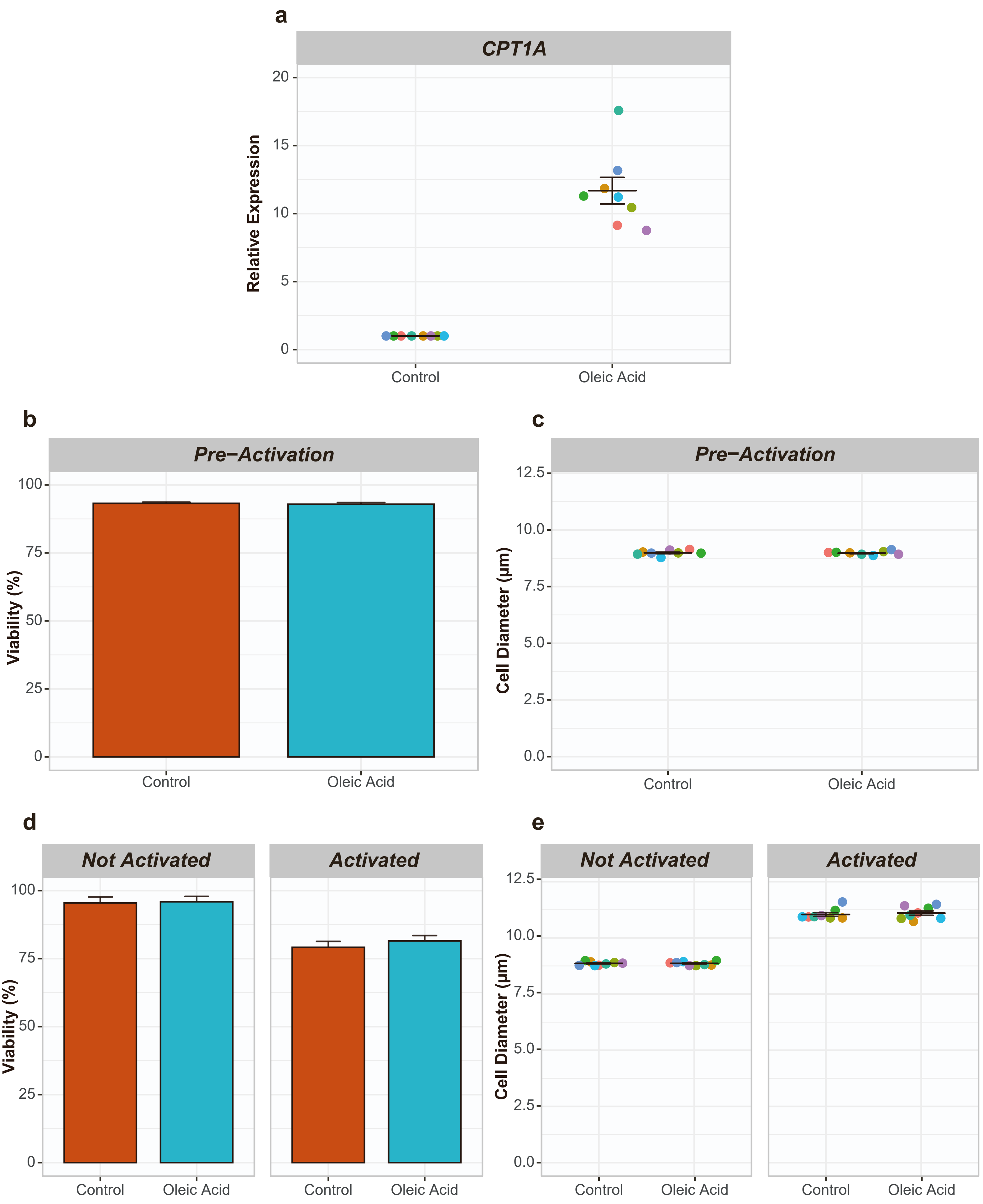

a
CPT1A
Relative Expression
20
15
10
5
0
Control
Oleic Acid
b
Pre−Activation
Viability (%)
100
75
50
25
0
Control
Oleic Acid
c
Pre−Activation
Cell Diameter (µm)
12.5
10.0
7.5
5.0
2.5
0.0
Control
Oleic Acid
d
Not Activated
Activated
Viability (%)
100
75
50
25
0
Control
Oleic Acid
Control
Oleic Acid
e
Not Activated
Activated
Cell Diameter (µm)
12.5
10.0
7.5
5.0
2.5
0.0
Control
Oleic Acid
Control
Oleic Acid

Supplemental Figure 8: Verification of viability, cell diameter and *CPT1A* expression post-exposure and post-activation for spectral cytometry. Related to Figure 3. **(a)** Line plot showing the relative expression of *CPT1A* per donor after 48h of oleic acid exposure as a confirmation of the *in vitro* model by RT-qPCR. Values are colored by donor and shown relative to the control condition. On average *CPT1A* expression of 30µg/mL oleic acid exposed cells was upregulated by 11.68 SE 0.98 fold after 48h ($p < 0.0001$), n =8. **(b)** Bar plot showing the average cell viability and standard error in percent, as determined by Via1-Cassette™ on a NucleoCounter® NC-200™. On average the cell viability of control exposed cells was 93.21 SE 0.43% and of oleic acid exposed cells was 92.89 SE 0.64%. Thus, The solvent control had no effect on $CD4^+$ T cell viability, as expected, at 48h. Thus, there was no effect on $CD4^+$ T cell viability after 48h exposure, n =8. **(c)** Dot plot showing the average cell diameter and standard error in µm, as determined by Via1-Cassette™ on a NucleoCounter® NC-200™. On average the cell diameter of control exposed cells was 8.99 SE 0.04µm and of oleic acid exposed was 8.98 SE 0.03µm. Thus, there was no effect on $CD4^+$ T cell diameter after 48h exposure, n =8. **(d)** Bar plot showing the average cell viability and standard error in percent, as determined by Via1-Cassette™ on a NucleoCounter® NC-200™. Left plot shows the cell viability for non-activated cells and right plot shows the cell viability for activated cells. On average, the cell viability of control pre-exposed non-activated cells was 95.45 SE 2.19% and for 30µg/mL oleic acid pre-exposed non-activated cells was 95.91 SE 1.95% after 72h. The cell viability of control pre-exposed activated cells was 79.13 SE 2.19% and for 30µg/mL oleic acid pre-exposed activated cells was 81.51 SE 1.95% after 72h activation with CD3-CD28 beads. Thus, there was no effect on $CD4^+$ T cell viability between the different pre-exposures. However, activation did affect $CD4^+$ T cell viability, where the activated cells were less viable than the not activated cells, n =8. **(e)** Dot plot showing the average cell diameter and standard error in µm, as determined by Via1-Cassette™ on a NucleoCounter® NC-200™. Left plot shows the cell diameter for non-activated cells and right plot shows the cell diameter for activated cells. On average, the cell diameter of control pre-exposed non-activated cells was 8.86 SE 0.03 µm and for 30µg/mL oleic acid pre-exposed non-activated cells was 8.86 SE 0.03µm after 72h. The cell diameter of control pre-exposed activated cells was 11.04 SE 0.09µm and for 30µg/mL oleic acid pre-exposed activated cells was 11.10 SE 0.10µm after 72h activation with CD3-CD28 beads. Thus, there was no effect on $CD4^+$ T cell diameter between the different pre-exposures. However, activation did affect $CD4^+$ T cell diameter, where the activated cells were larger than the not activated cells, n =8.

Data S9.1: Gating strategy for the primary spectral cytometry analysis of non-activated CD4^{+} T cells pre-exposed to oleic acid

Donor 1 – Control – Not Activated

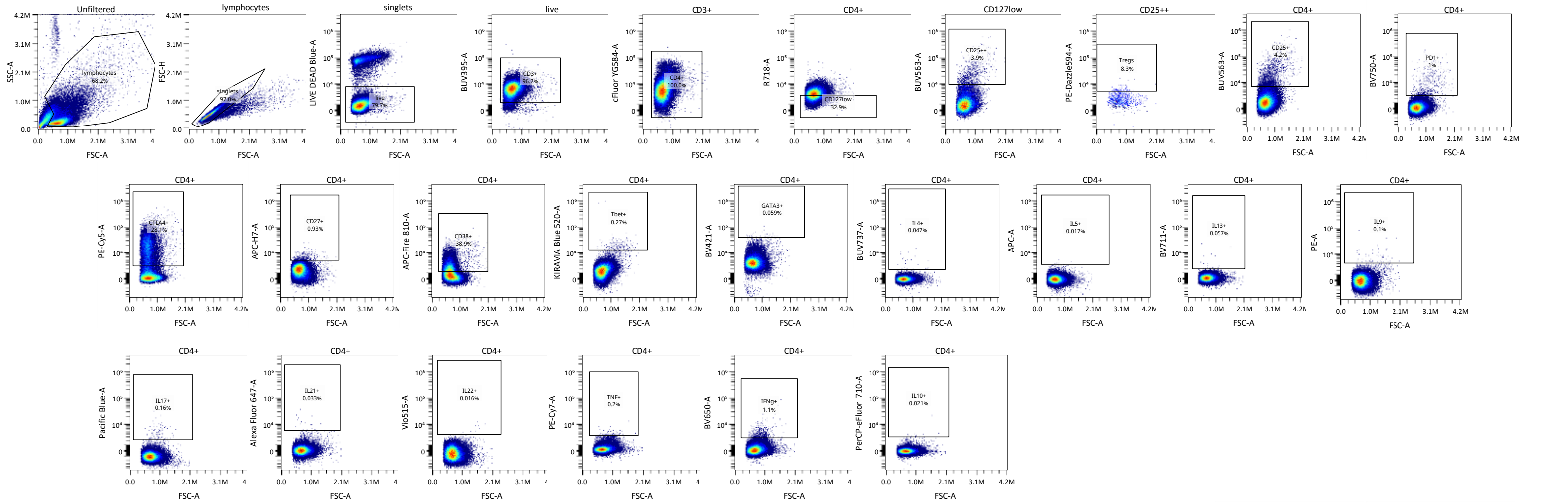

Donor 1 – Oleic Acid – Not Activated

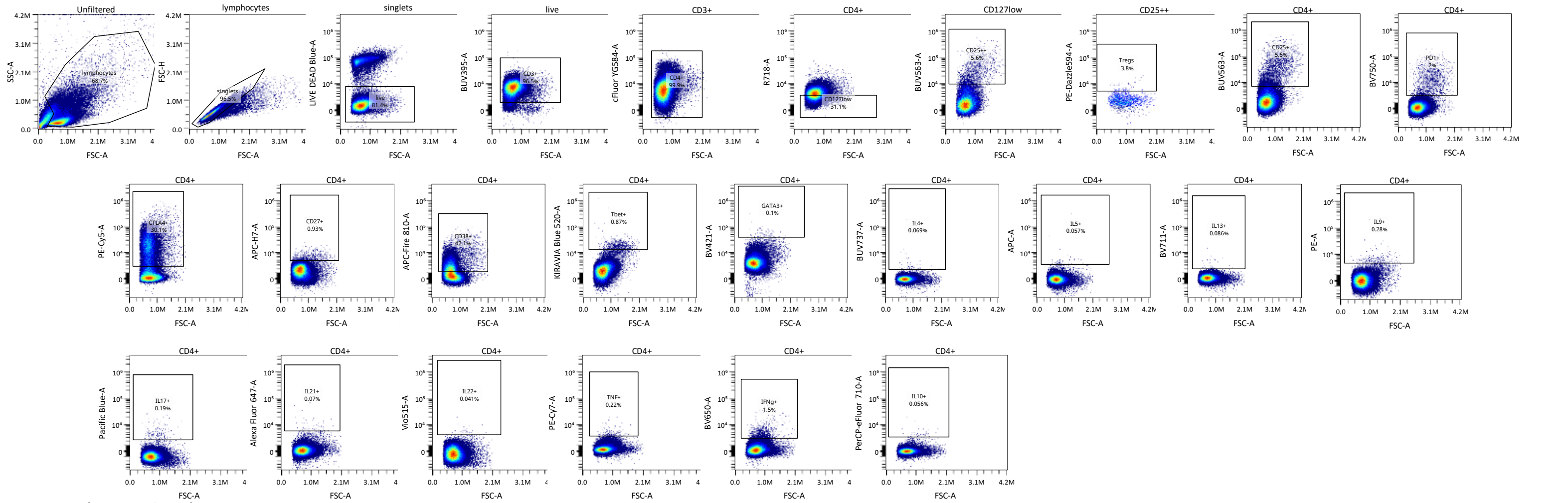

Donor 2 – Control – Not Activated

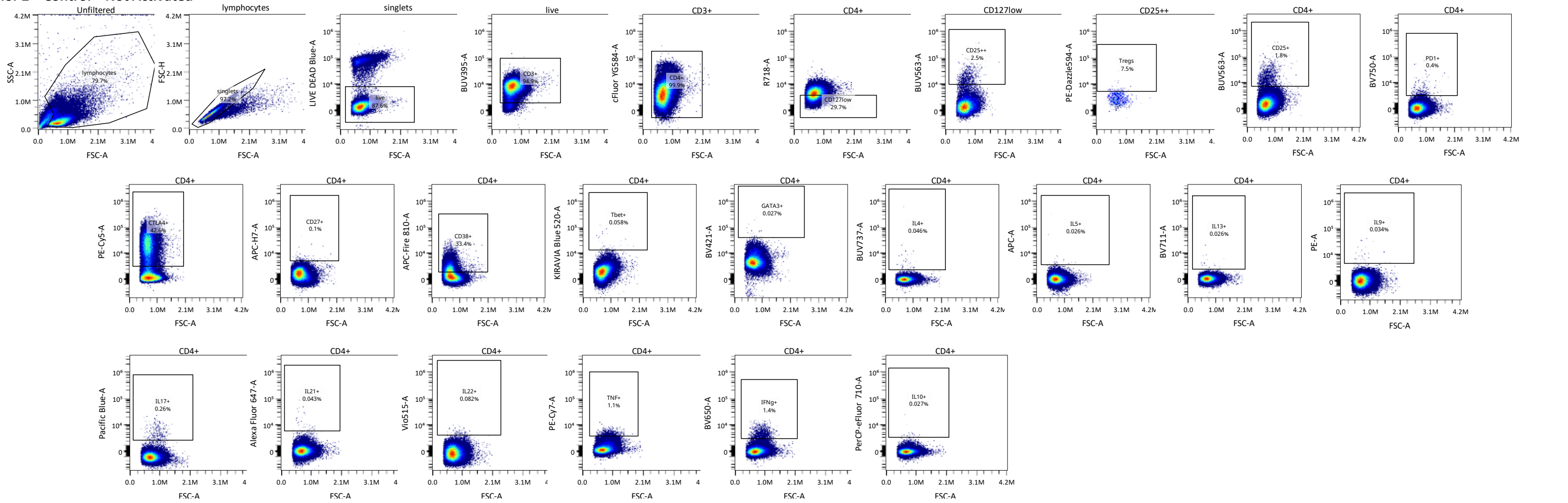

Donor 2 – Oleic Acid – Not Activated

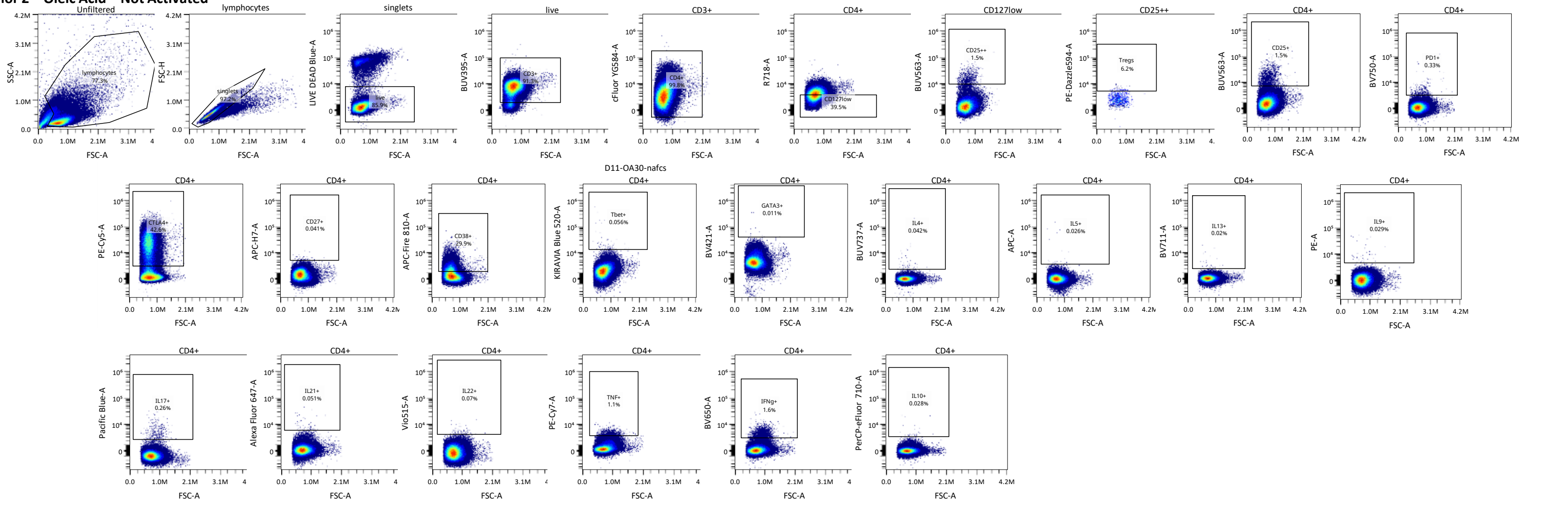

Data S9.2: Gating strategy for the primary spectral cytometry analysis of non-activated $CD4^+$ T cells pre-exposed to oleic acid

Donor 3 – Control – Not Activated

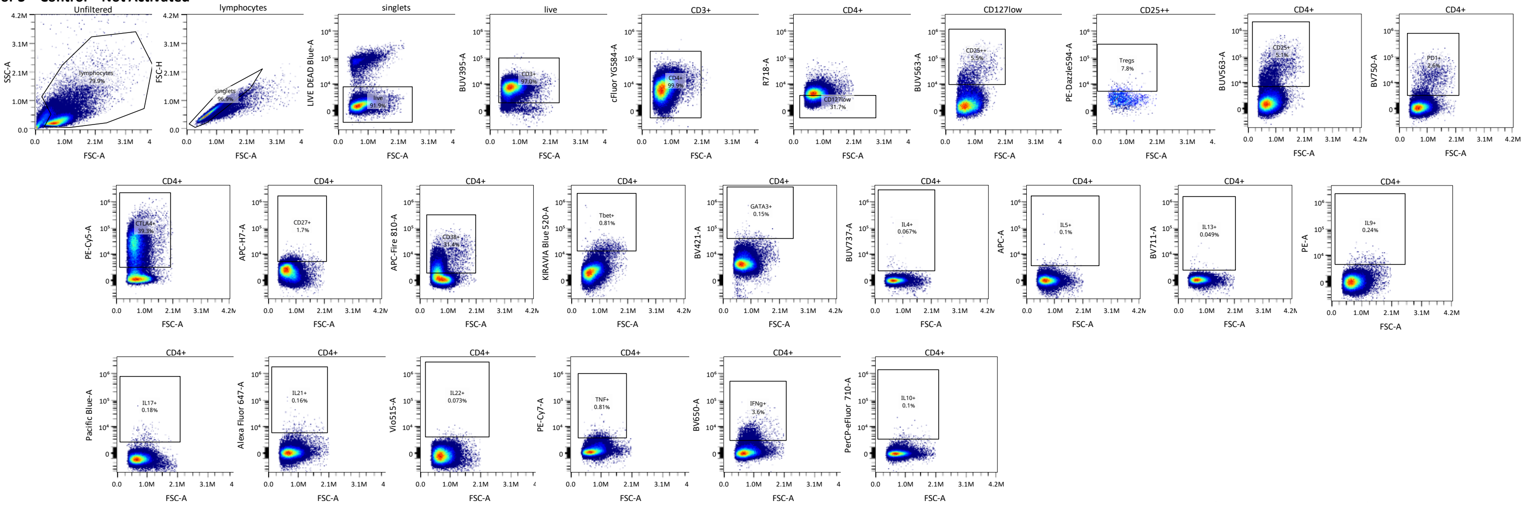

Donor 3 – Oleic Acid – Not Activated

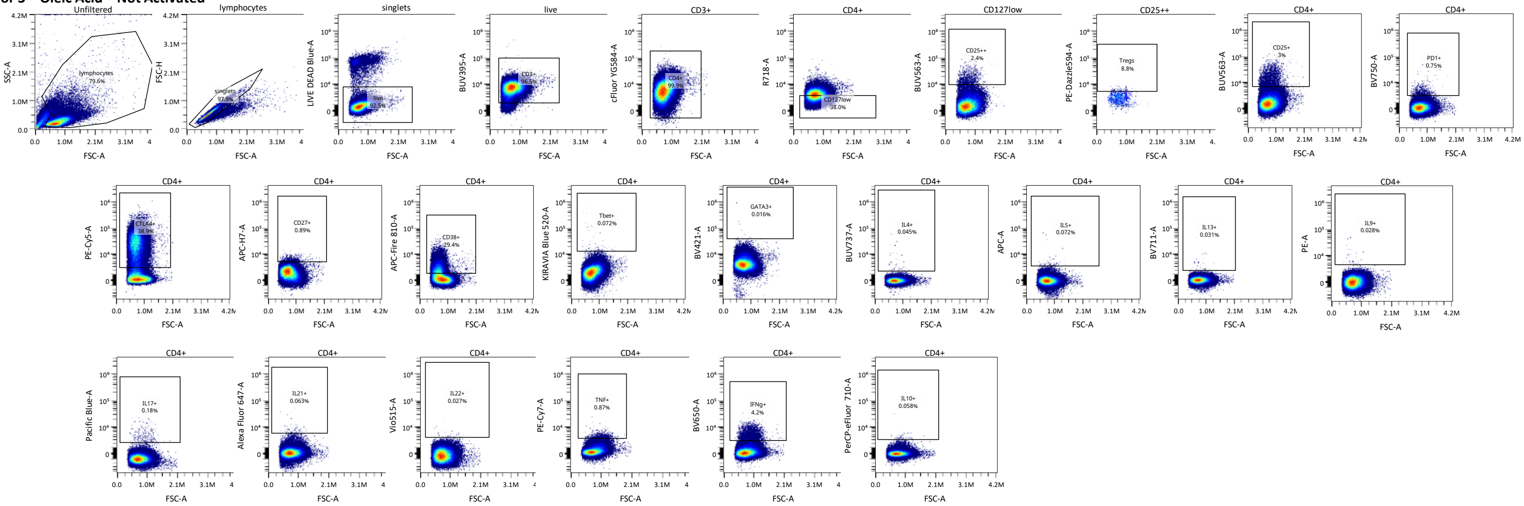

Donor 4 – Control – Not Activated

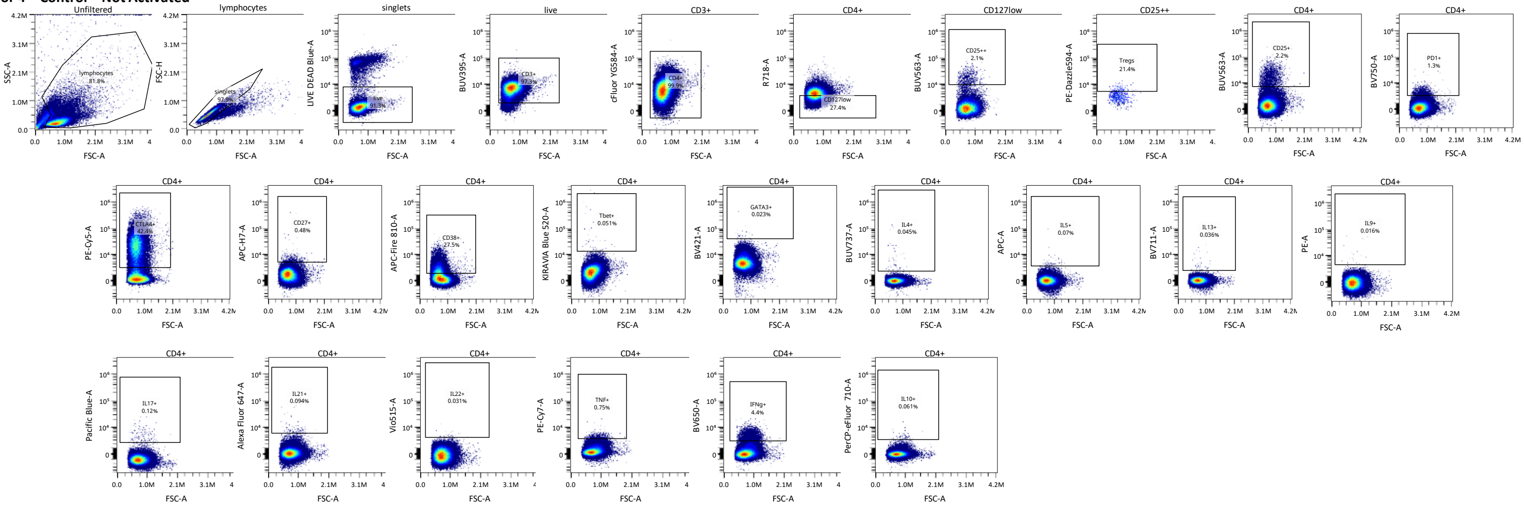

Donor 4 – Oleic Acid – Not Activated

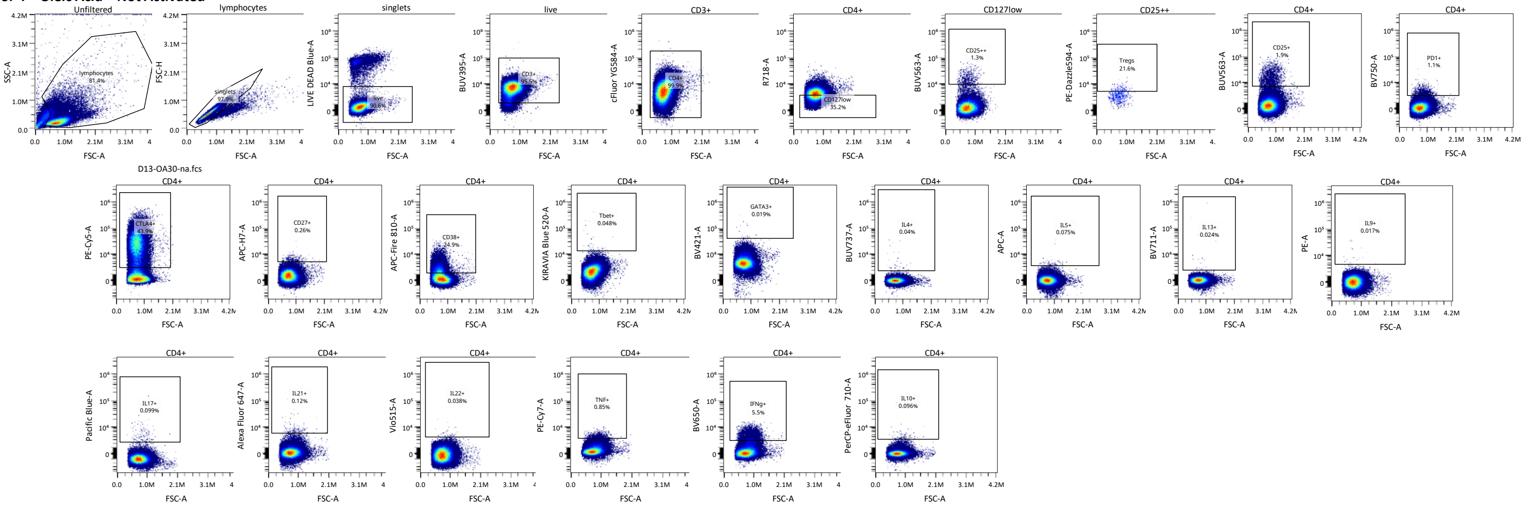

Data S9.3: Gating strategy for the primary spectral cytometry analysis of non-activated CD4^{+} T cells pre-exposed to oleic acid

Donor 5 – Control – Not Activated

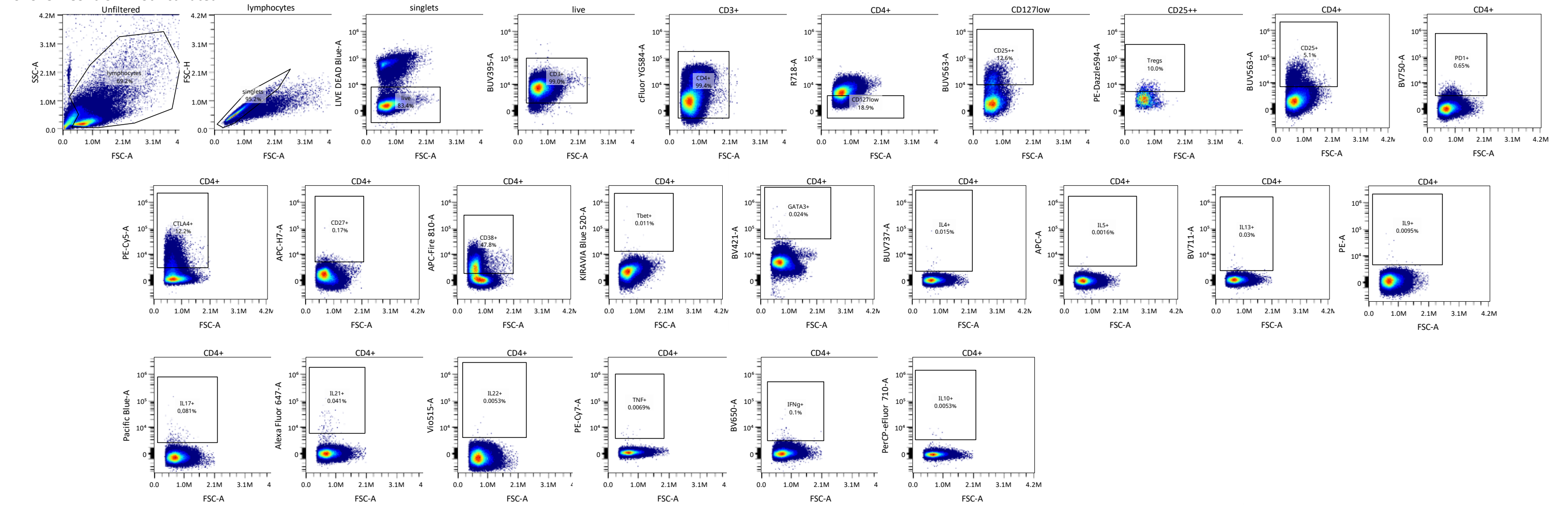

Donor 5 – Oleic Acid – Not Activated

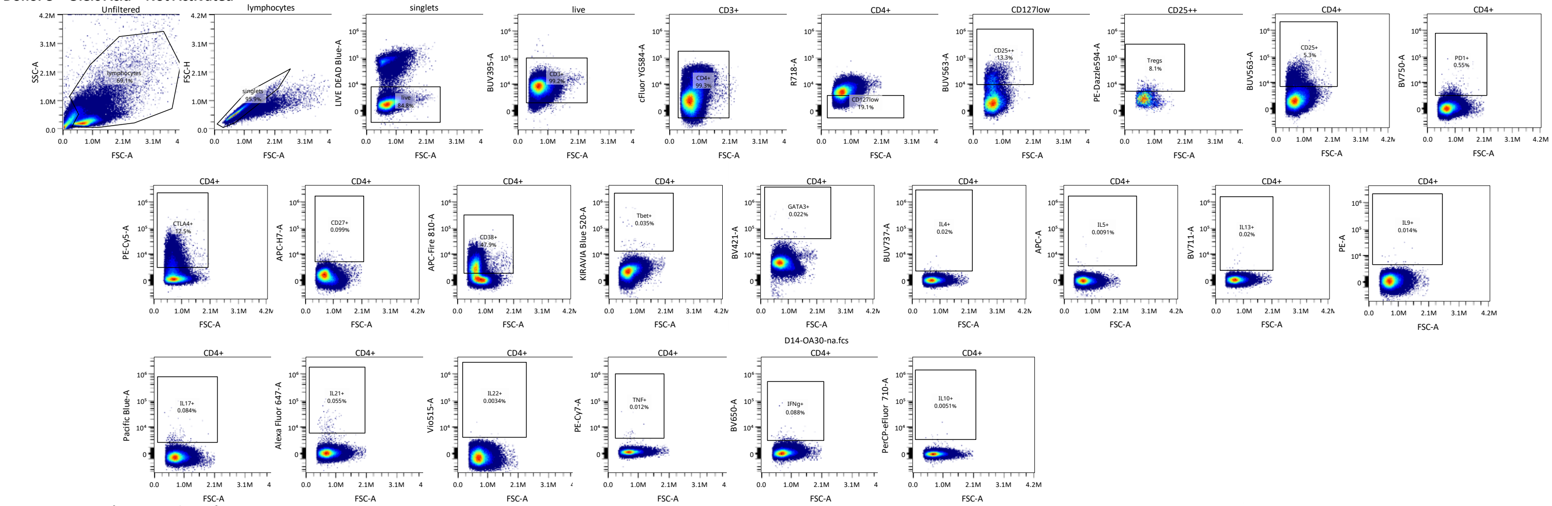

Donor 6 – Control – Not Activated

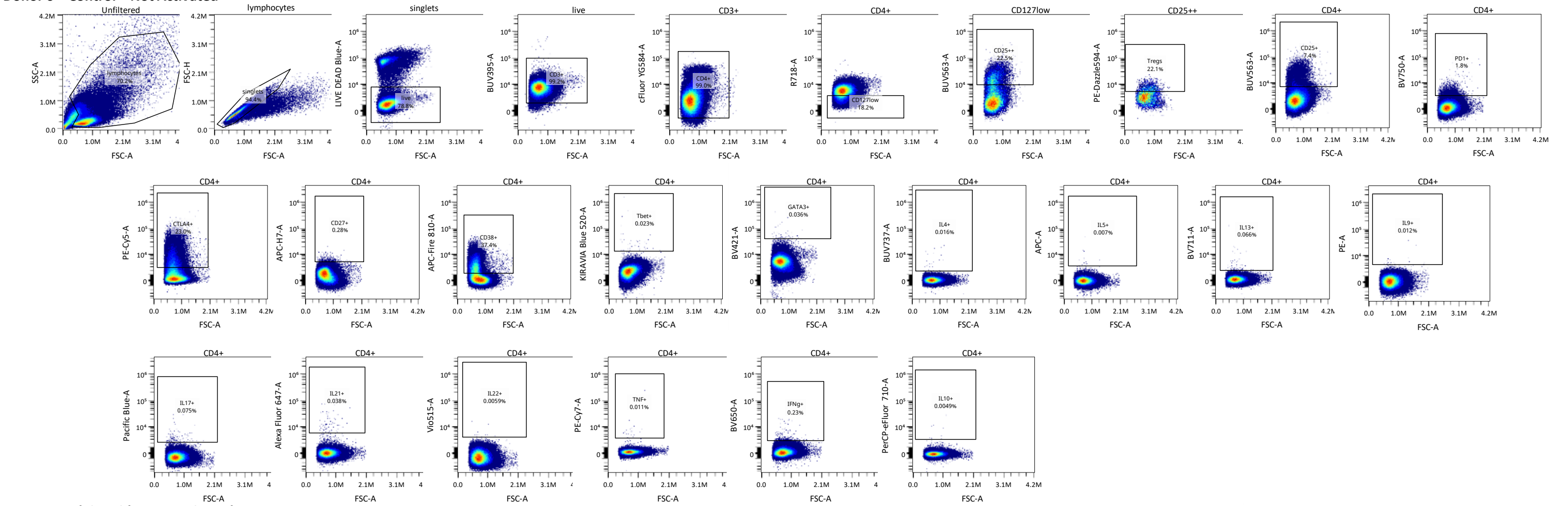

Donor 6 – Oleic Acid – Not Activated

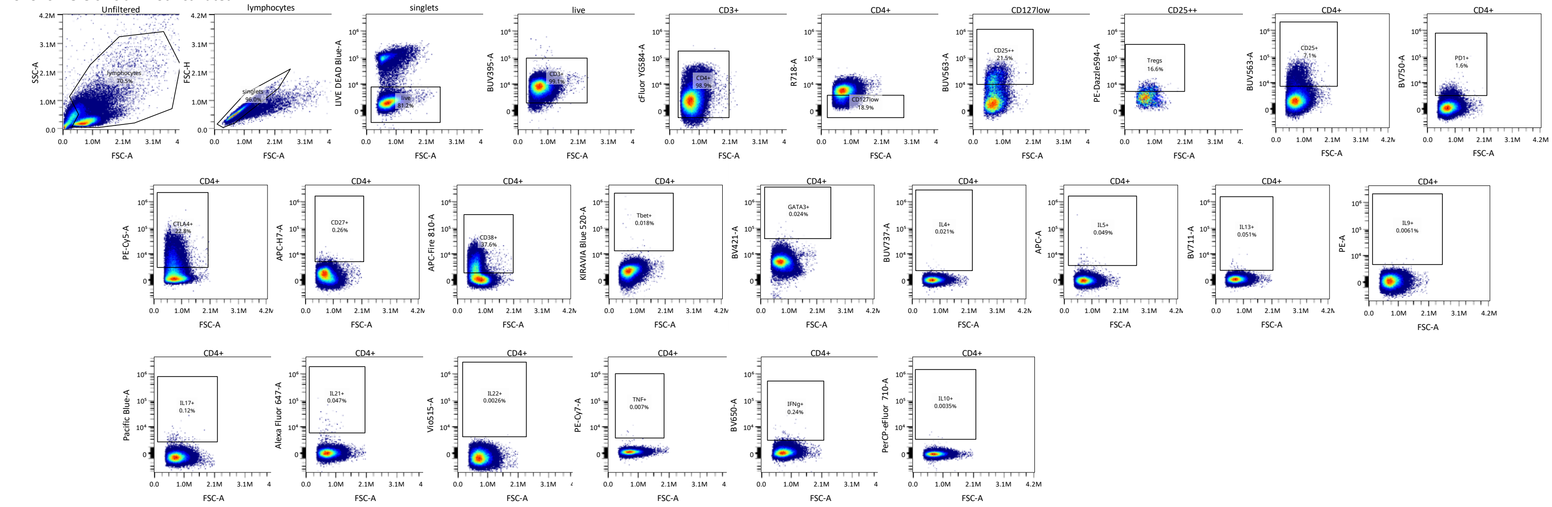

Data S9.4: Gating strategy for the primary spectral cytometry analysis of non-activated CD4⁺ T cells pre-exposed to oleic acid

Donor 7 – Control – Not Activated

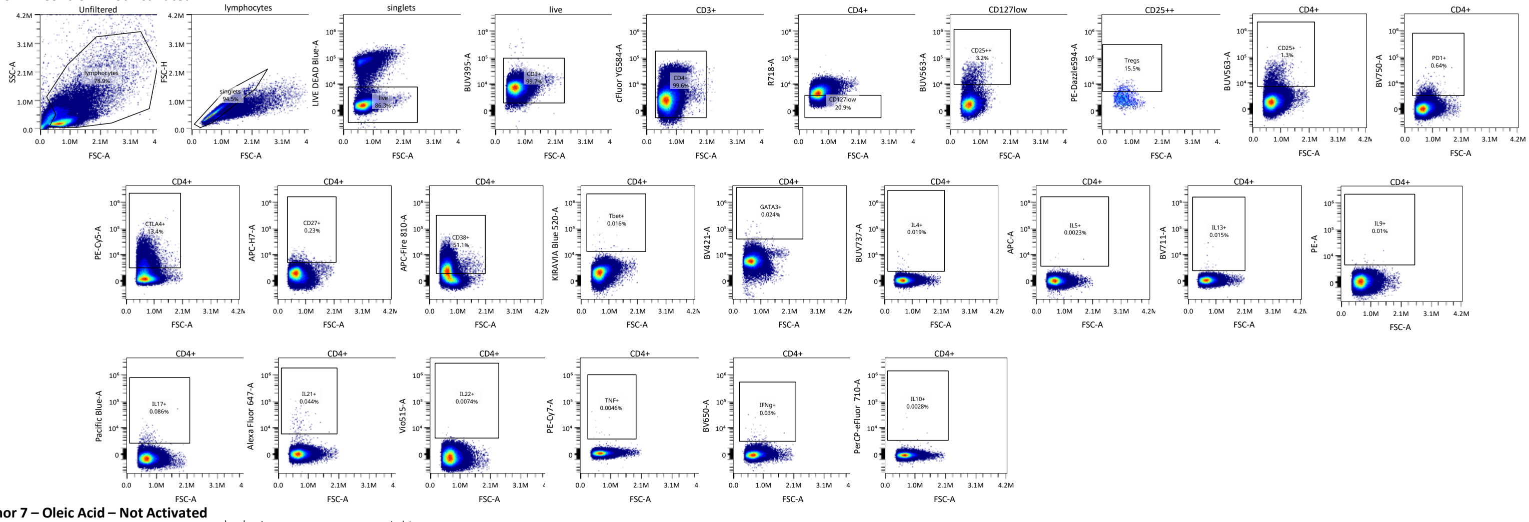

Donor 7 – Oleic Acid – Not Activated

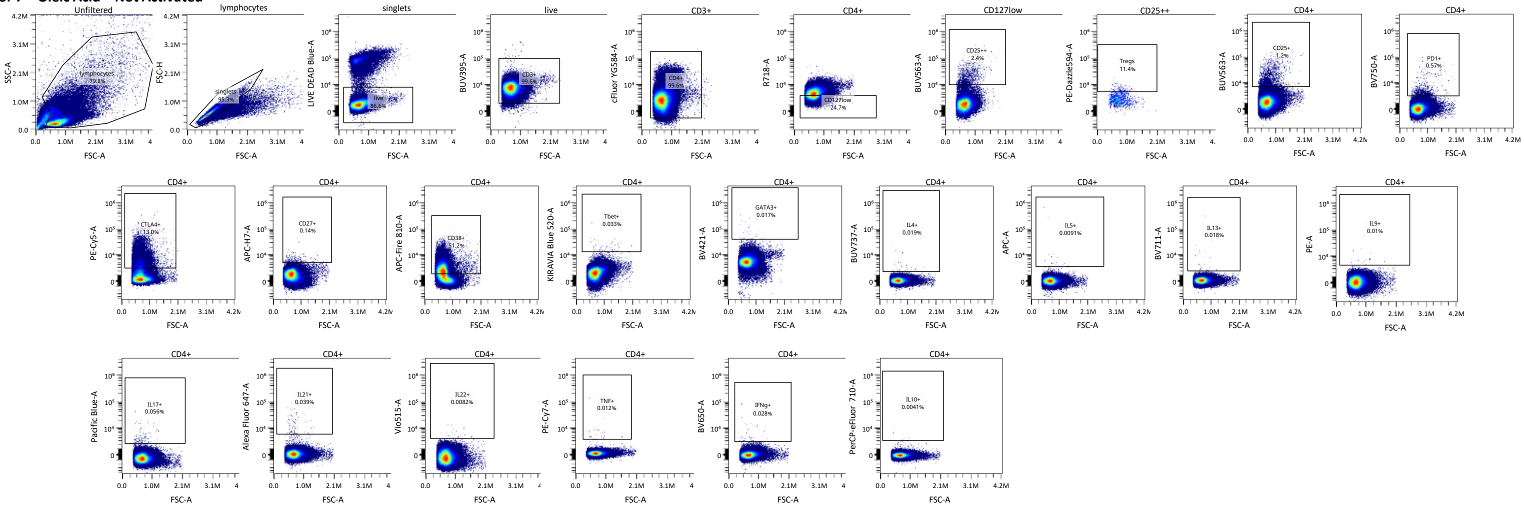

Donor 8 – Control – Not Activated

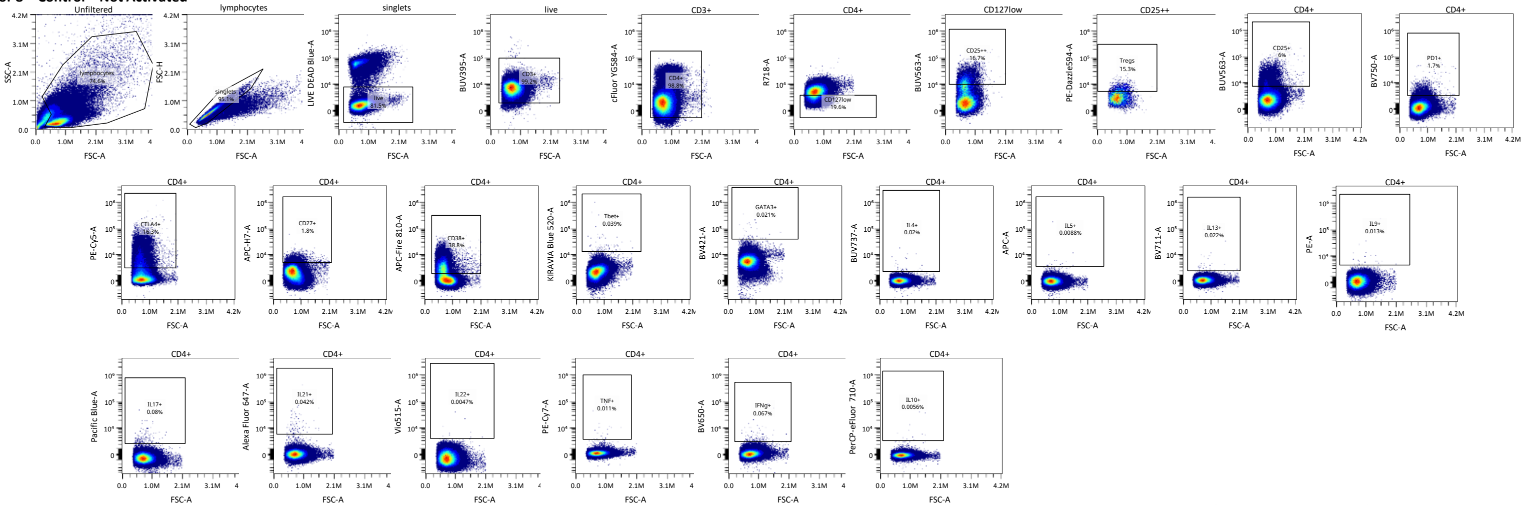

Donor 8 – Oleic Acid – Not Activated

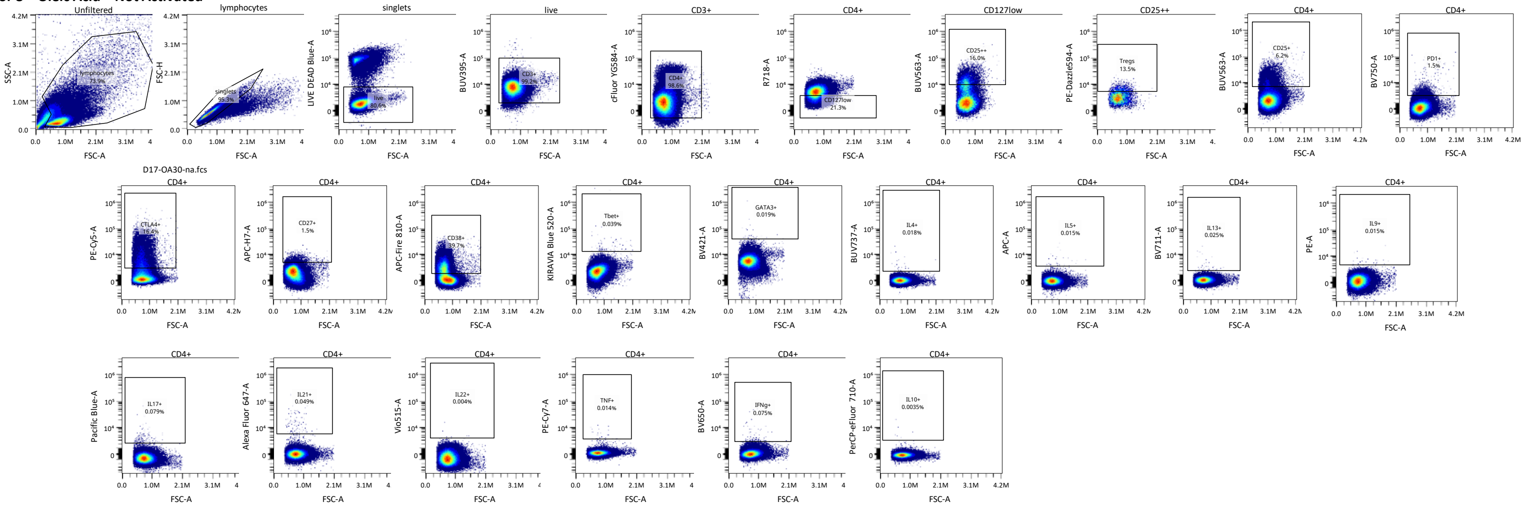

Supplemental Figure 9: Gating strategy for the primary spectral cytometry analysis of non-activated $CD4^+$ T cells pre-exposed to oleic acid, n = 8, related to Figure 3. Gating strategy is the same for all 8 donors analyzed and includes gates set for all markers measured in the panel.

Data S10.1: Gating strategy for the primary spectral cytometry analysis of activated CD4⁺ T cells pre-exposed to oleic acid

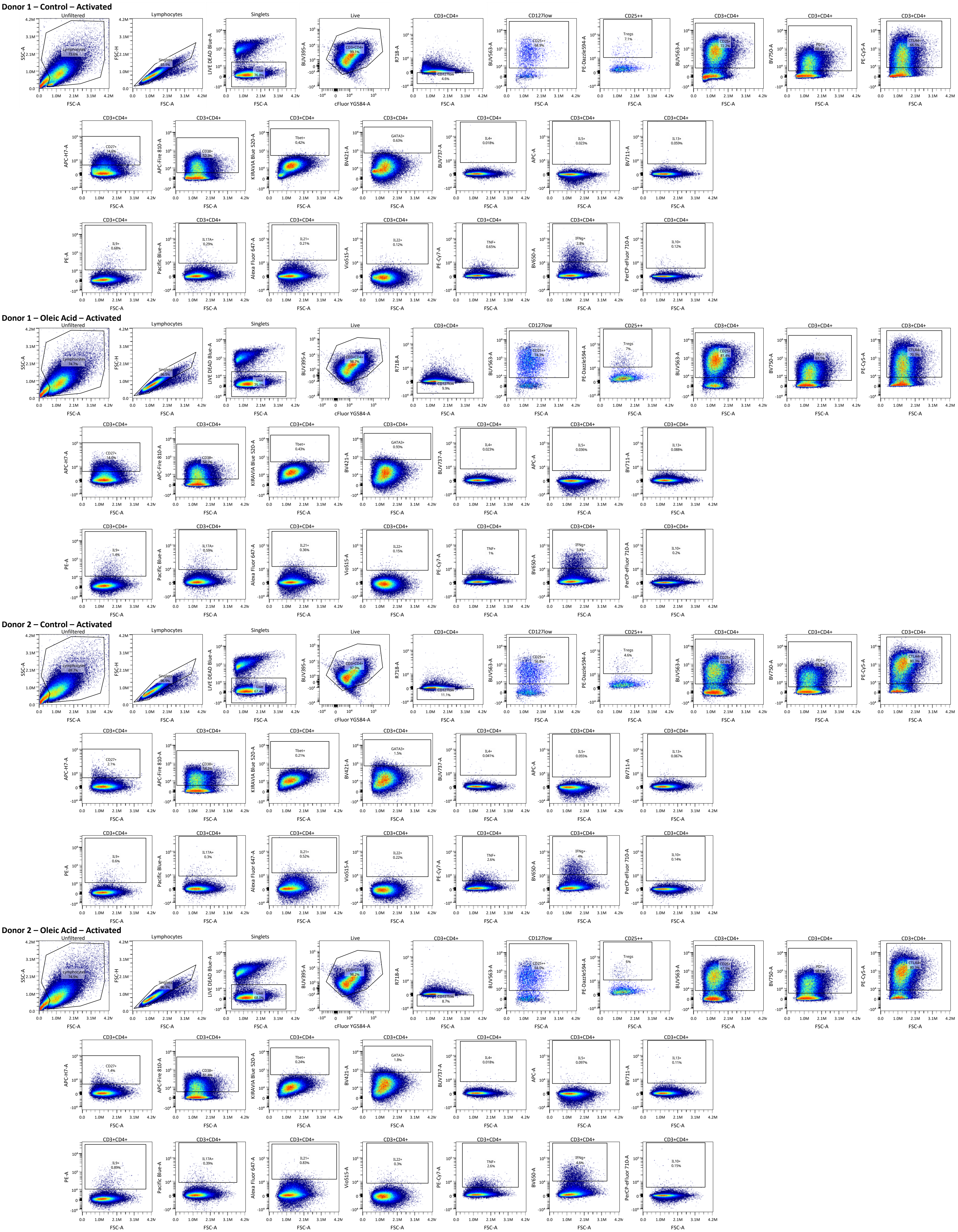

Data S10.2: Gating strategy for the primary spectral cytometry analysis of activated CD4+ T cells pre-exposed to oleic acid

Donor 3 – Control – Activated

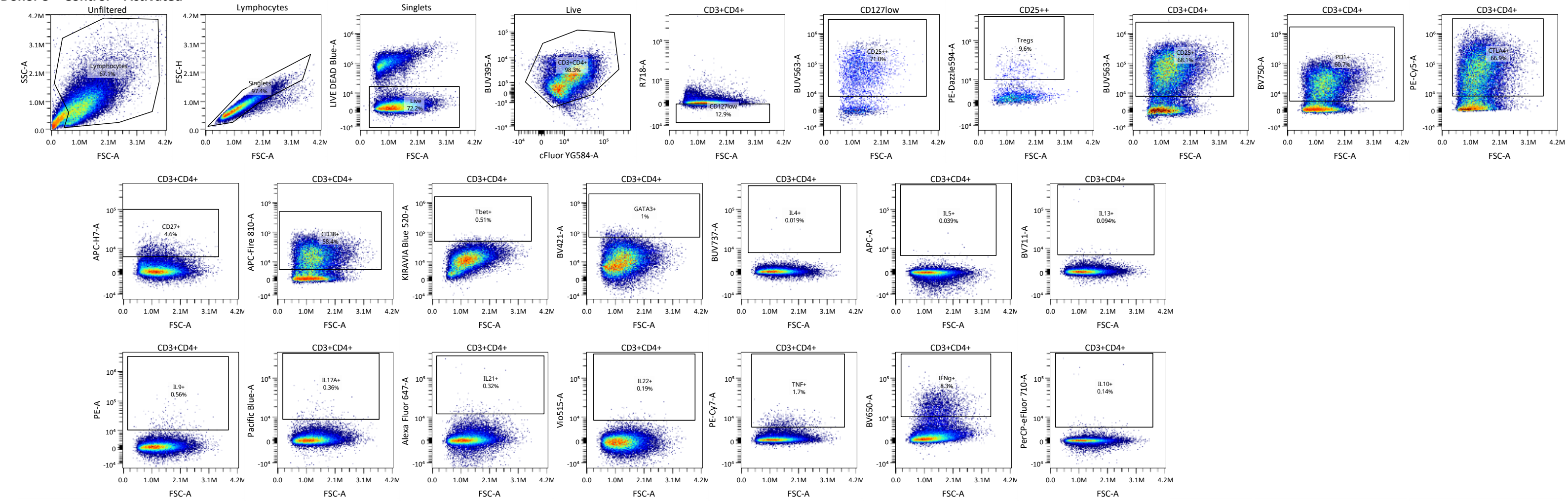

Donor 3 – Oleic Acid – Activated

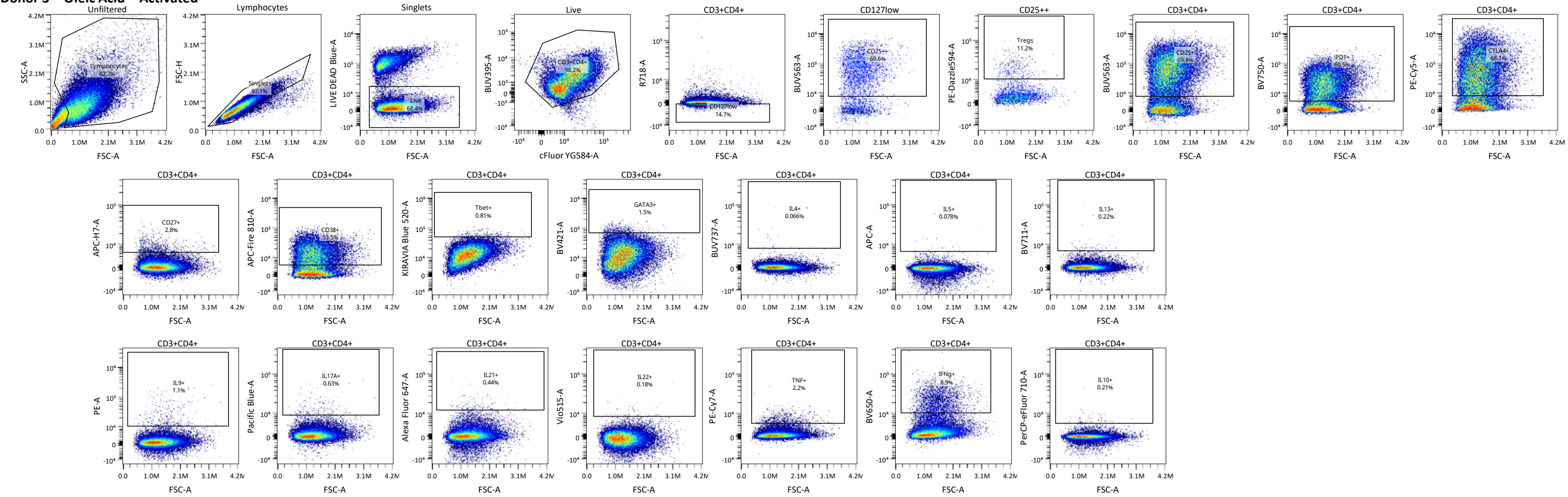

Donor 4 – Control – Activated

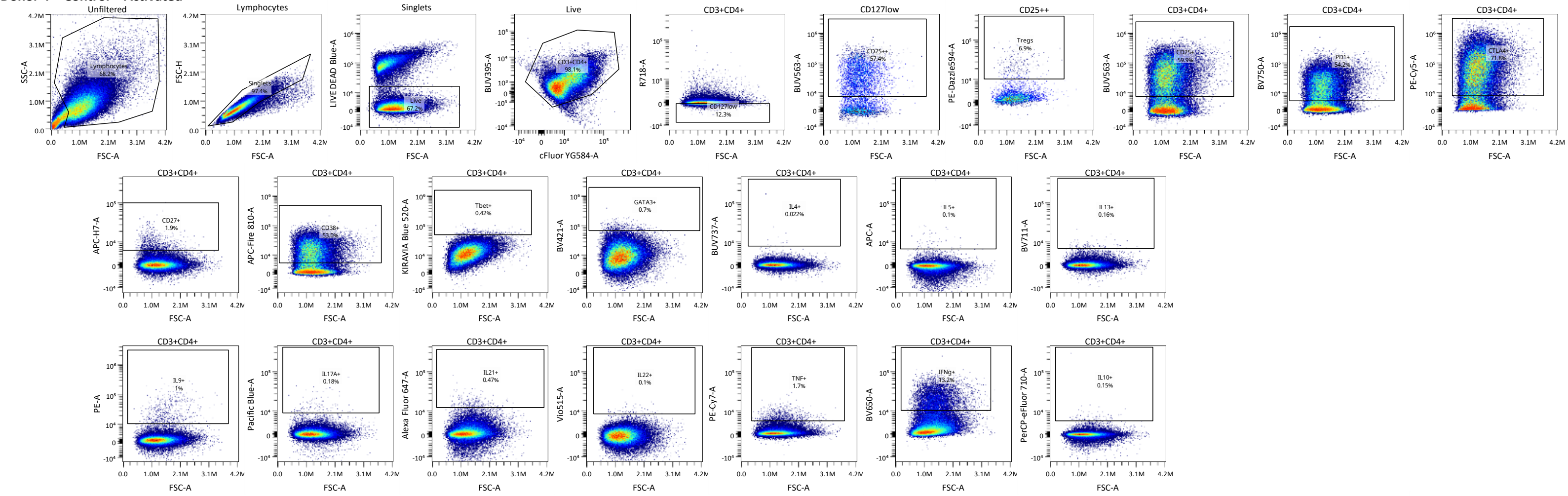

Donor 4 – Oleic Acid – Activated

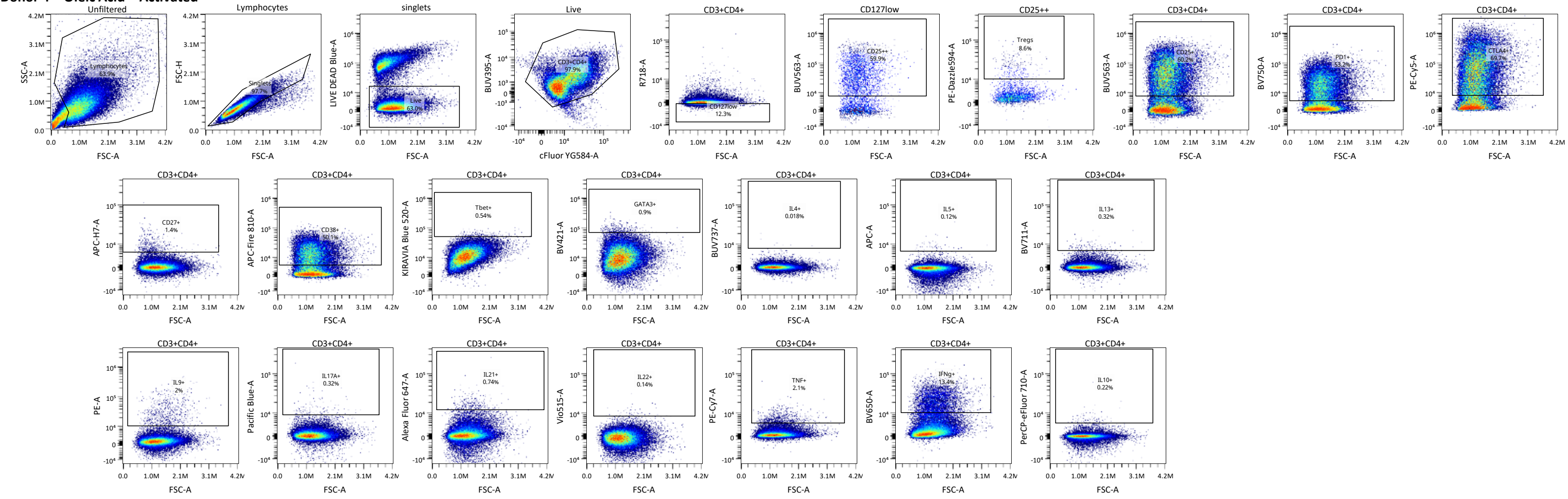

Data S10.3: Gating strategy for the primary spectral cytometry analysis of activated CD4⁺ T cells pre-exposed to oleic acid

Donor 5 – Control – Activated

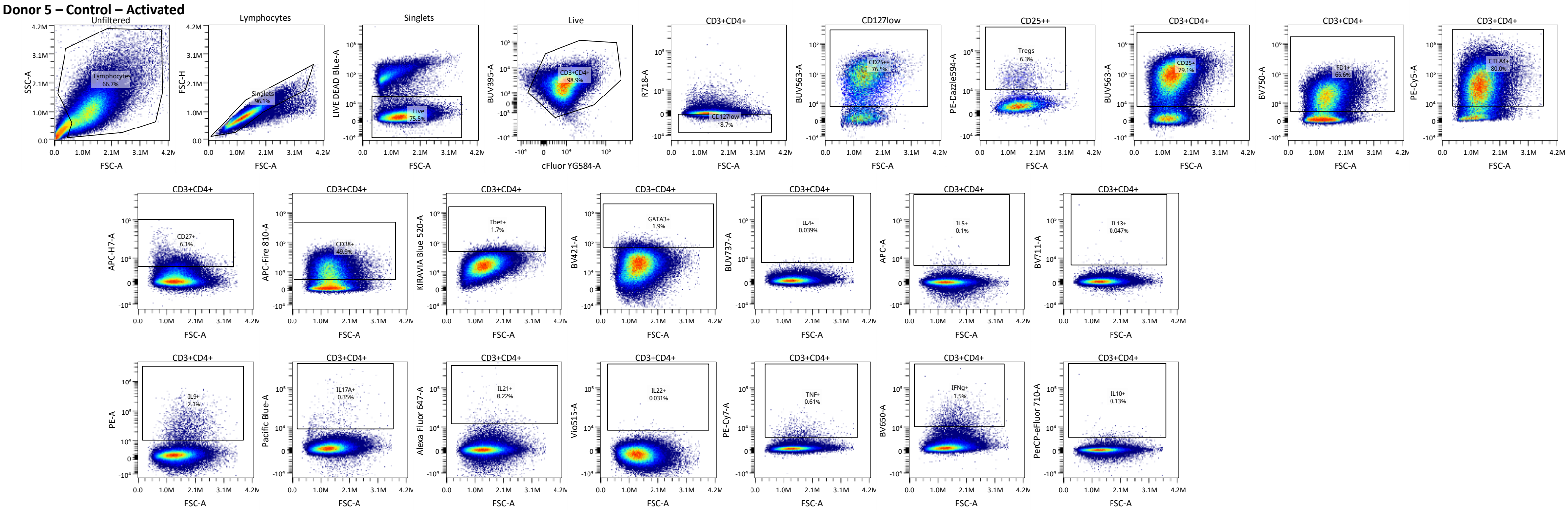

Donor 5 – Oleic Acid – Activated

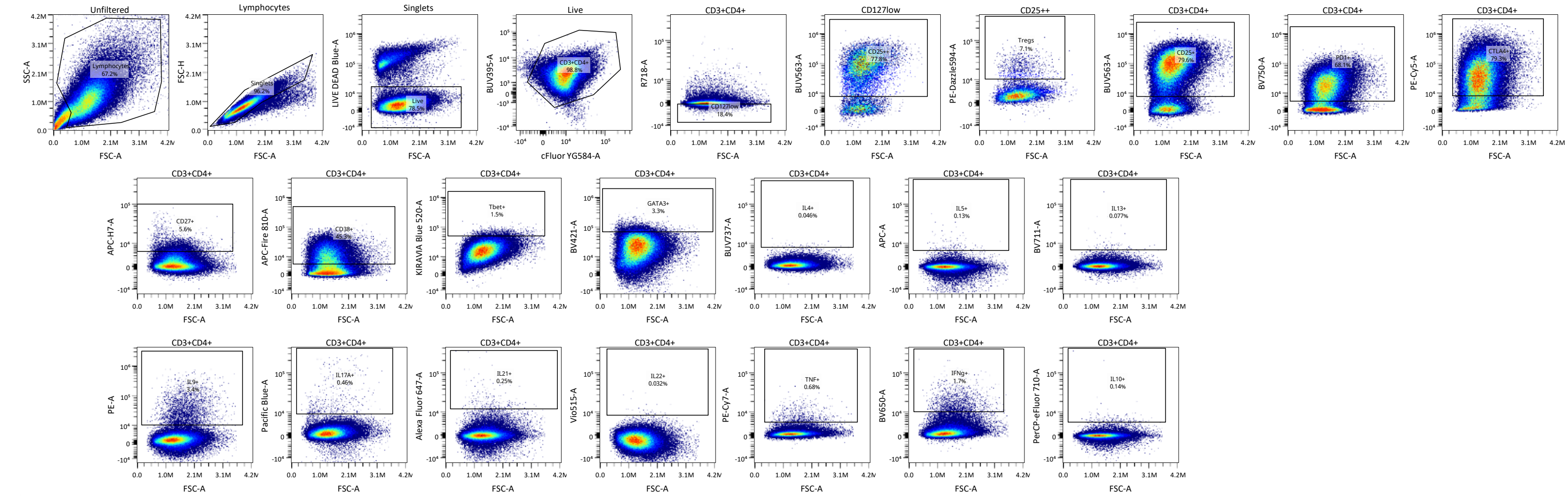

Donor 6 – Control – Activated

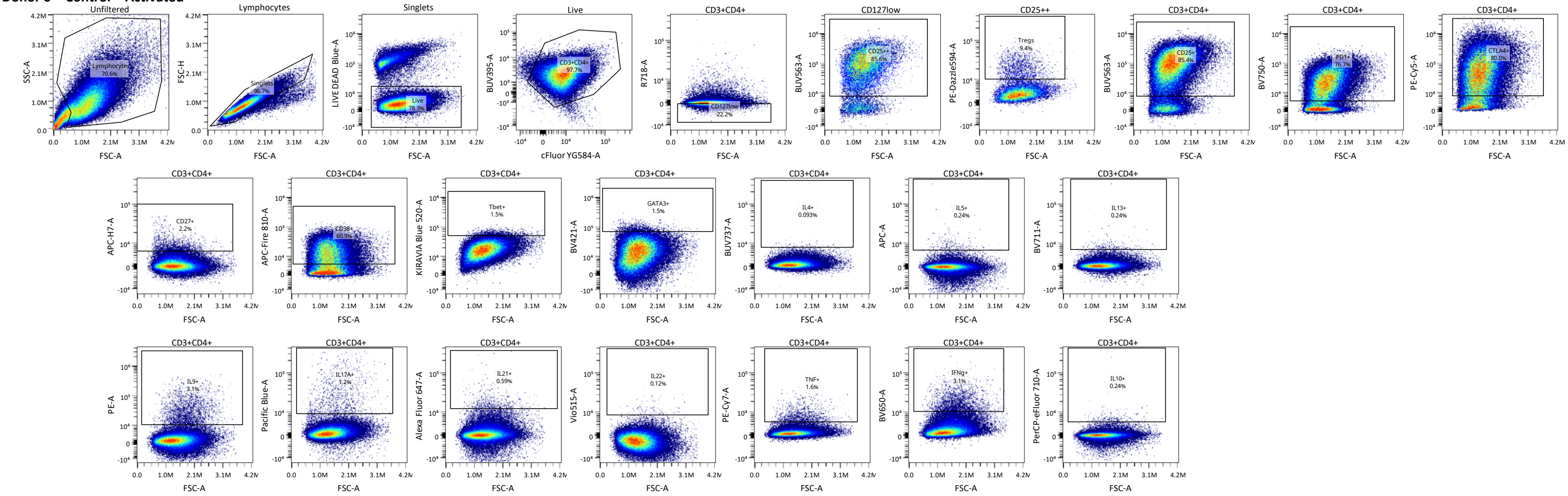

Donor 6 – Oleic Acid – Activated

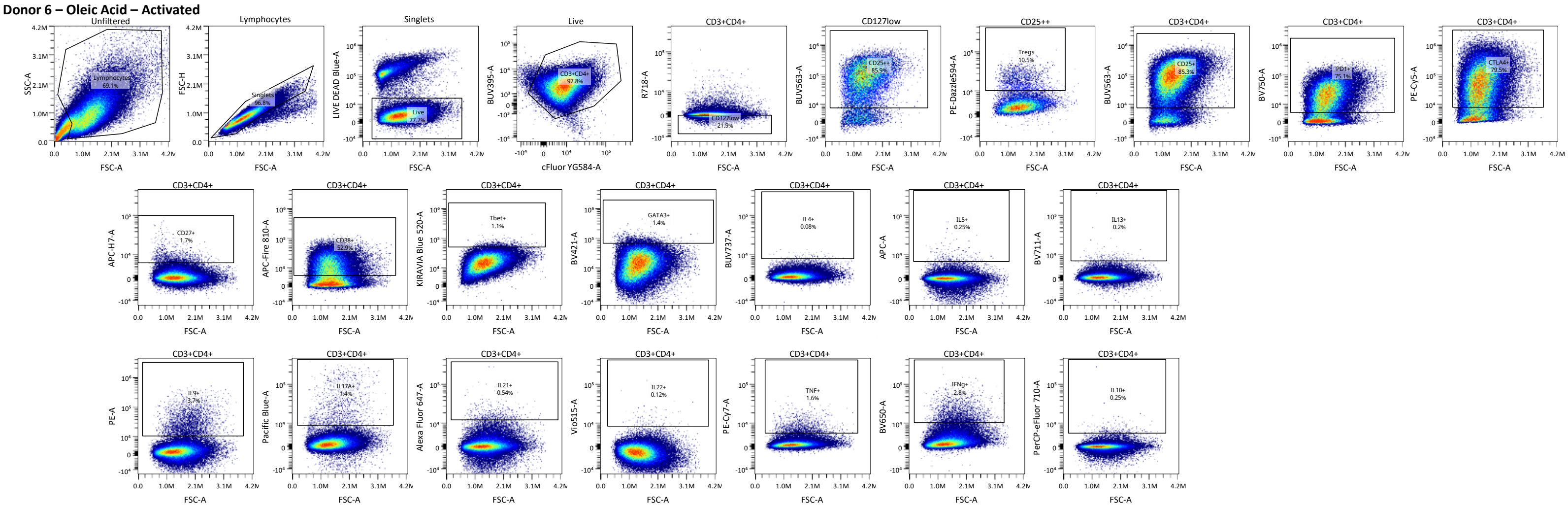

Data S10.4: Gating strategy for the primary spectral cytometry analysis of activated CD4$^+$ T cells pre-exposed to oleic acid

Donor 7 – Control – Activated

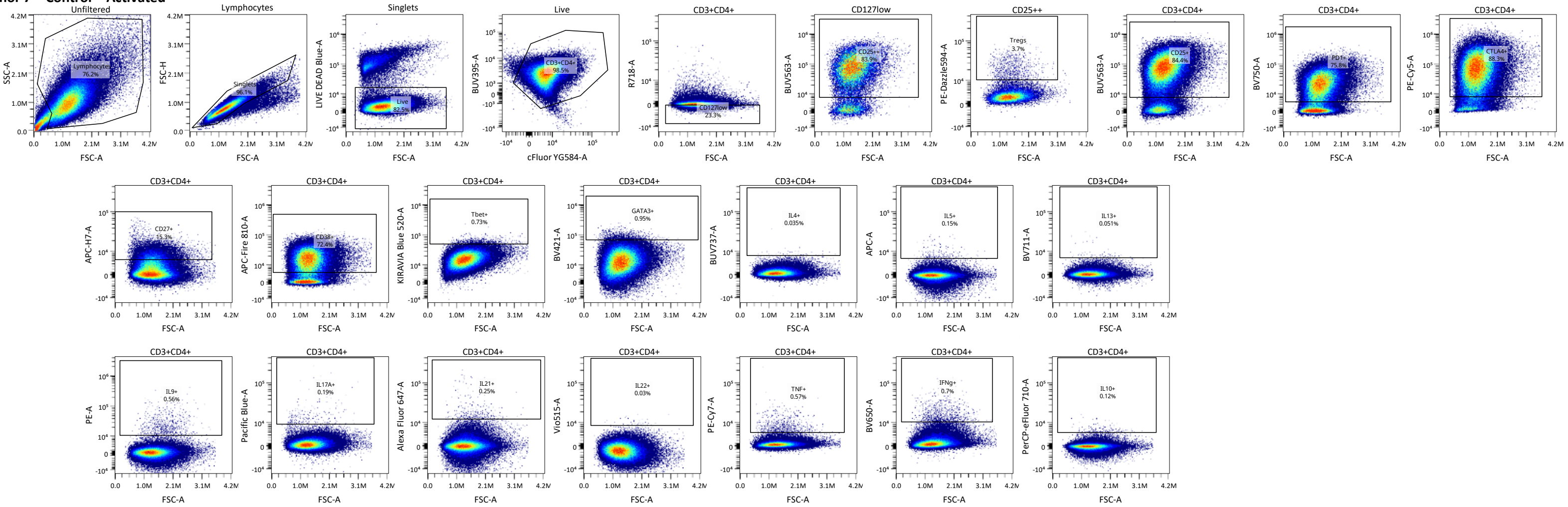

Donor 7 – Oleic Acid – Activated

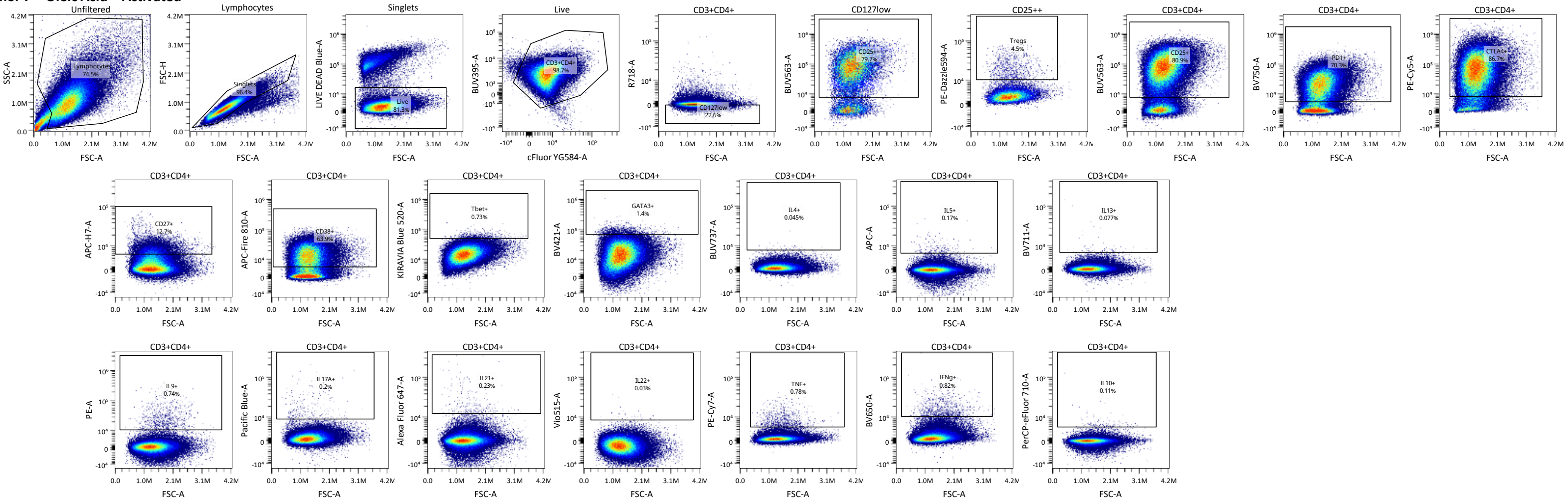

Donor 8 – Control – Activated

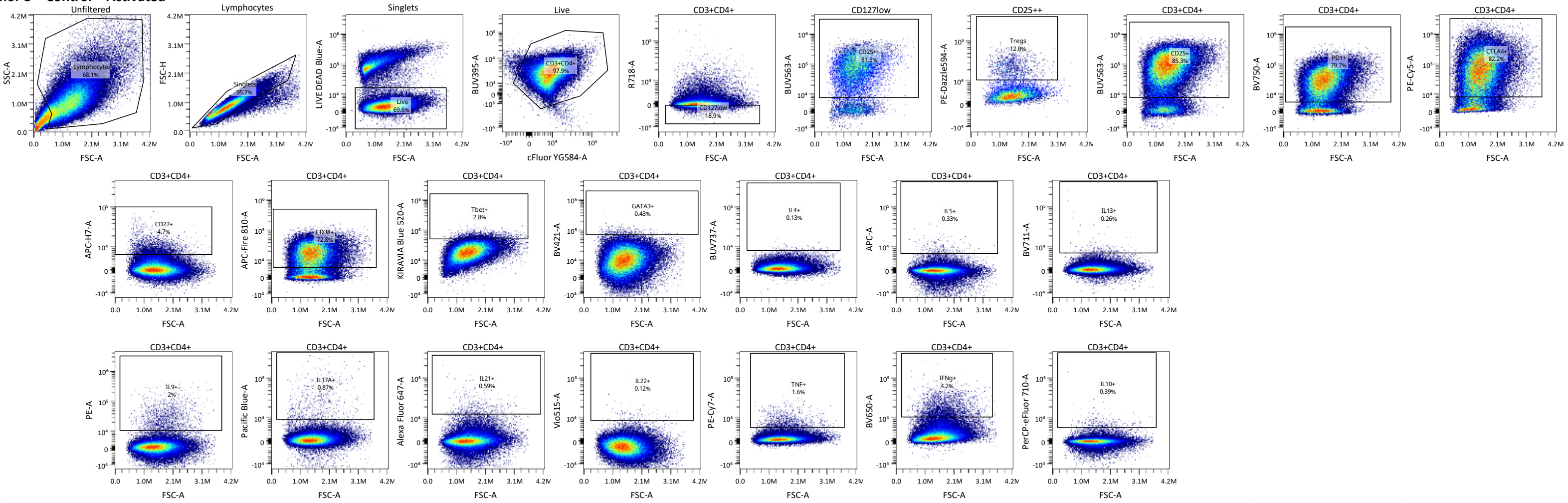

Donor 8 – Oleic Acid – Activated

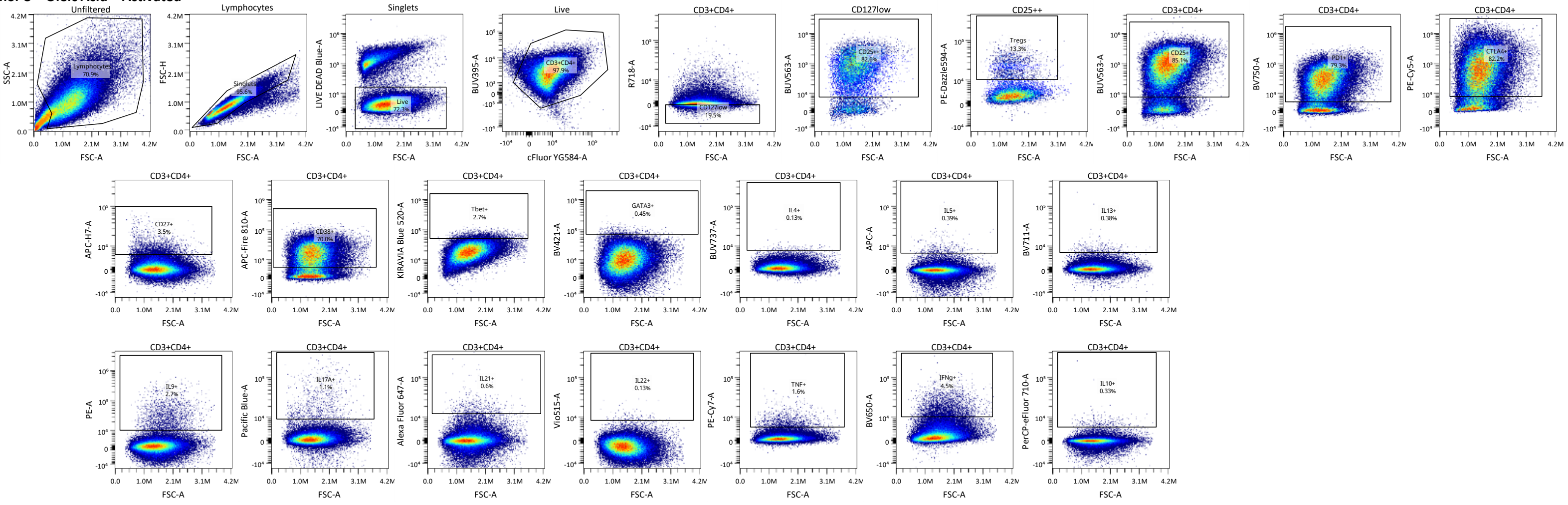

Supplemental Figure 10: Gating strategy for the primary spectral cytometry analysis of activated CD4$^+$ T cells pre-exposed to oleic acid, n = 8, related to Figure 3. Gating strategy is the same for all 8 donors analyzed and includes gates set for all markers measured in the panel.

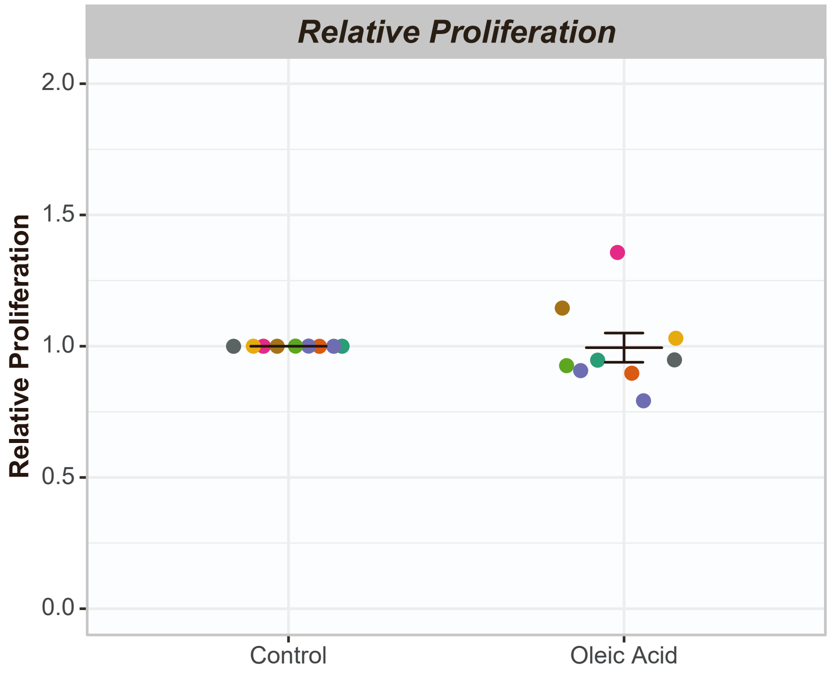
Relative Proliferation
Relative Proliferation
2.0
1.5
1.0
0.5
0.0
Control
Oleic Acid

Supplemental Figure 11: Relative proliferation post-activation of oleic acid pre-exposed $CD4^+$ T cells. Related to Figure 3. Dot plot showing the relative proliferation, as determined by ^3H incorporation, per donor after 48h exposure to control or 30µg/mL oleic acid, where the solvent was completely evaporated prior to addition to the cells, and subsequent activation by CD3/CD28 activation beads for 72h. ^3H was added post 72h activation and cells were left to proliferate for 18h. Data is shown relative to the Control condition. Points are colored by donor with mean and standard error. No difference in proliferative capacity was observed between the oleic acid and control pre-exposed conditions. On average the relative proliferation of oleic acid exposed cells was 0.99 SE 0.06 ($p > 0.92$), $n = 9$.

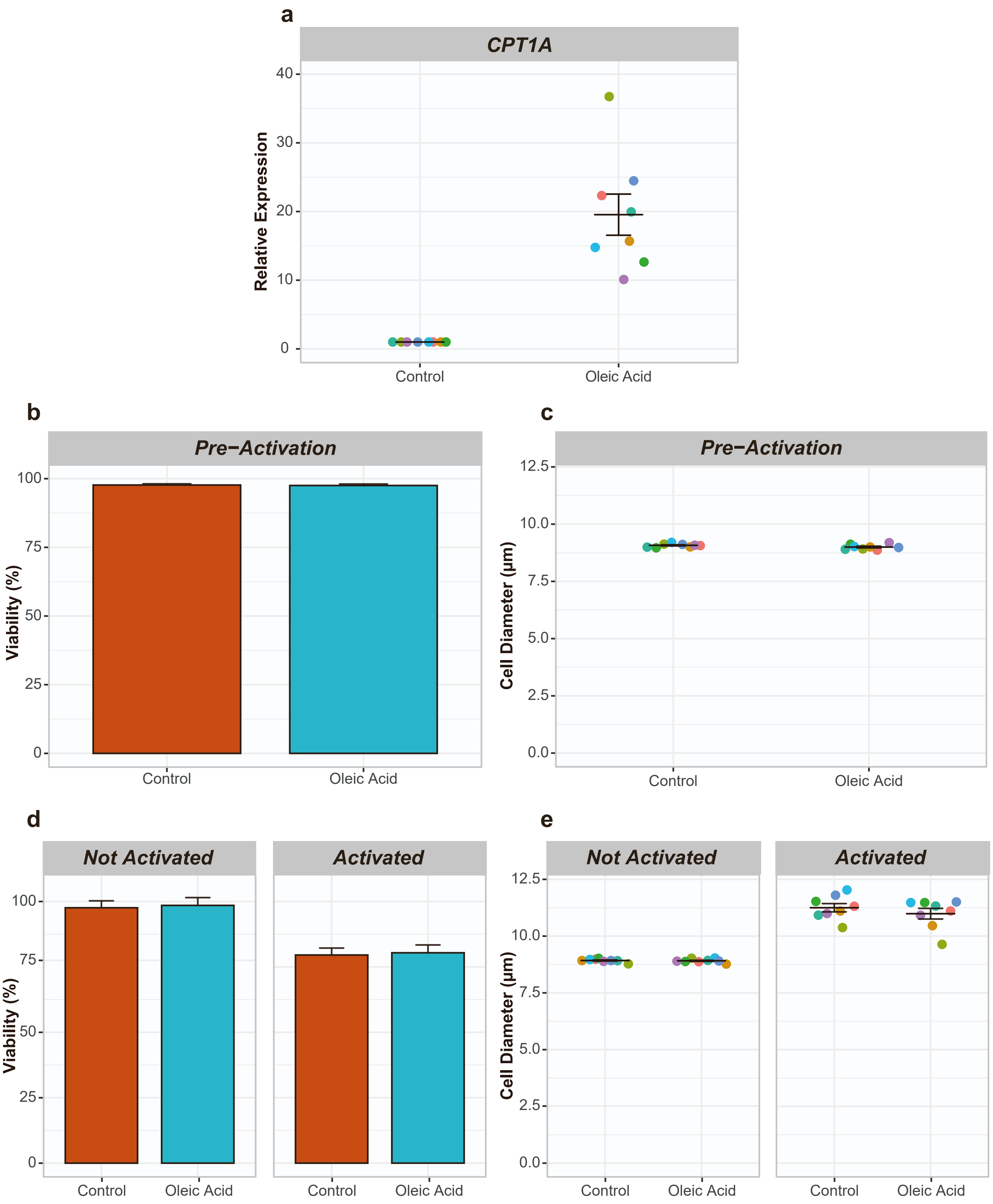

a
CPT1A
Relative Expression
0
10
20
30
40
Control
Oleic Acid
b
Pre−Activation
Viability (%)
0
25
50
75
100
Control
Oleic Acid
c
Pre−Activation
Cell Diameter (µm)
0.0
2.5
5.0
7.5
10.0
12.5
Control
Oleic Acid
d
Not Activated
Activated
Viability (%)
0
25
50
75
100
Control
Oleic Acid
Control
Oleic Acid
e
Not Activated
Activated
Cell Diameter (µm)
0.0
2.5
5.0
7.5
10.0
12.5
Control
Oleic Acid
Control
Oleic Acid

Supplemental Figure 12: Verification of viability, cell diameter and *CPT1A* expression post-exposure and post-activation for spectral cytometry. Related to Figure 3. **(a)** Line plot showing the relative expression of *CPT1A* per donor after 48h of oleic acid exposure as a confirmation of the *in vitro* model by RT-qPCR. Values are colored by donor and shown relative to the control condition. On average *CPT1A* expression of 28µg/mL oleic acid exposed cells was upregulated by 19.5 SE 3.00 fold after 48h ($p < 0.0001$), n = 8. **(b)** Bar plot showing the average cell viability and standard error in percent, as determined by Via1-Cassette™ on a NucleoCounter® NC-200™. On average the cell viability of control exposed cells was 93.21 SE 0.43% and of oleic acid exposed cells was 92.89 SE 0.64%. Thus, The solvent control had no effect on $CD4^+$ T cell viability, as expected, at 48h. Thus, there was no effect on $CD4^+$ T cell viability after 48h exposure, n = 8. **(c)** Dot plot showing the average cell diameter and standard error in µm, as determined by Via1-Cassette™ on a NucleoCounter® NC-200™. On average the cell diameter of control exposed cells was 8.99 SE 0.04µm and of oleic acid exposed was 8.98 SE 0.03µm. Thus, there was no effect on $CD4^+$ T cell diameter after 48h exposure, n = 8. **(d)** Bar plot showing the average cell viability and standard error in percent, as determined by Via1-Cassette™ on a NucleoCounter® NC-200™. Left plot shows the cell viability for non-activated cells and right plot shows the cell viability for activated cells. On average, the cell viability of control pre-exposed non-activated cells was 95.45 SE 2.19% and for 28µg/mL oleic acid pre-exposed non-activated cells was 95.91 SE 1.95% after 72h. The cell viability of control pre-exposed activated cells was 79.13 SE 2.19% and for 28µg/mL oleic acid pre-exposed activated cells was 81.51 SE 1.95% after 72h activation with CD3-CD28 beads. Thus, there was no effect on $CD4^+$ T cell viability between the different pre-exposures. However, activation did affect $CD4^+$ T cell viability, where the activated cells were less viable than the not activated cells, n = 8. **(e)** Dot plot showing the average cell diameter and standard error in µm, as determined by Via1-Cassette™ on a NucleoCounter® NC-200™. Left plot shows the cell diameter for non-activated cells and right plot shows the cell diameter for activated cells. On average, the cell diameter of control pre-exposed non-activated cells was 8.86 SE 0.03 µm and for 28µg/mL oleic acid pre-exposed non-activated cells was 8.86 SE 0.03µm after 72h. The cell diameter of control pre-exposed activated cells was 11.04 SE 0.09µm and for 28µg/mL oleic acid pre-exposed activated cells was 11.10 SE 0.10µm after 72h activation with CD3-CD28 beads. Thus, there was no effect on $CD4^+$ T cell diameter between the different pre-exposures. However, activation did affect $CD4^+$ T cell diameter, where the activated cells were larger than the not activated cells, n = 8.

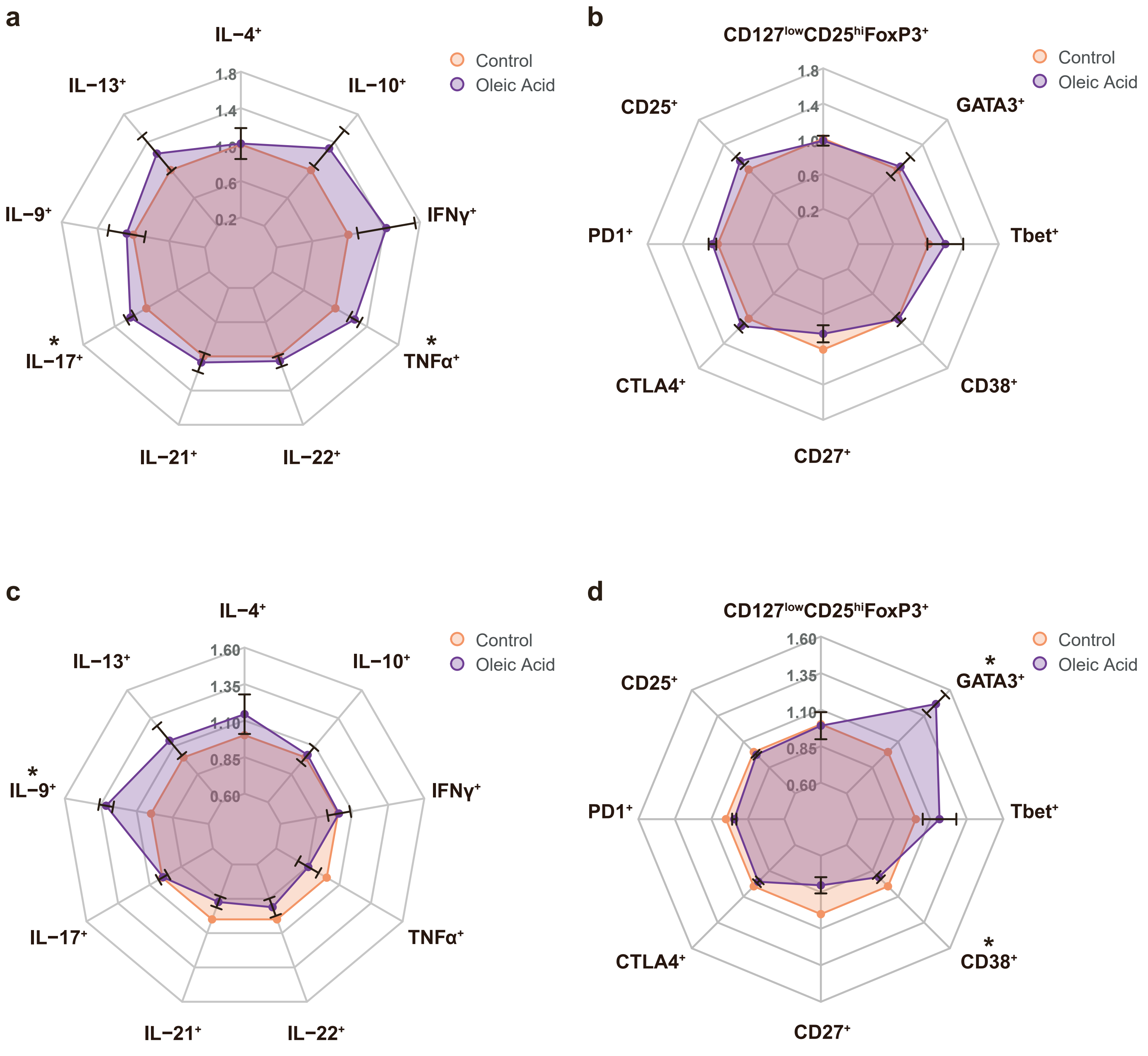

a
IL−4⁺
IL−10⁺
IFNγ⁺
TNFα⁺
IL−22⁺
IL−21⁺
IL−17⁺
IL−9⁺
IL−13⁺
*
*
Control
Oleic Acid
1.8
1.4
1.0
0.6
0.2
b
CD127lowCD25hiFoxP3⁺
GATA3⁺
Tbet⁺
CD38⁺
CD27⁺
CTLA4⁺
PD1⁺
CD25⁺
Control
Oleic Acid
1.8
1.4
1.0
0.6
0.2
c
IL−4⁺
IL−10⁺
IFNγ⁺
TNFα⁺
IL−22⁺
IL−21⁺
IL−17⁺
IL−9⁺
IL−13⁺
*
Control
Oleic Acid
1.60
1.35
1.10
0.85
0.60
d
CD127lowCD25hiFoxP3⁺
GATA3⁺
Tbet⁺
CD38⁺
CD27⁺
CTLA4⁺
PD1⁺
CD25⁺
*
*
Control
Oleic Acid
1.60
1.35
1.10
0.85
0.60

Supplemental Figure 13: Oleic acid pre-exposure leads to changes in expression of extracellular markers, transcription factors, and intracellular cytokines. (*) $P_{FDR} < 0.05$, n = 8, related to Figure 3. **(a)** Radar plot of various $CD4^+$ T cell internal cytokines expressed in $CD4^+$ T cells after 48h of oleic acid exposure or control followed by 72h of rest and 4h stimulus with PMA/ionomycin. Values are expressed as fold change and standard error relative to control. **(b)** Radar plot of various $CD4^+$ T cell external markers and transcription factors expressed in $CD4^+$ T cells after 48h of oleic acid exposure or control followed by 72h of rest and 4h stimulus with PMA/ionomycin. Values are expressed as fold change and standard error relative to control. **(c)** Radar plot of various $CD4^+$ T cell internal cytokines expressed in $CD4^+$ T cells after 48h of oleic acid exposure or control followed by 72h of activation with CD3/CD28 activation beads and 4h additional stimulus with PMA/ionomycin. Values are expressed as fold change and standard error relative to control. **(d)** Radar plot of various $CD4^+$ T cell external markers and transcription factors expressed in $CD4^+$ T cells after 48h of oleic acid exposure or control followed by 72h of activation with CD3/CD28 activation beads and 4h additional stimulus with PMA/ionomycin. Values are expressed as fold change and standard error relative to control.

Data S14.1: Gating strategy for the second spectral cytometry analysis of non-activated CD4$^+$ T cells pre-exposed to oleic acid

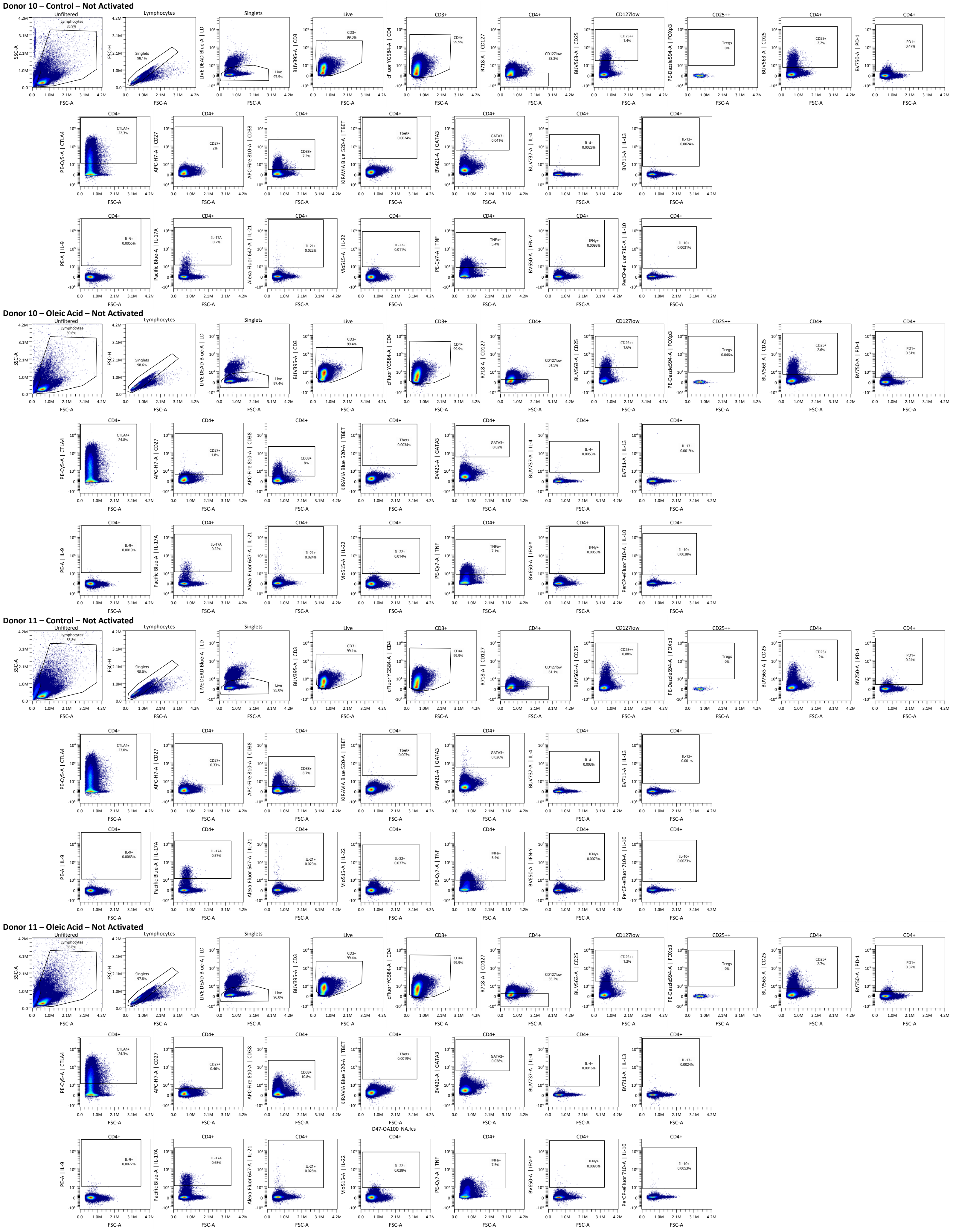

Data S14.2: Gating strategy for the second spectral cytometry analysis of non-activated CD4⁺ T cells pre-exposed to oleic acid

Donor 12 – Control – Not Activated

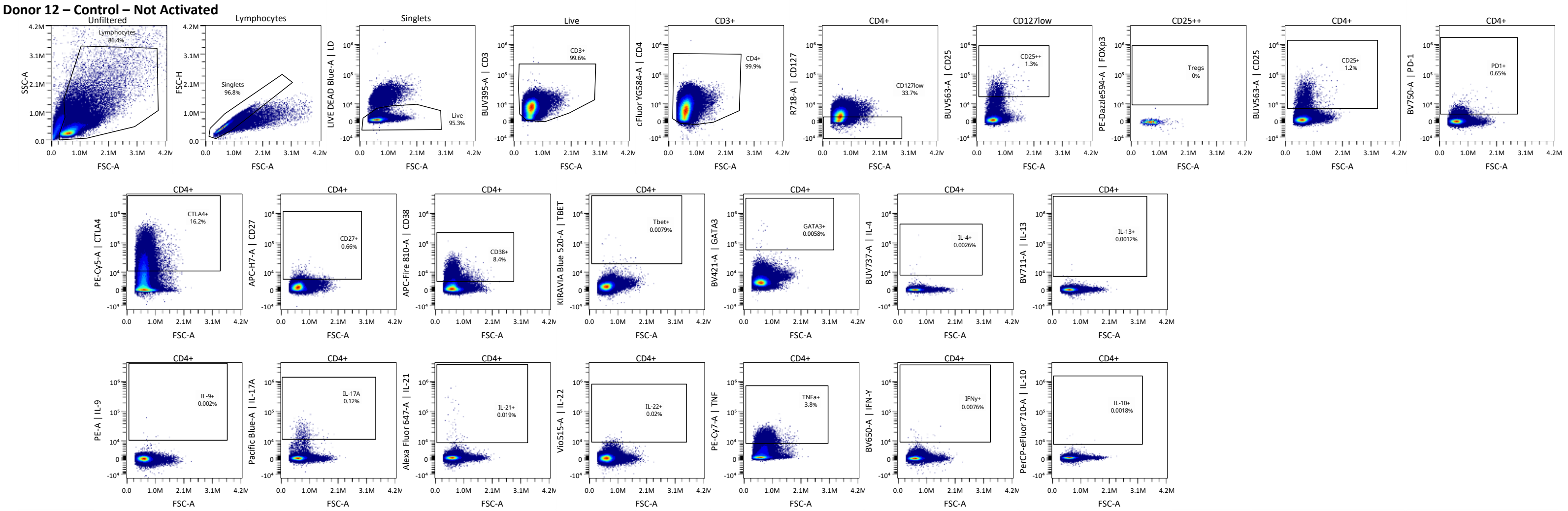

Donor 12 – Oleic Acid – Not Activated

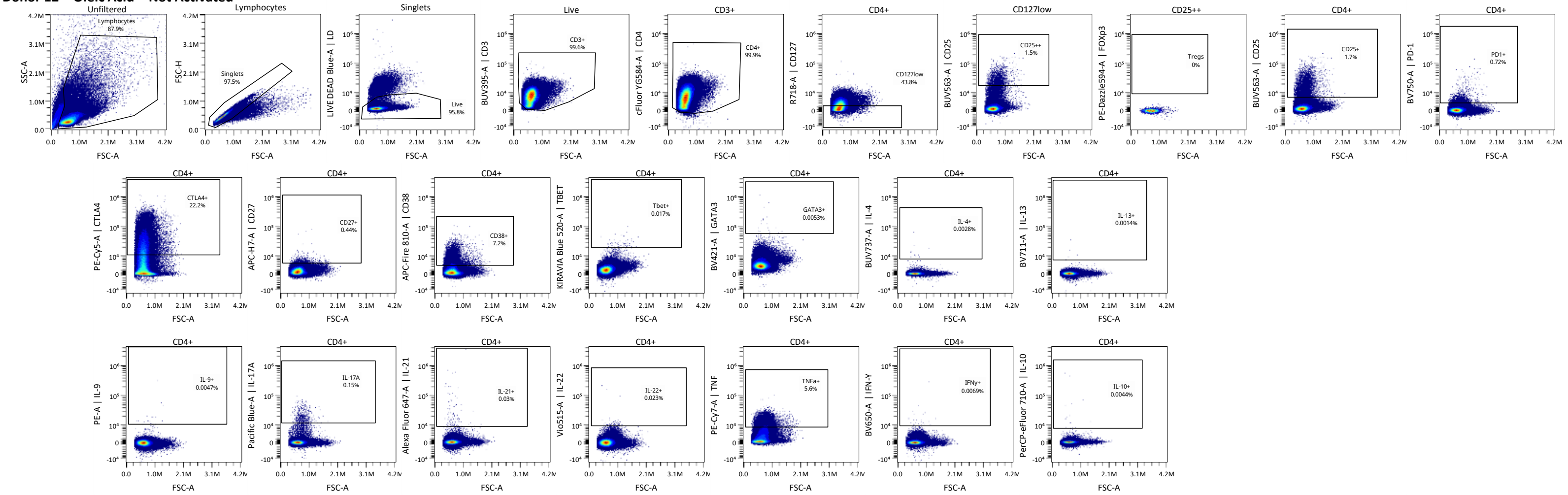

Donor 13 – Control – Not Activated

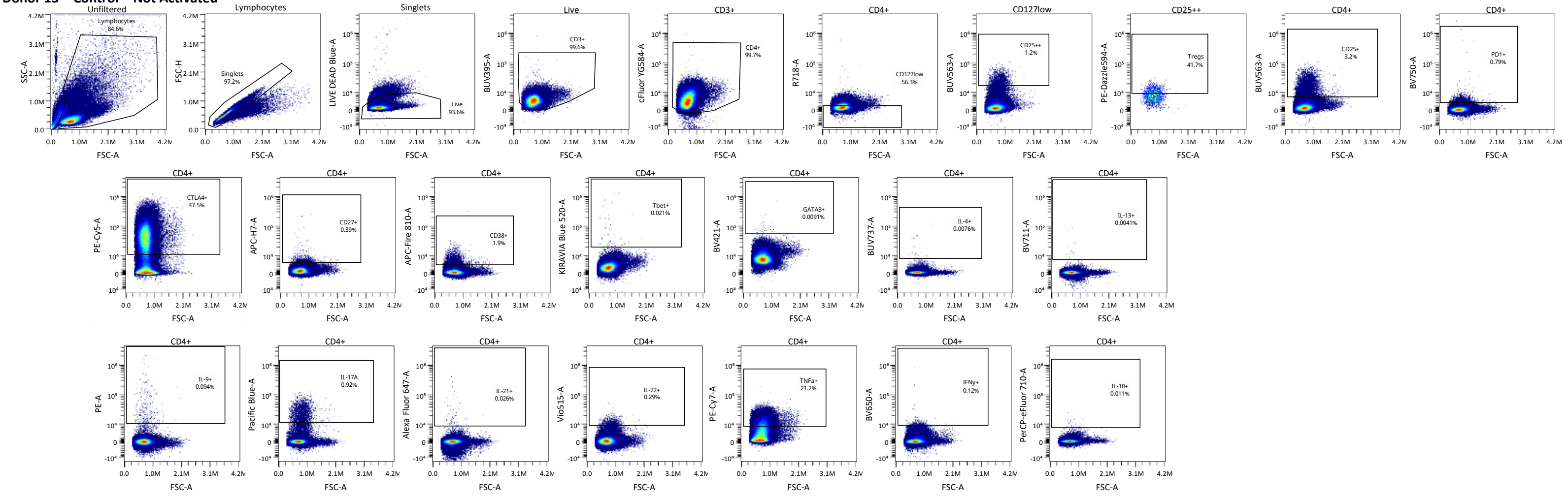

Donor 13 – Oleic Acid – Not Activated

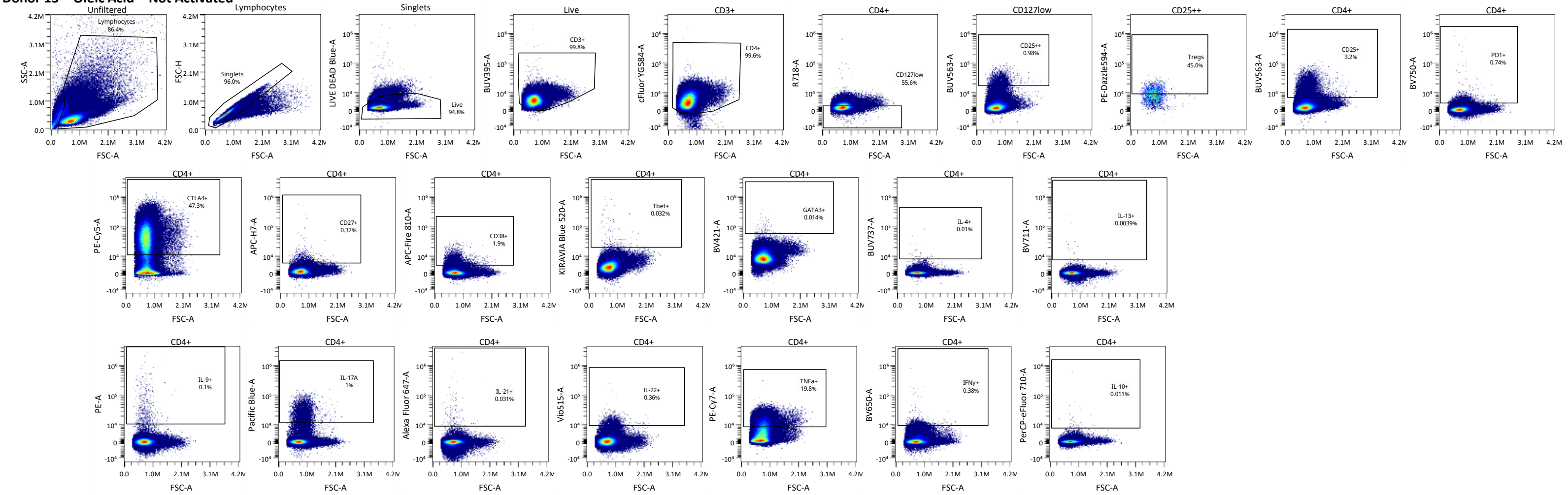

Data S14.3: Gating strategy for the second spectral cytometry analysis of non-activated $CD4^{+}$ T cells pre-exposed to oleic acid

Donor 14 – Control – Not Activated

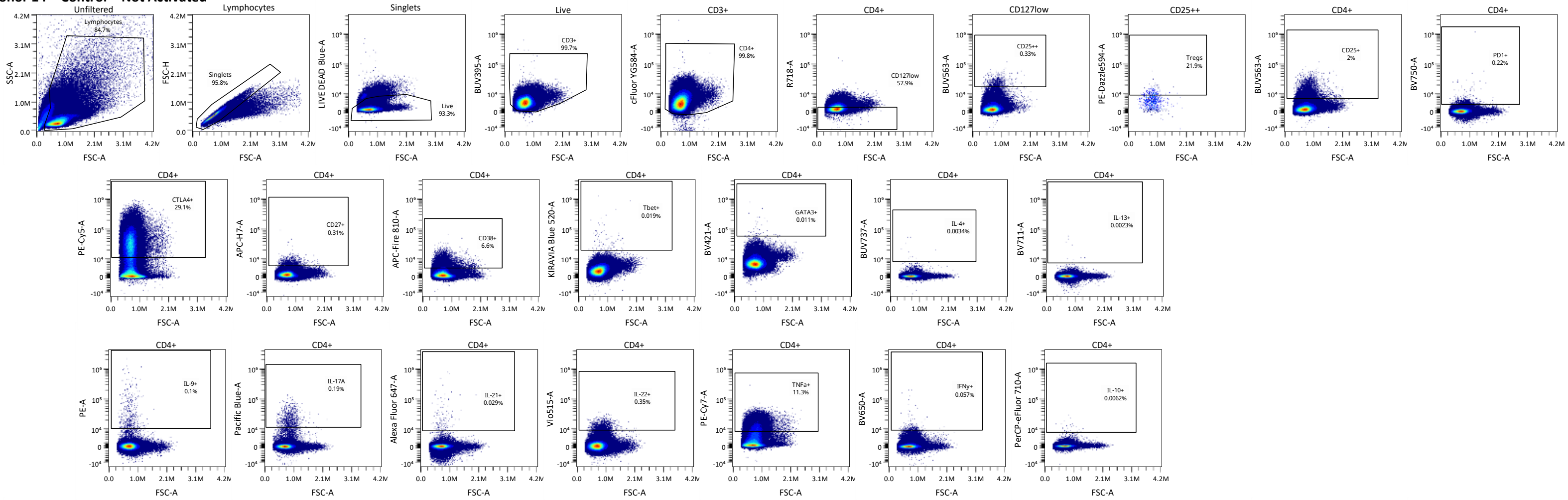

Donor 14 – Oleic Acid – Not Activated

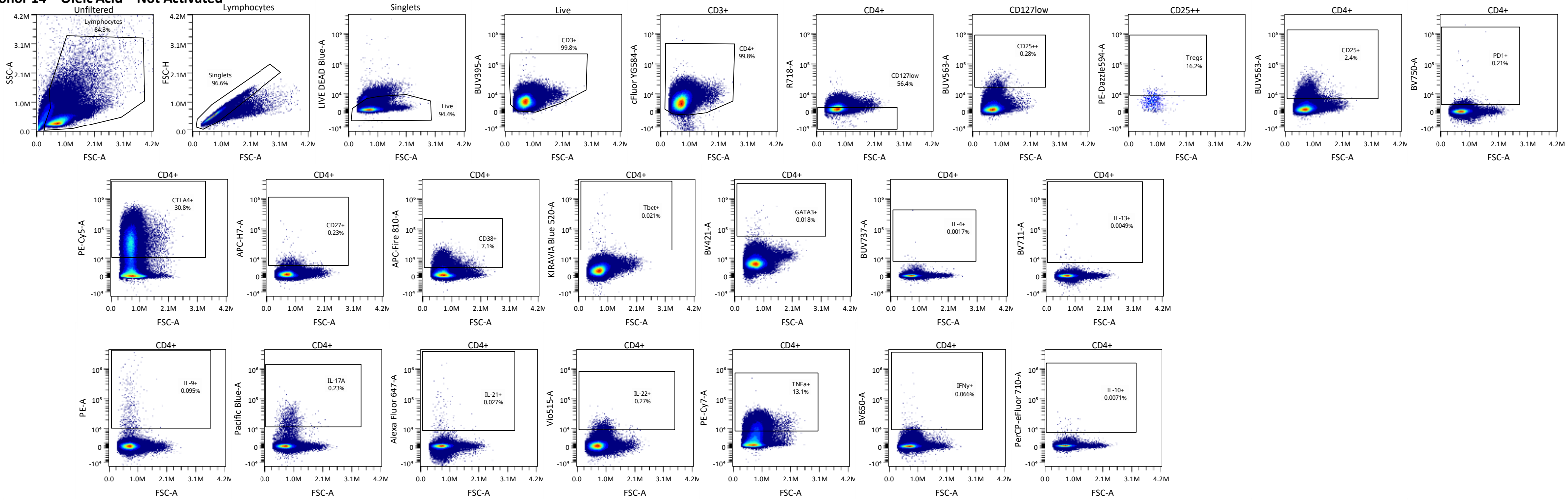

Donor 15 – Control – Not Activated

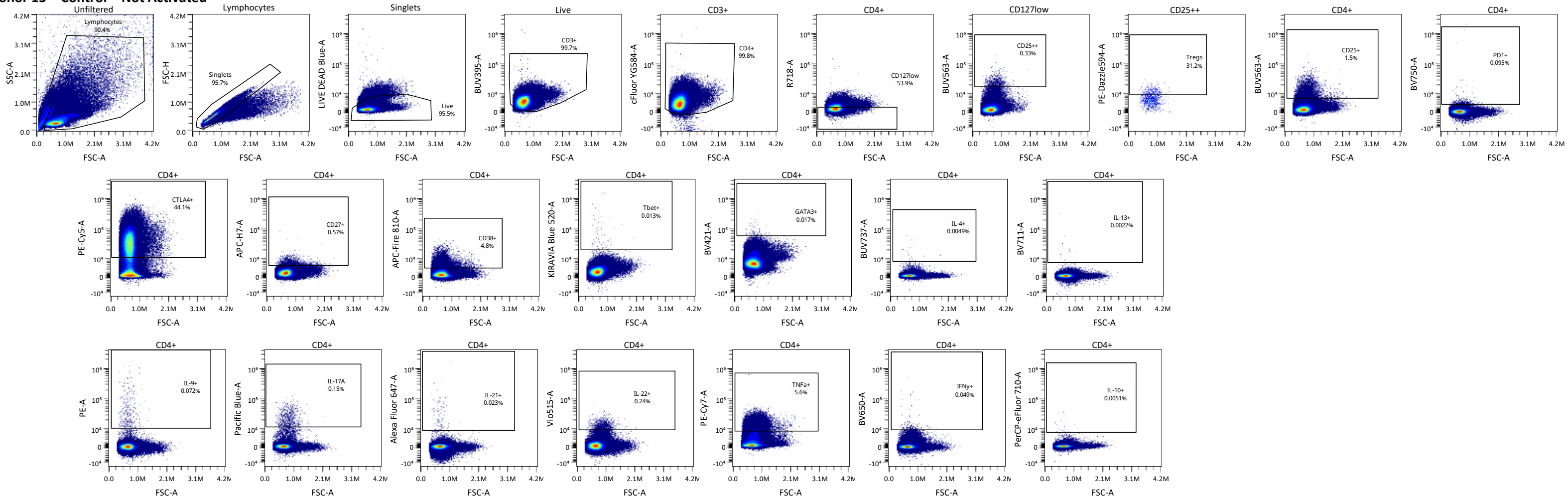

Donor 15 – Oleic Acid – Not Activated

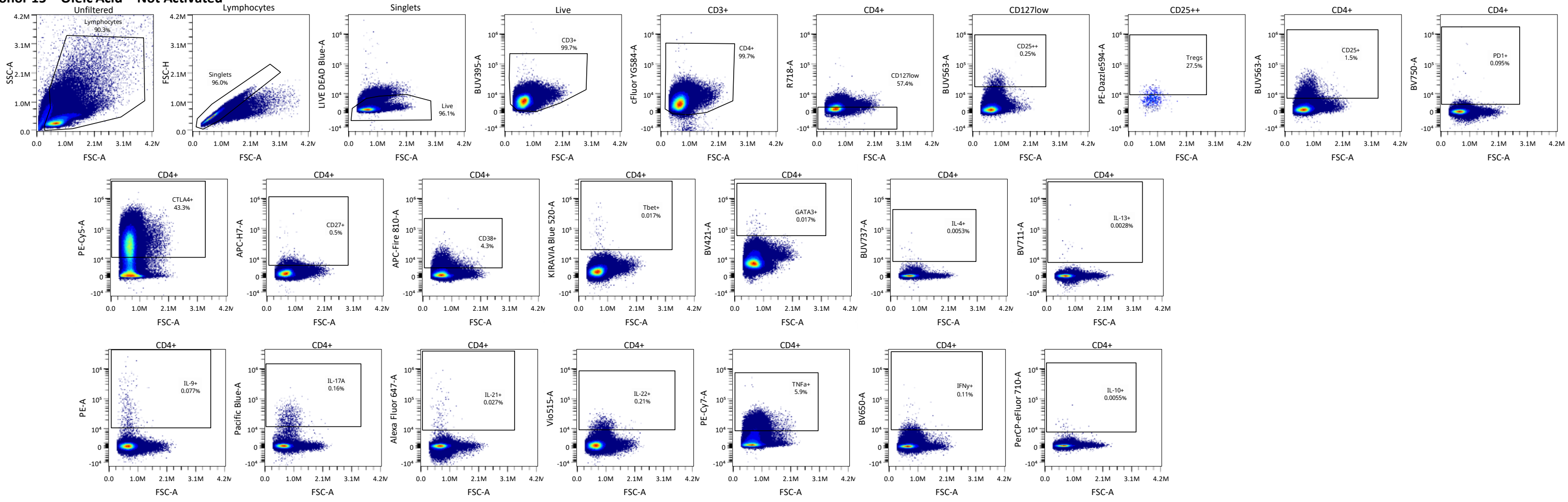

Data S14.4: Gating strategy for the second spectral cytometry analysis of non-activated CD4^{+} T cells pre-exposed to oleic acid

Donor 16 – Control – Not Activated

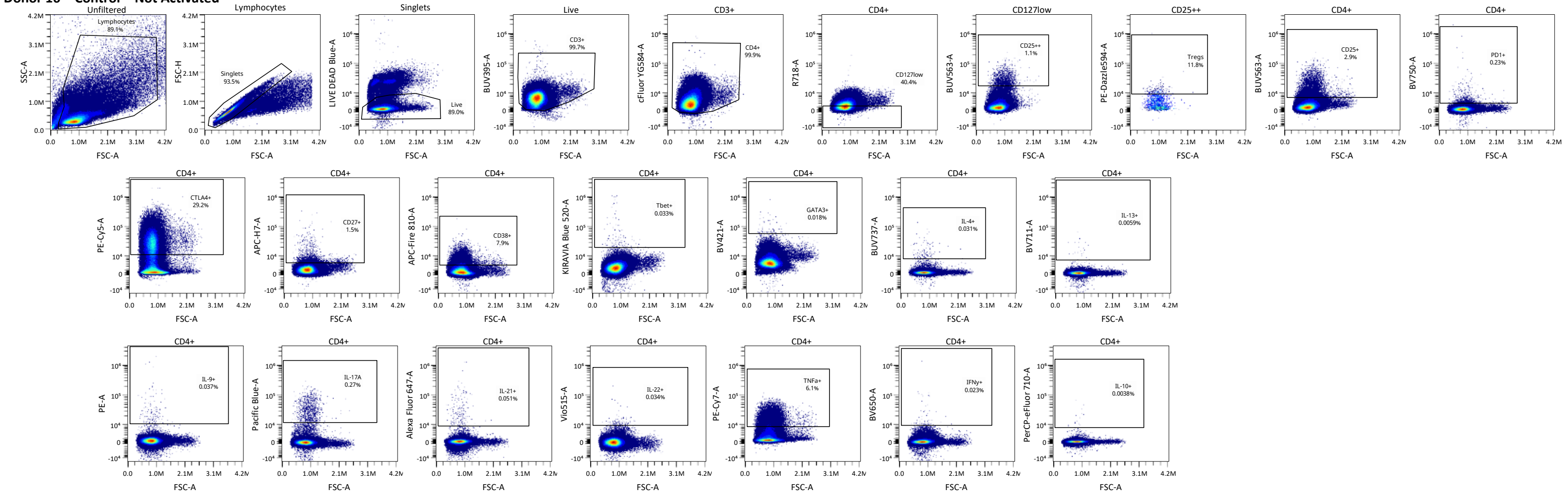

Donor 16 – Oleic Acid – Not Activated

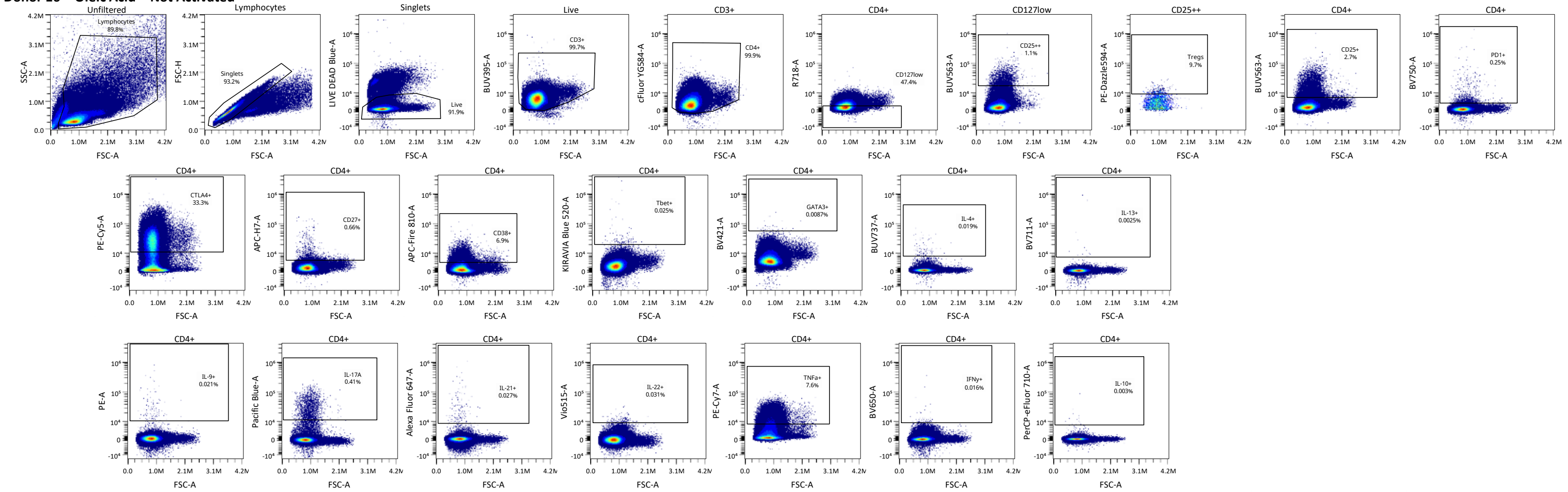

Donor 17 – Control – Not Activated

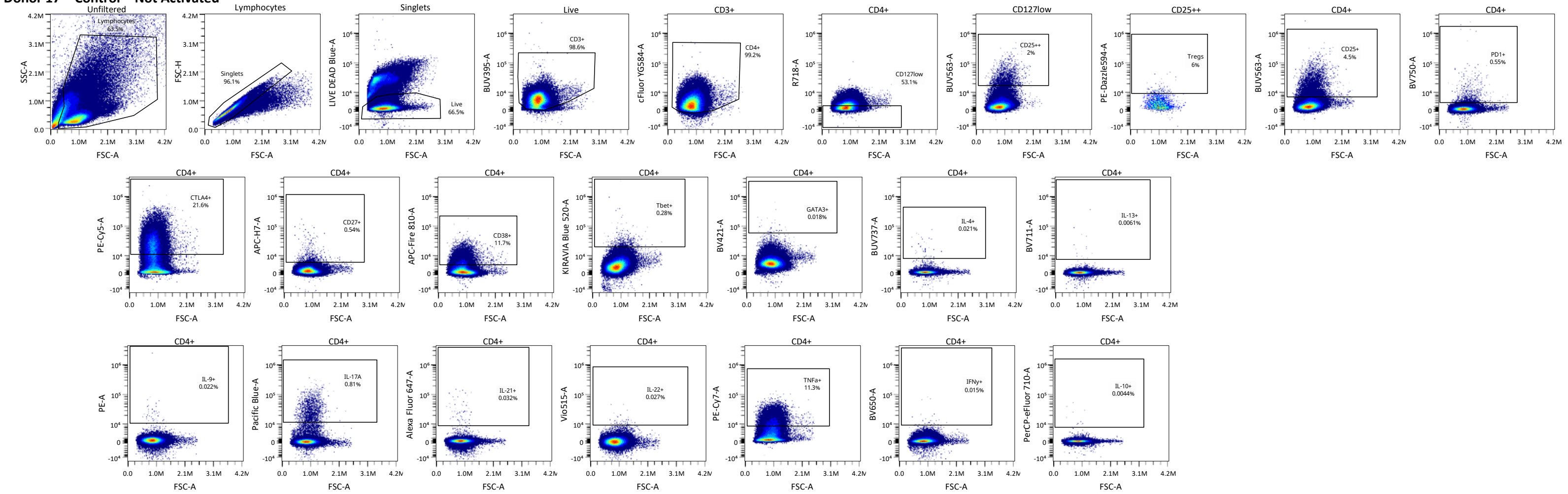

Donor 17 – Oleic Acid – Not Activated

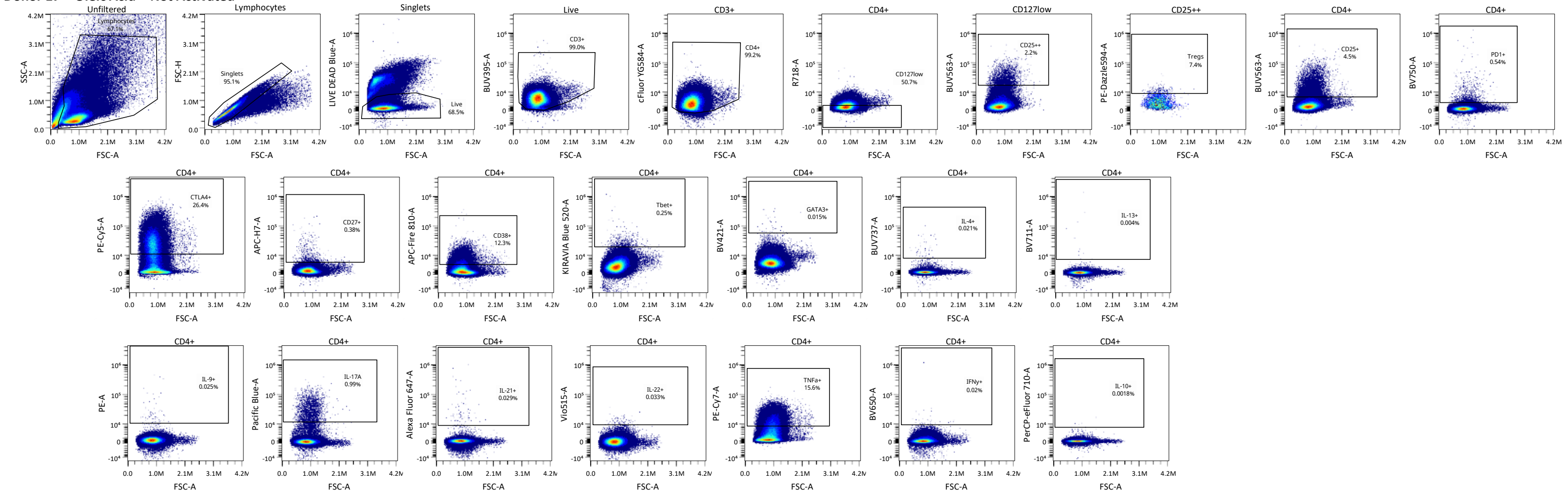

Supplemental Figure 14: Gating strategy for the second spectral cytometry analysis of non-activated $CD4^+$ T cells pre-exposed to oleic acid from 8 independent donors, n = 8, related to Figure 3. Gating strategy is the same for all 8 independent donors analyzed and includes gates set for all markers measured in the panel.

Data S15.1: Gating strategy for the second spectral cytometry analysis of activated CD4$^+$ T cells pre-exposed to oleic acid

Donor 10 – Control – Activated

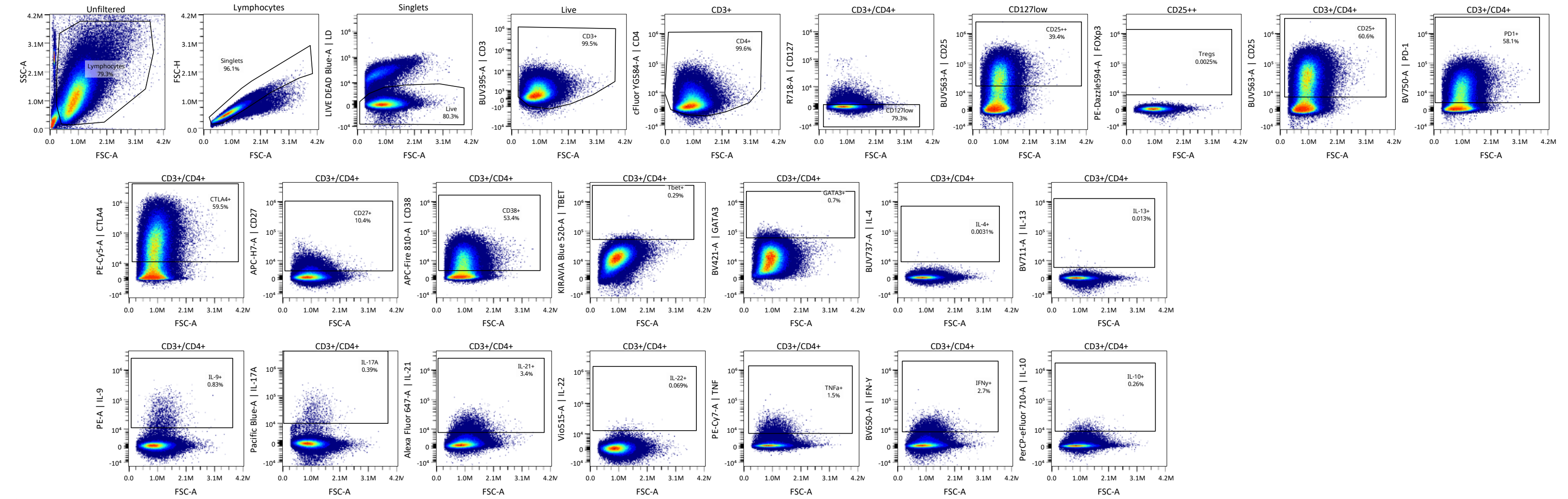

Donor 10 – Oleic Acid – Activated

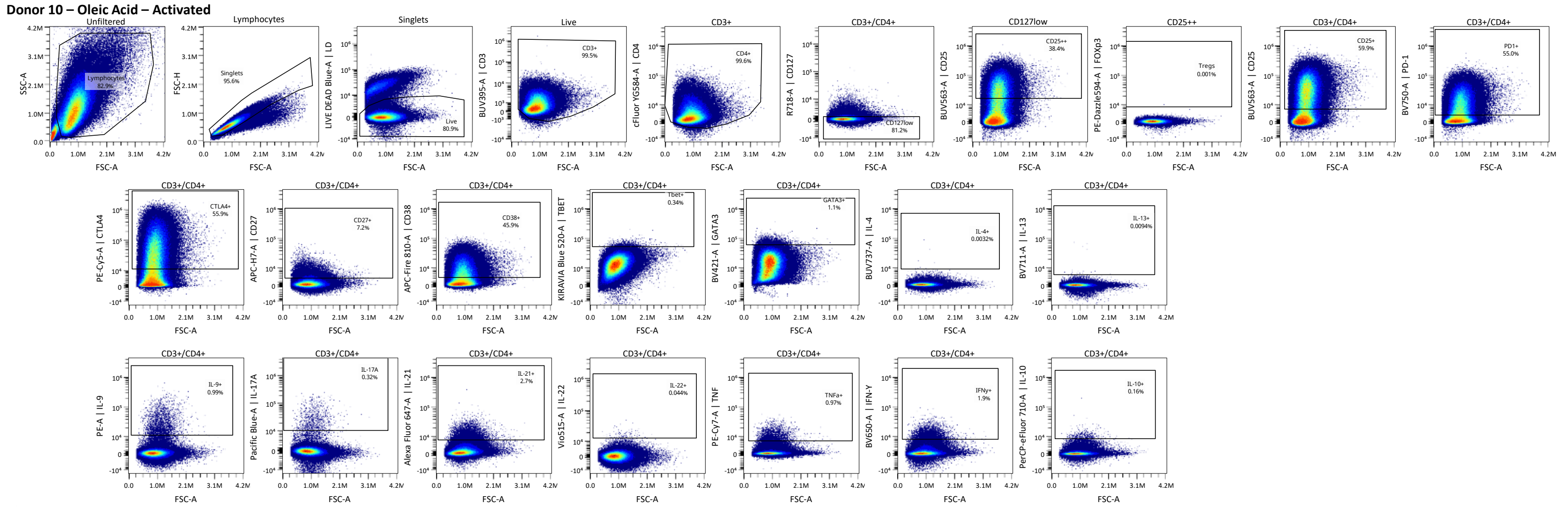

Donor 11 – Control – Activated

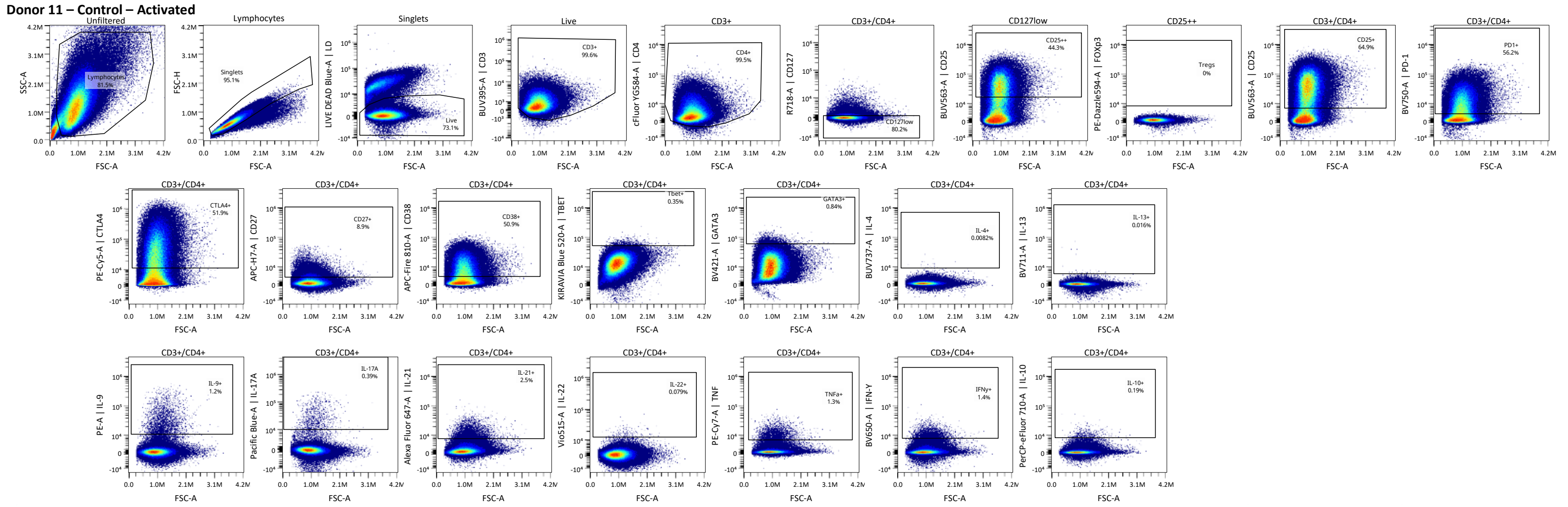

Donor 11 – Oleic Acid – Activated

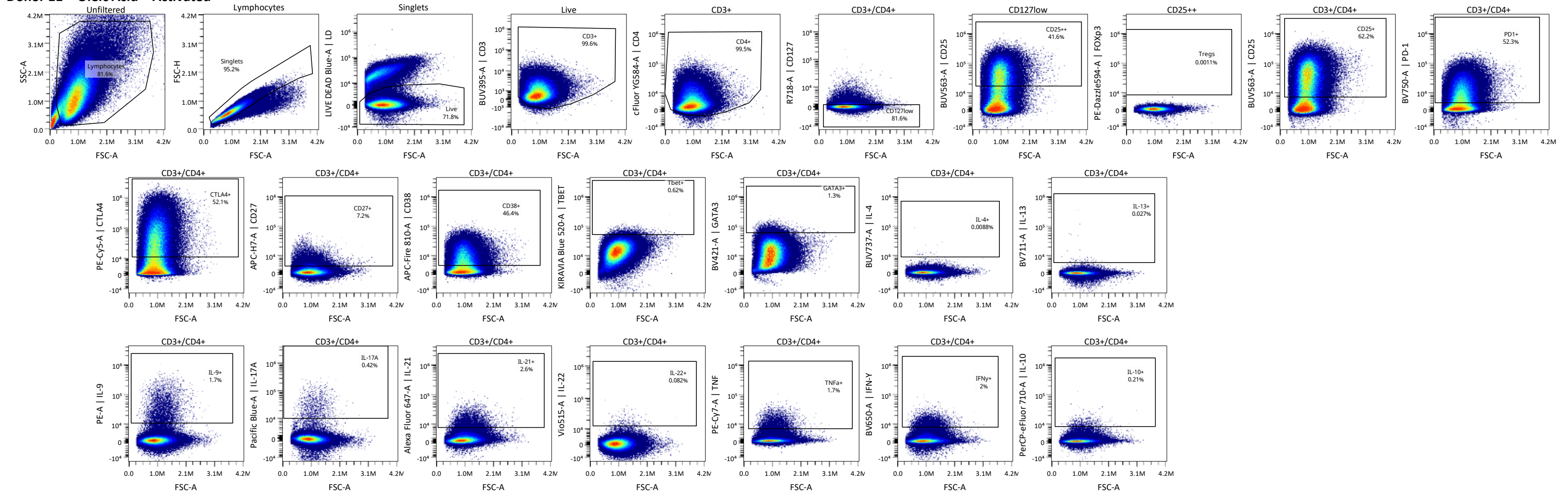

Data S15.2: Gating strategy for the second spectral cytometry analysis of activated CD4$^+$ T cells pre-exposed to oleic acid

Donor 12 – Control – Activated

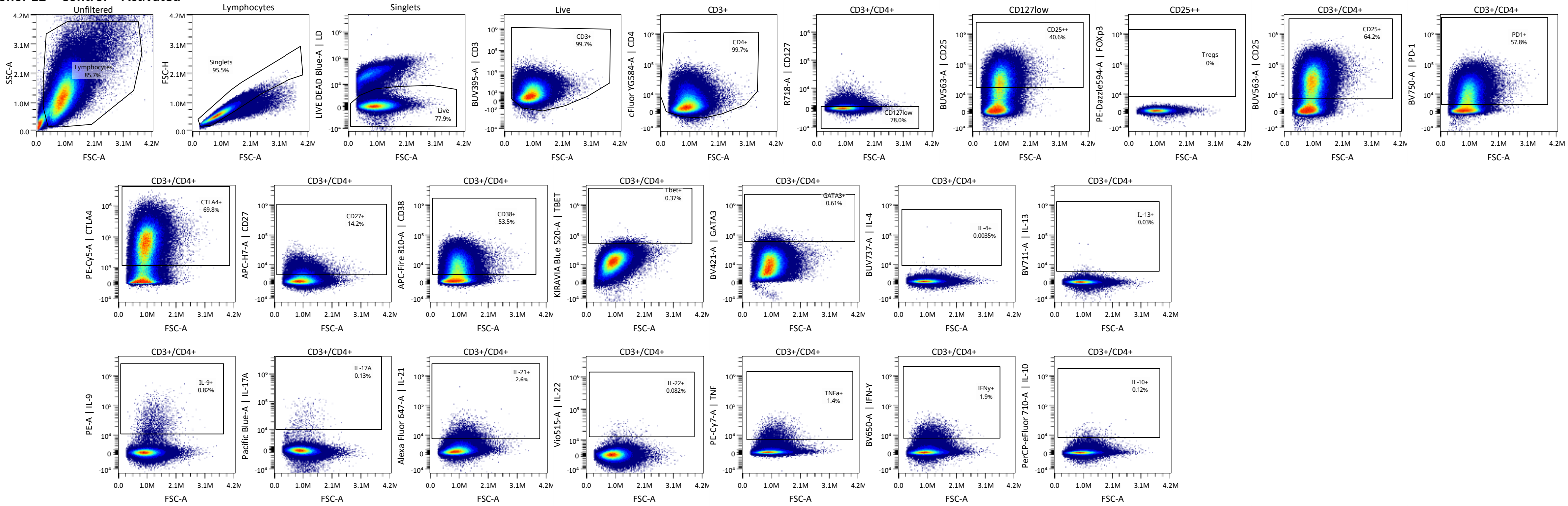

Donor 12 – Oleic Acid – Activated

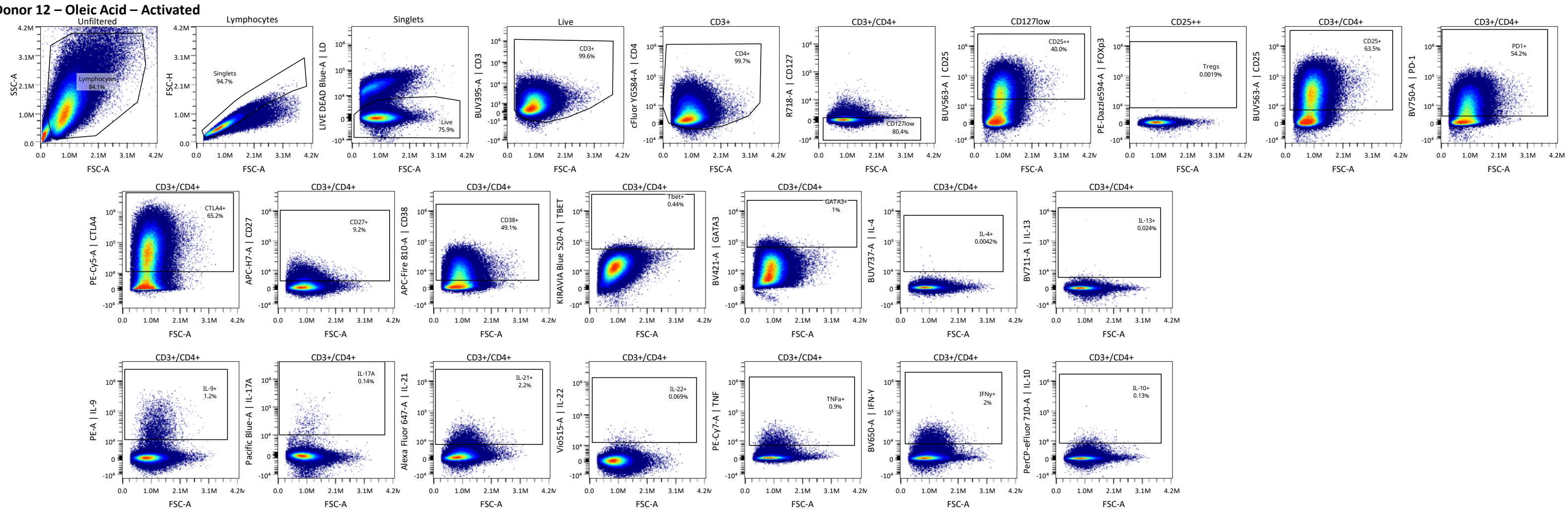

Donor 13 – Control – Activated

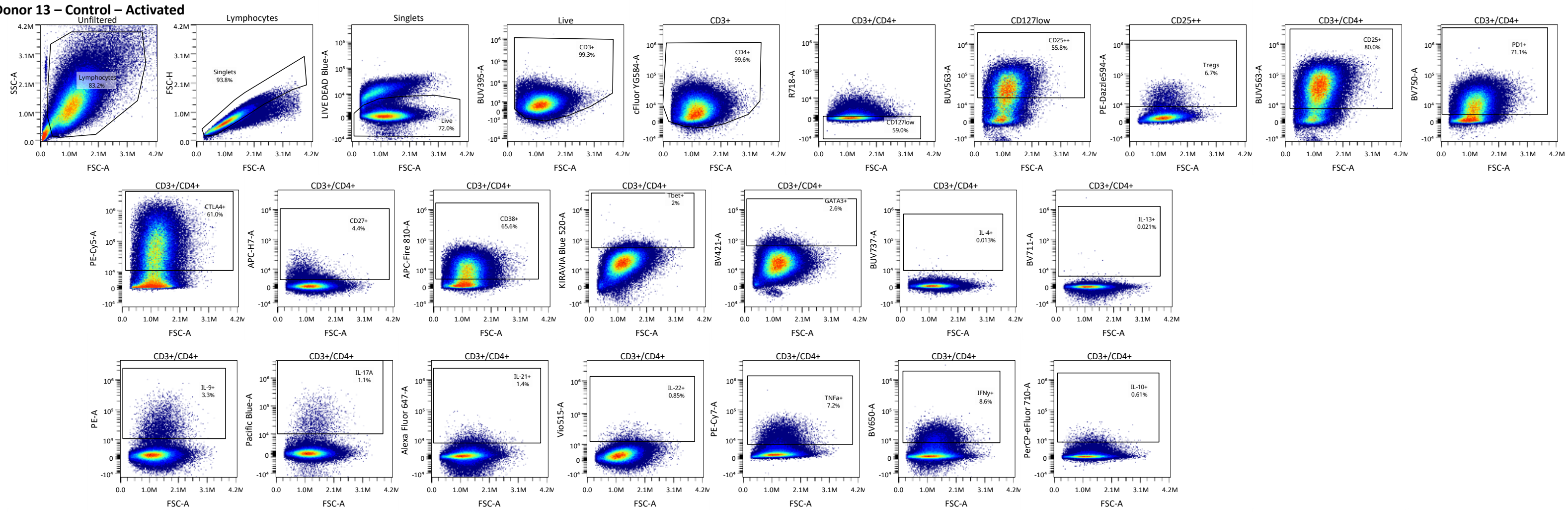

Donor 13 – Oleic Acid – Activated

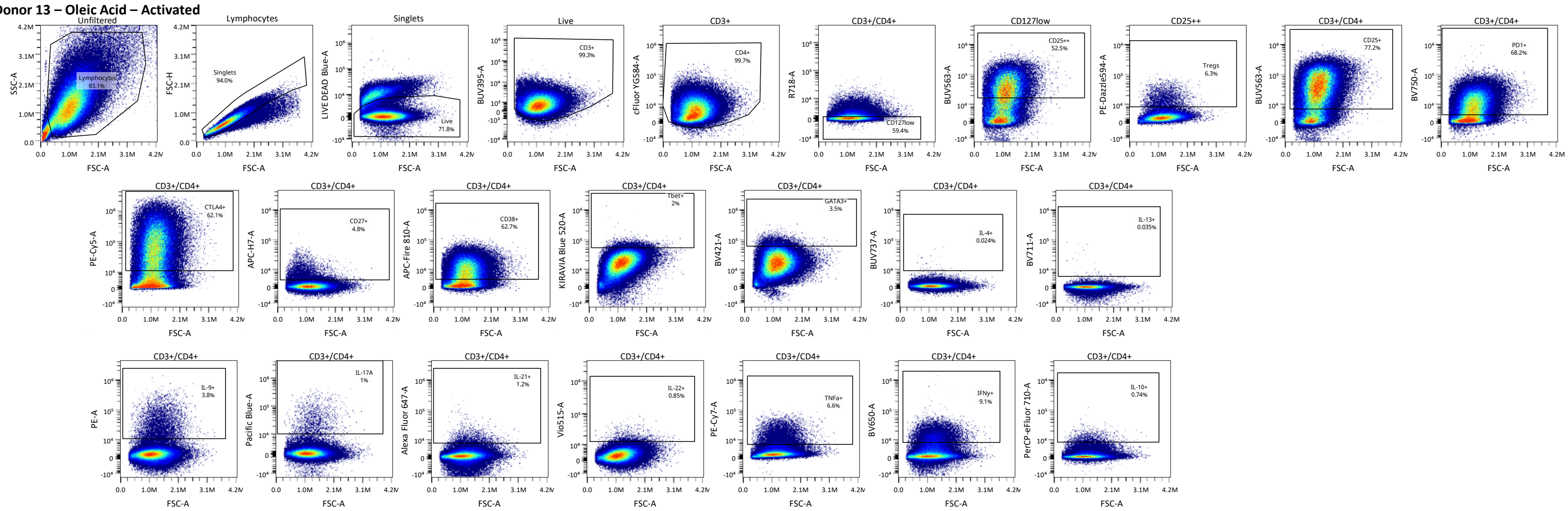

Data S15.3: Gating strategy for the second spectral cytometry analysis of activated CD4^{+} T cells pre-exposed to oleic acid

Donor 14 – Control – Activated

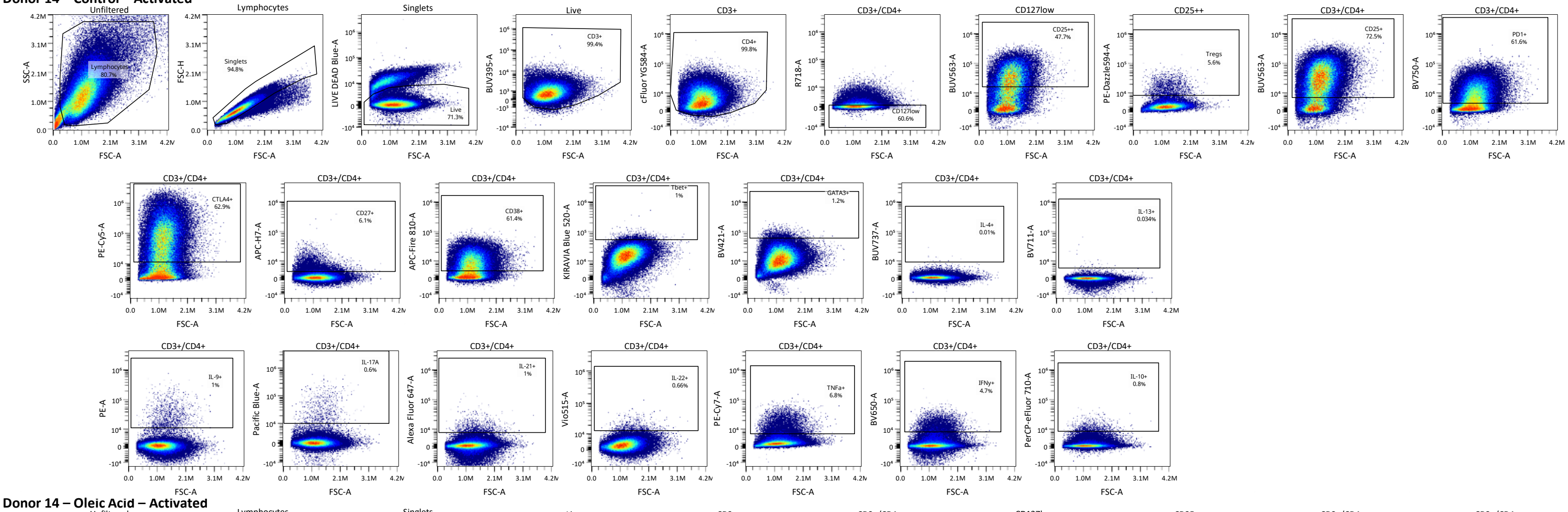

Donor 14 – Oleic Acid – Activated

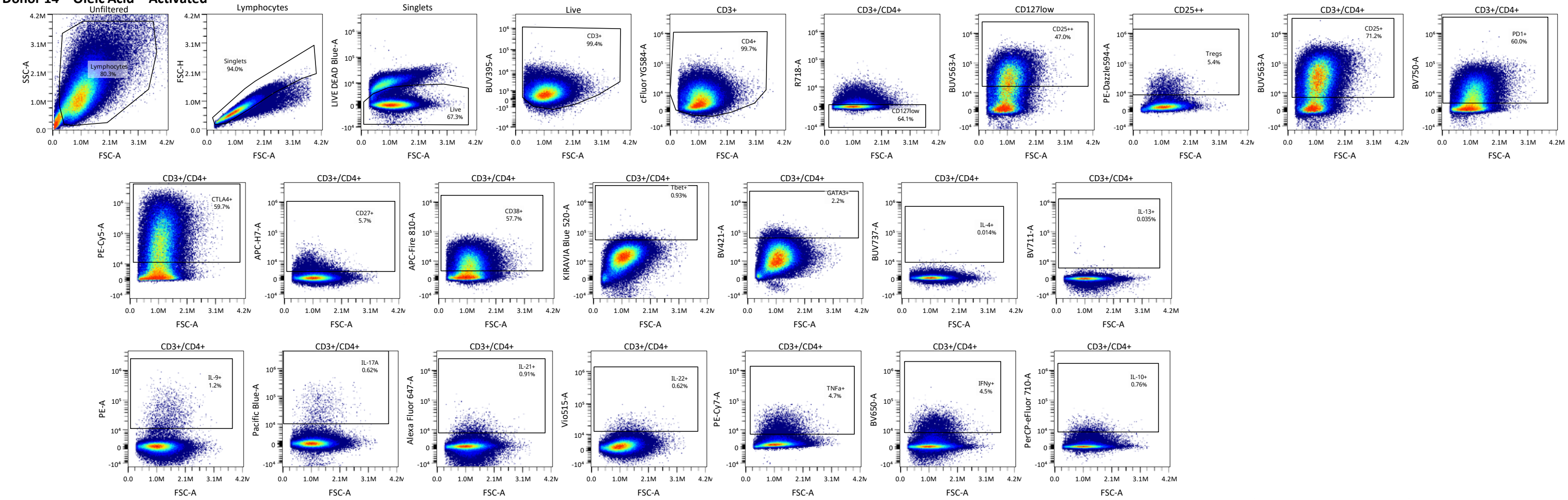

Donor 15 – Control – Activated

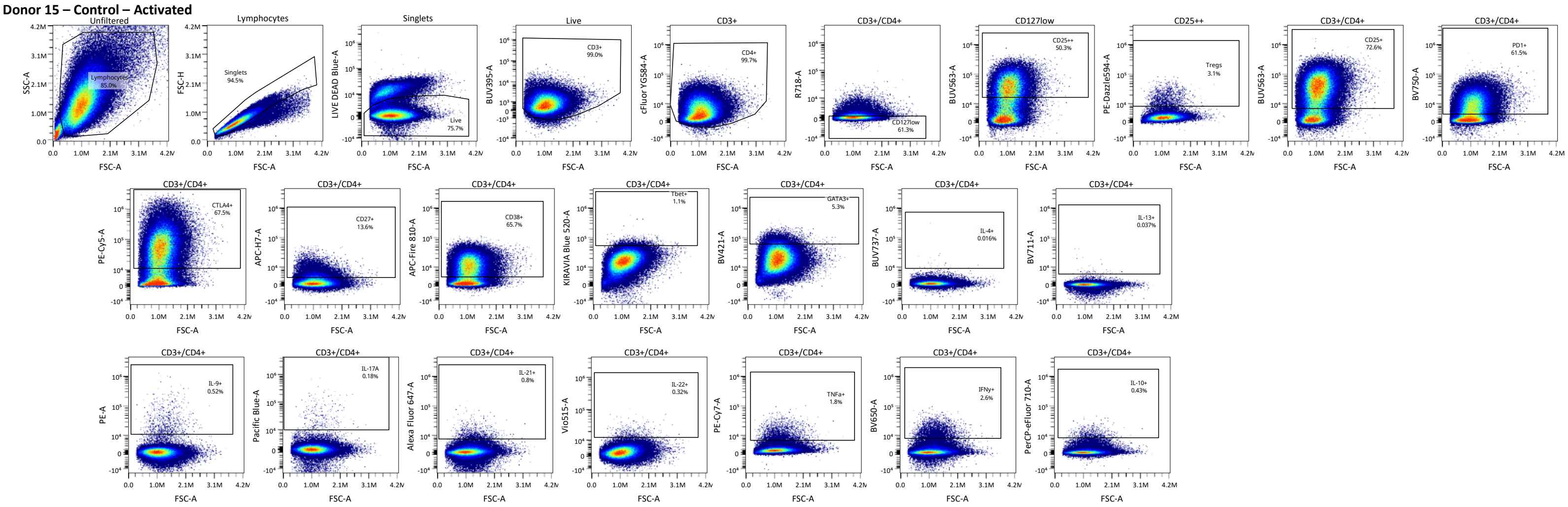

Donor 15 – Oleic Acid – Activated

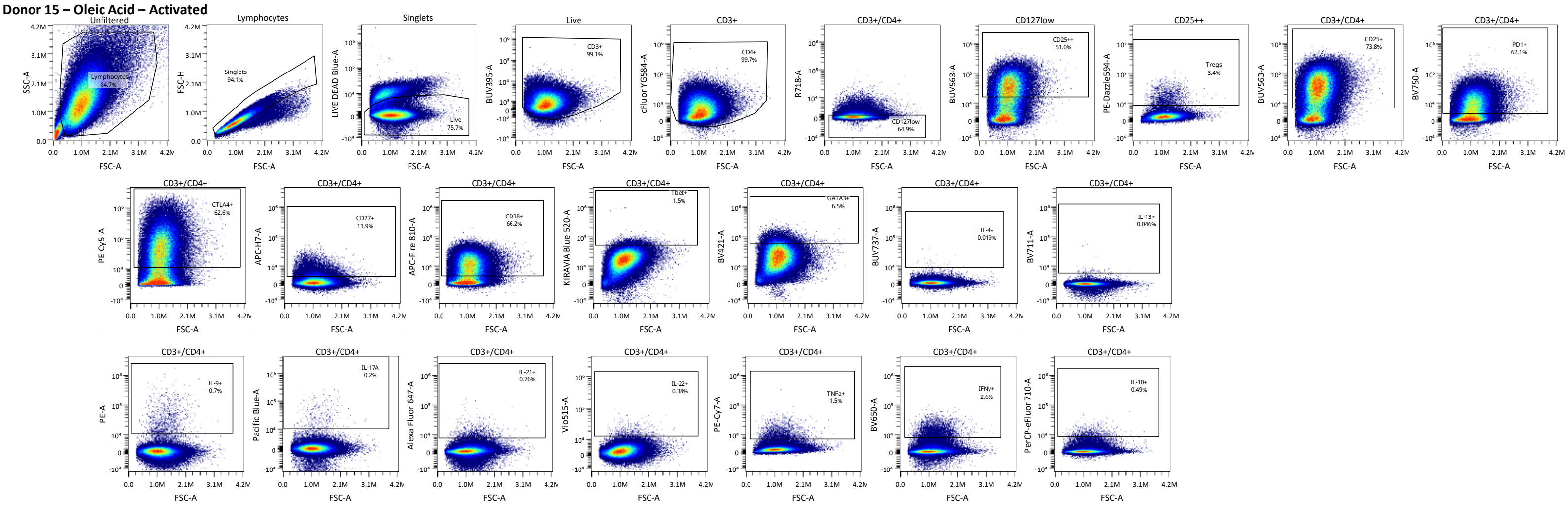

Data S15.4: Gating strategy for the second spectral cytometry analysis of activated CD4⁺ T cells pre-exposed to oleic acid

Donor 16 – Control – Activated

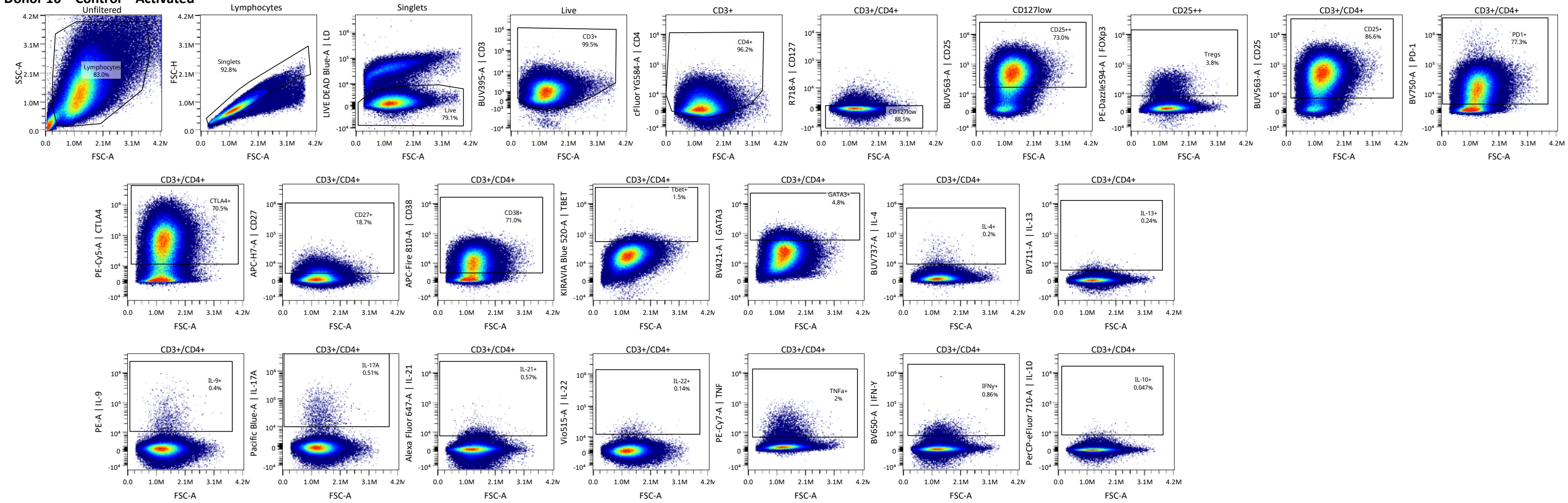

Donor 16 – Oleic Acid – Activated

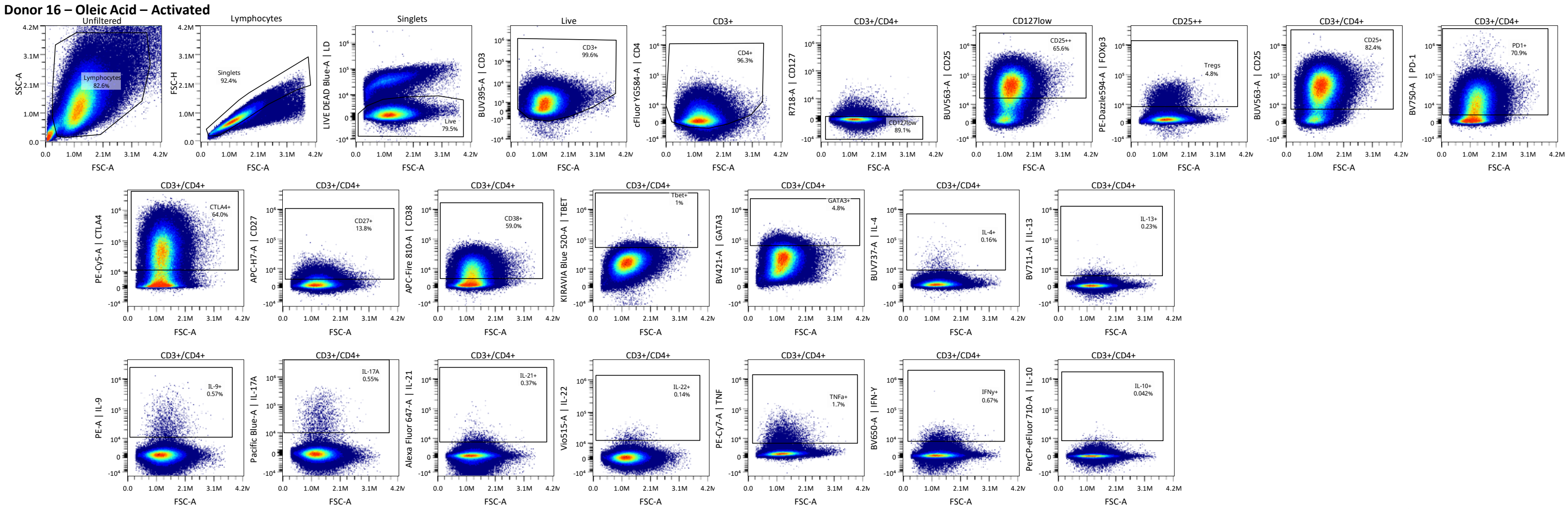

Donor 17 – Control – Activated

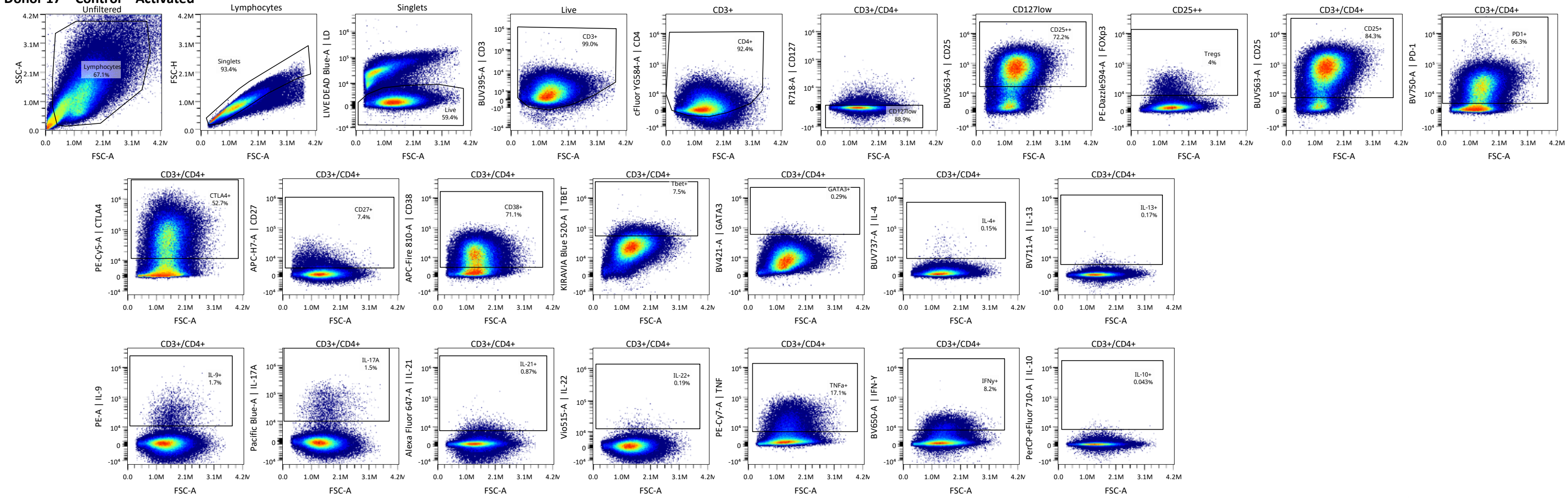

Donor 17 – Oleic Acid – Activated

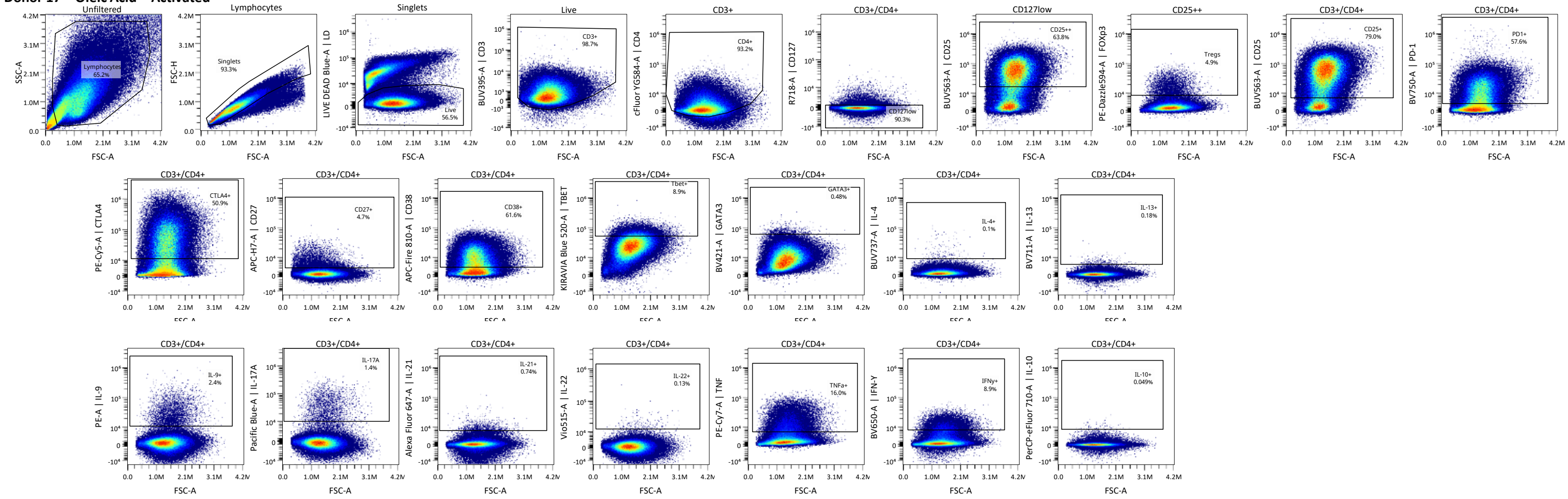

Supplemental Figure 15: Gating strategy for the second spectral cytometry analysis of activated CD4$^+$ T cells pre-exposed to oleic acid from 8 independent donors, n = 8, related to Figure 3. Gating strategy is the same for all 8 independent donors analyzed and includes gates set for all markers measured in the panel.

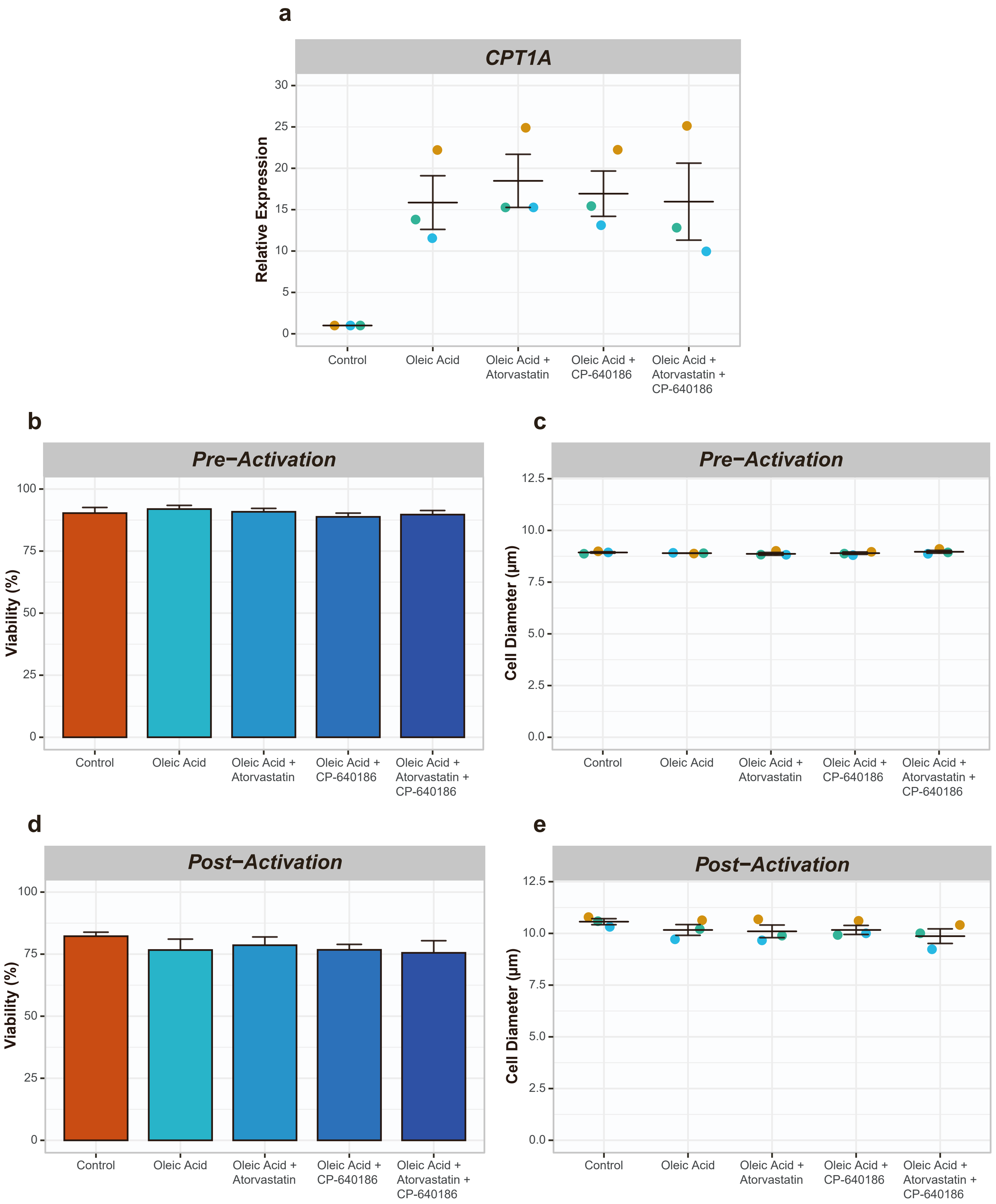
a
CPT1A
Relative Expression
0
5
10
15
20
25
30
Control
Oleic Acid
Oleic Acid + Atorvastatin
Oleic Acid + CP-640186
Oleic Acid + Atorvastatin + CP-640186
b
Pre−Activation
Viability (%)
0
25
50
75
100
Control
Oleic Acid
Oleic Acid + Atorvastatin
Oleic Acid + CP-640186
Oleic Acid + Atorvastatin + CP-640186
c
Pre−Activation
Cell Diameter (µm)
0.0
2.5
5.0
7.5
10.0
12.5
Control
Oleic Acid
Oleic Acid + Atorvastatin
Oleic Acid + CP-640186
Oleic Acid + Atorvastatin + CP-640186
d
Post−Activation
Viability (%)
0
25
50
75
100
Control
Oleic Acid
Oleic Acid + Atorvastatin
Oleic Acid + CP-640186
Oleic Acid + Atorvastatin + CP-640186
e
Post−Activation
Cell Diameter (µm)
0.0
2.5
5.0
7.5
10.0
12.5
Control
Oleic Acid
Oleic Acid + Atorvastatin
Oleic Acid + CP-640186
Oleic Acid + Atorvastatin + CP-640186

Supplemental Figure 16: Verification of viability, cell diameter and *CPT1A* expression post-exposure and post-activation for spectral cytometry with metabolic inhibitors. Related to Figure 4. **(a)** Line plot showing the relative expression of *CPT1A* per donor after 48h of oleic acid exposure, with or without inhibitors added as a confirmation of the *in vitro* model by RT-qPCR. Values are colored by donor and shown relative to the control condition. On average *CPT1A* was upregulated 15.86 SE 3.24 fold when exposed to oleic acid, 18.48 SE 3.21 fold when exposed to oleic acid + atorvastatin, 16.93 SE 2.74 fold when exposed to oleic acid + CP-640186, and 15.97 SE 4.65 fold when exposed to oleic acid + atorvastatin + CP-640186. Atorvastatin is an HMGCR inhibitor, blocking cholesterol biosynthesis, and CP-640186 is an ACC inhibitor, blocking fatty acid biosynthesis. As CPT1A is a part of the fatty acid oxidation pathway there was no influence of the inhibitors on *CPT1A* expression, n = 3. **(b)** Bar plot showing the average cell viability and standard error in percent, as determined by Via1-Cassette™ on a NucleoCounter® NC-200™. On average the cell viability of control exposed cells was 90.30 SE 2.27%, oleic acid exposed cells was 91.93 SE 1.48%, oleic acid + atorvastatin exposed cells was 90.83 SE 1.37%, oleic acid + CP-640186 exposed cells was 88.80 SE 1.50%, and oleic acid + atorvastatin + CP-640186 exposed cells was 89.70 SE 2.86% at 48h. Thus, there was no effect on $CD4^+$ T cell viability after 48h exposure, n = 3. **(c)** Dot plot showing the average cell diameter and standard error in µm, as determined by Via1-Cassette™ on a NucleoCounter® NC-200™. On average the cell diameter of control exposed cells was 8.93 SE 0.03µm, oleic acid exposed cells was 8.90 SE 0.0µm, oleic acid + atorvastatin exposed cells was 8.87 SE 0.07µm, oleic acid + CP-640186 exposed cells was 8.90 SE 0.0µm, and oleic acid + atorvastatin + CP-640186 exposed cells was 8.97 SE 0.07µm after 48h exposure. Thus, there was no effect on $CD4^+$ T cell diameter after 48h exposure, n = 3. **(d)** Bar plot showing the average cell viability and standard error in percent, as determined by Via1-Cassette™ on a NucleoCounter® NC-200™ after 72h activation with CD3-CD28 activation beads. On average the cell viability of control exposed cells was 82.23 SE 1.62%, oleic acid exposed cells was 76.67 SE 7.61%, oleic acid + atorvastatin exposed cells was 78.60 SE 3.33%, oleic acid + CP-640186 exposed cells was 76.73 SE 2.20%, and oleic acid + atorvastatin + CP-640186 exposed cells was 75.53 SE 4.88% after 72h activation. Thus, there was no effect on $CD4^+$ T cell viability after 72h activation between the different conditions. However, the cells were slightly less viable after activation than before activation, n = 3. **(e)** Dot plot showing the average cell diameter and standard error in µm, as determined by Via1-Cassette™ on a NucleoCounter® NC-200™. On average the cell diameter of control exposed cells was 10.57 SE 0.15µm, oleic acid exposed cells was 10.17 SE 0.26µm, oleic acid + atorvastatin exposed cells was 10.10 SE 0.31µm, oleic acid + CP-640186 exposed cells was 10.17 SE 0.22µm, and oleic acid + atorvastatin + CP-640186 exposed cells was 9.87 SE 0.61µm after 72h activation. Thus, there was no effect on $CD4^+$ T cell diameter after 48h exposure. However, the cells were larger after activation than before activation as was expected, n = 3.

Data S17.1: Gating strategy for the inhibitor spectral cytometry analysis of activated CD4^{+} T cells pre-exposed to oleic acid with or without metabolic inhibitors

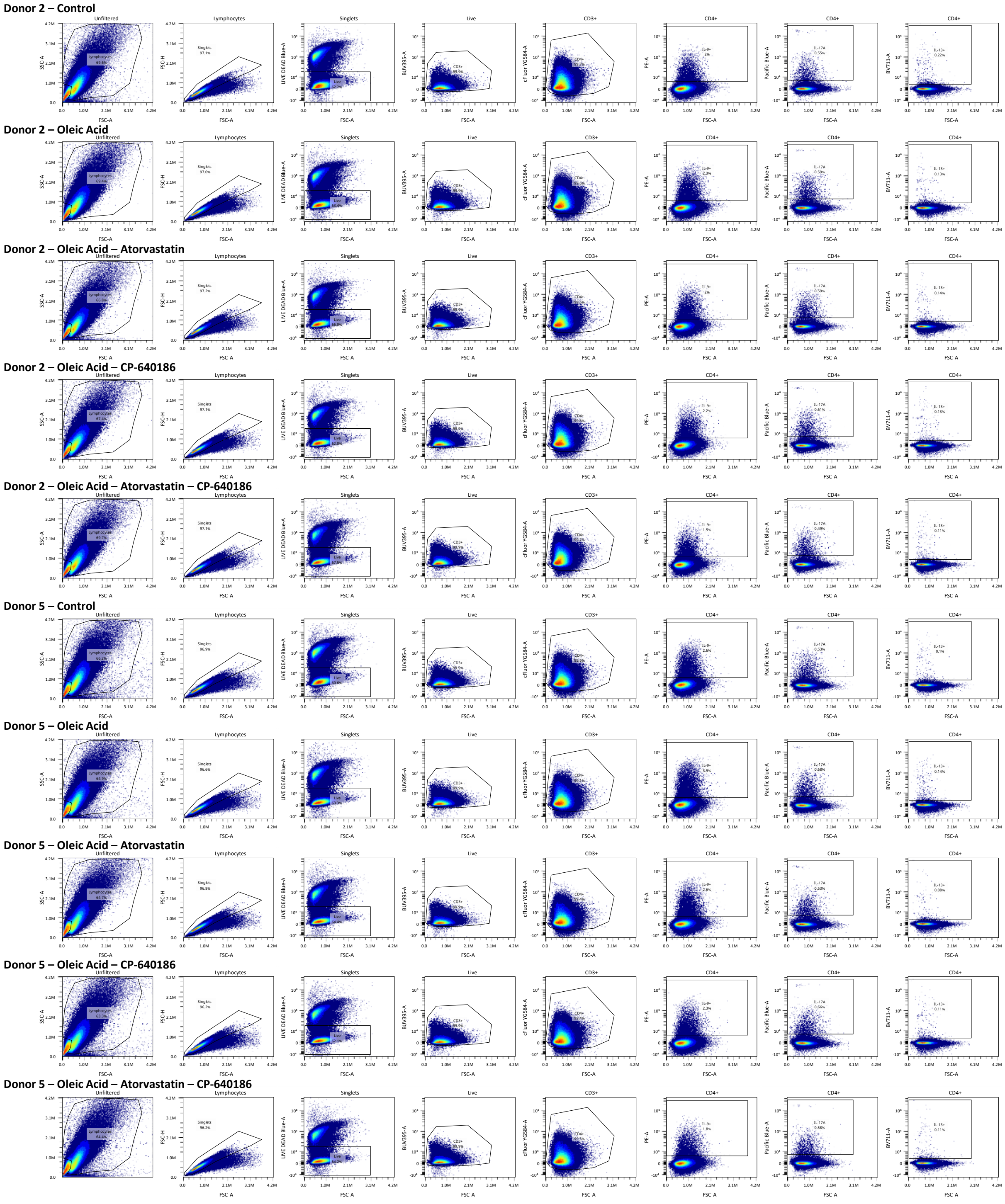

Data S17.2: Gating strategy for the inhibitor spectral cytometry analysis of activated CD4$^+$ T cells pre-exposed to oleic acid with or without metabolic inhibitors

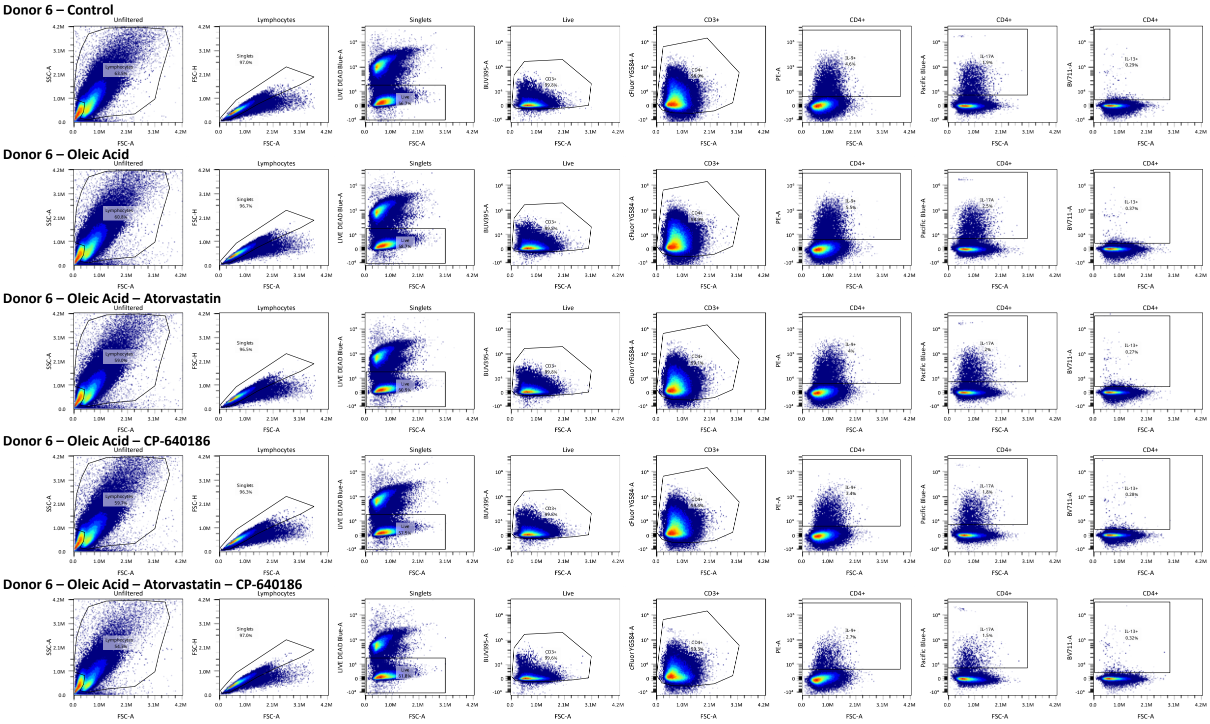

Supplemental Figure 17: Gating strategy for the inhibitor spectral cytometry analysis of activated CD4$^+$ T cells pre-exposed to oleic acid with or without metabolic inhibitors, n = 3, related to Figure 4. Gating strategy is the same for all 3 donors analyzed and includes gates set for all markers measured.
